# Three-way circulation–soil moisture–temperature feedbacks amplify the most extreme mid-latitudinal heat waves

**DOI:** 10.1126/sciadv.aeh9111

**Published:** 2026-11-06

**Authors:** Yinglin Tian, Jiangong Liu, Yu Huang, Pierre Gentine, Kai Kornhuber

**Affiliations:** ^1^ Potsdam Institute for Climate Impact Research (PIK), Member of the Leibniz Association, P.O. Box 6012 03, D-14412 Potsdam, Germany.; ^2^ Department of Earth and Environmental Engineering, Columbia University, New York, NY 10027, USA.; ^3^ Carbon Neutrality and Climate Change Thrust, Society Hub, The Hong Kong University of Science and Technology (Guangzhou), Guangzhou 511453, China.; ^4^ Max Planck Institute of Geoanthropology, Integrative Earth System Science, 07745 Jena, Germany.; ^5^ International Institute for Applied Systems Analysis, Laxenburg 2361, Austria.

## Abstract

The most extreme heat waves are intensifying faster than moderate events across many mid-latitude regions. Climate models underestimate these trends, suggesting an incomplete representation and understanding of the underlying physical processes. Here, we use an explainable machine-learning framework to investigate interactions among atmospheric circulation, surface soil moisture, and temperature, and their role in recent heat extremes. We show that a three-way interaction among these components becomes active once a critical temperature threshold is exceeded, contributing, on average, ∼10% to peak temperature anomalies during the most extreme heat events across ∼28% of humid mid-latitude regions. We further reveal that this interaction is systematically underestimated in CMIP6 and CESM2 Large Ensemble simulations. Soils in climate models are too dry at the initiation of heat waves, therefore suppressing subsequent soil-moisture depletion and inhibiting feedback activation. These findings help explain regional model biases in upper-tail warming and highlight avenues for improving future projections of extreme heat events.

## INTRODUCTION

Mega heat waves have become increasingly frequent and intense in recent decades, shattering local temperature records by unprecedented margins and causing widespread societal and ecological disruption (*1*, *2*). Notable examples include the 2003 European heat wave, which exceeded previous records by +2.4°C (*3*), the 2010 Russian event (+2.5°C) (*4*), the 2021 Pacific Northwest (PNW) heat wave (+4.6°C) (*5*, *6*), and multiple severe episodes during 2022–2023 across the UK, South America, Africa, and Asia (*7*, *8*). These events overwhelmed local adaptation capacities, causing more than 70,000 excess deaths in Europe in 2003 (*9*) and 60,000 in 2022 (*10*), alongside substantial impacts on ecosystems (*11*), agriculture (*12*), air quality (*6*), infrastructure (*13*), and economic activity (*14*). A critical scientific question is therefore raised: Are record-shattering heat waves merely statistical outliers in a warming climate, or do they reflect specific nonlinear processes that disproportionately intensify under global warming?

Beyond the rise in mean temperature that increases the likelihood of heat extremes (*15*), recent studies report a disproportionate acceleration of the most extreme temperatures relative to moderate heat events in certain regions (*16*, *17*), particularly over western Europe (*18*, *19*), while signals are less conclusive at global or hemispheric scales (*20*). This spatial heterogeneity suggests that changes in mean temperature alone cannot fully explain the observed regional intensification of record-breaking heat waves (*21*, *22*). A theoretical framework combining surface energy balance and evaporative fraction (EF) has been proposed to explain this nonlinear intensification, identifying net radiation and surface evaporative partitioning as two primary drivers (*20*, *23*). These two drivers are further linked to persistent anticyclonic flow anomalies (*21*, *24*) and strong land-atmosphere coupling (*25*), respectively. Regarding the latter, positive feedbacks between surface soil moisture and near-surface air temperature have been widely emphasized (*25*–*30*), that is, soil drying suppresses evaporative cooling, enhances sensible heat fluxes, and thus amplifies surface warming.

While soil moisture–temperature coupling is an established land-atmosphere mechanism in the context of extreme heat waves in current (*27*) and future climates (*25*), a potential three-way interaction among mid-tropospheric circulation (referred to as Z500), surface soil moisture (SM), and temperature (T), as highlighted during 2021 PNW heat wave (*5*), has not yet been systematically investigated across regions or events. Often, atmospheric circulation is treated solely as an external trigger of hot and dry conditions, rather than as a component that responds to land-surface anomalies (*25*). However, evidence from the 2003 and 2010 European heat waves indicates that soil drying can modify boundary-layer dynamics (*31*, *32*), pointing to a closed, positive Z500-SM-T feedback loop. Such a nonlinear multiway interaction could help explain why exceptional heat waves can occur even when both Z500 and SM anomalies are individually less extreme than the resulting temperature anomaly (*1*). In the context of the 2021 PNW heat wave, Bartusek *et al.* (*5*) further hypothesized a nonlinear behavior of the Z500-SM-T interaction and estimated it to contribute 40% of the corresponding exceptional heat. Yet, a comprehensive and quantitative assessment across multiple events and spatial scales remains lacking. Consequently, it remains unknown whether such nonlinear three-way interactions are a general feature of extreme heat, whether they preferentially emerge in particular climate regimes, and whether current climate models adequately capture the related processes.

Here, we address these questions using an explainable artificial intelligence (XAI) framework (Fig. 1) to diagnose nonlinear interactions among atmospheric circulation, soil moisture, and temperature across mid-latitude summer heat waves. Rather than relying on explainable machine learning (ML) alone, we integrate complementary algorithmic, statistical, and physical validations to evaluate the robustness and physical credibility of the diagnosed interactions. We show that the Z500-SM-T interaction is regionally threshold-activated: It remains weak below a critical temperature but becomes self-amplifying once a threshold is exceeded. This mechanism is prevalent across humid mid-latitude regions, where Z500-SM-T interactions are associated with the disproportionate intensification of the most extreme heat waves relative to moderate events. Moreover, these nonlinear interactions are consistently underestimated in current climate models, likely contributing to some of the most pronounced underestimations of extreme-heat trends globally. By further diagnosing the physical origins of this bias, we find that overly dry soil conditions at heat-wave onset suppress subsequent soil drying and feedback activation in models, implying that current projections may substantially underestimate the future intensification of the most extreme heat waves in regional hot spots.

**Fig. 1.**
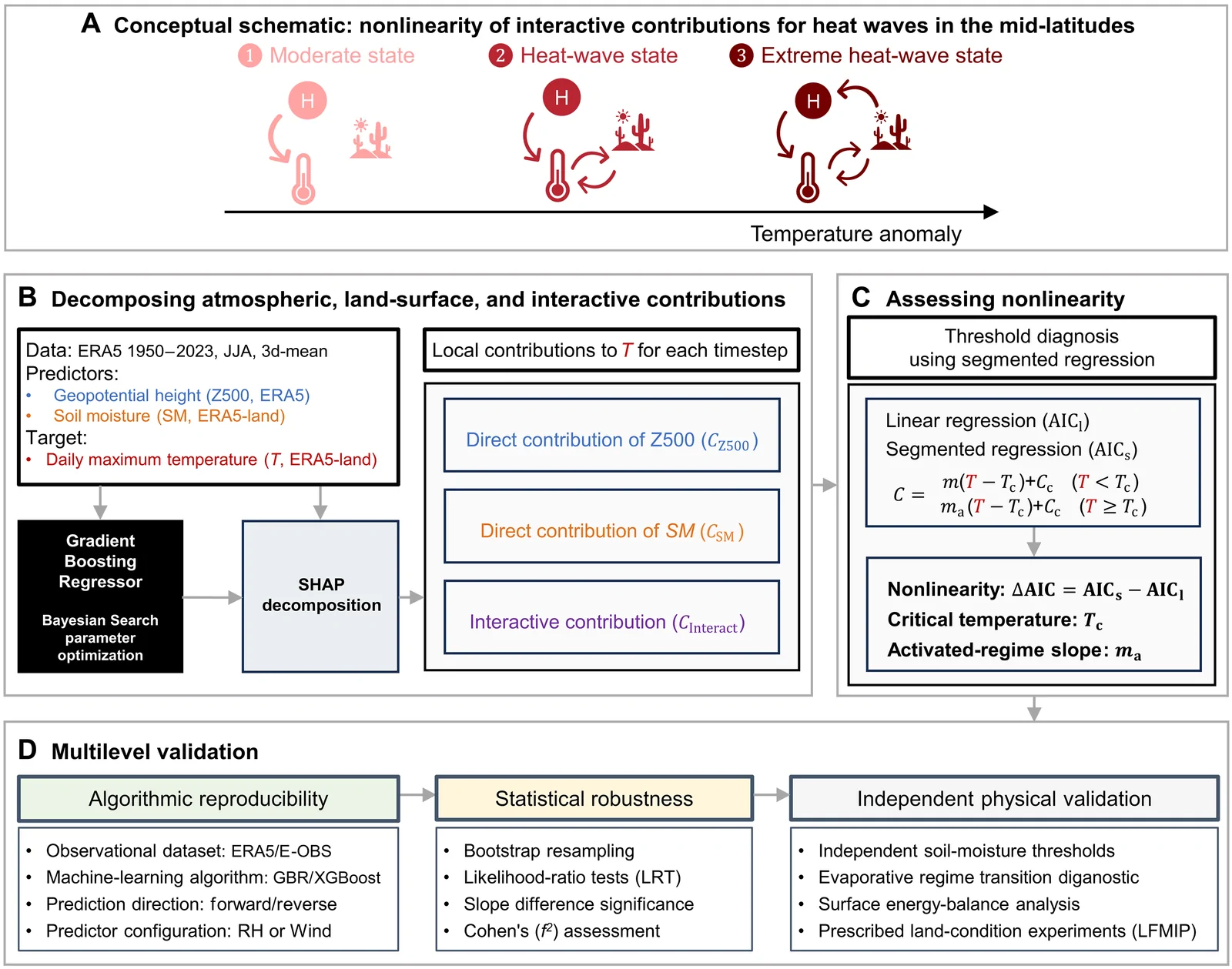
Conceptual schematic of the nonlinearity of the land-atmosphere interaction during the mid-latitude heat waves and the XAI framework. (**A**) Three different states during the evolution of heat waves in the mid-latitudes that vary by the temperature anomaly magnitude. The “H”, thermometer, and landscape icons indicate circulation, temperature, and soil moisture, respectively. Curved arrows indicate the direction of influence. (**B**) Overview of the explainable artificial intelligence (XAI) framework. A gradient boosting regression (GBR) model is trained using ERA5 and ERA5-Land reanalysis, with 3-day-mean daily maximum temperature (*T*) as the target variable and mid-tropospheric circulation [500-hPa geopotential height (Z500)] and near-surface soil moisture (SM) as predictors. Shapley additive explanations (SHAP) are then applied to decompose each predicted temperature anomaly into additive contributions from atmospheric circulation (*C*_Z500_), land-surface conditions (*C*_SM_), and their nonlinear interaction (*C*_interact_), thereby separating the interactive contribution from the direct effects of the individual drivers. 3d-mean, 3-day mean; JJA, June, July, and August. (**C**) Diagnosis of threshold-activated nonlinear amplification. The interaction contribution (*C*_interact_) is related to temperature using segmented regression. Nonlinearity is quantified by the difference in Akaike information criterion (AIC) between segmented and linear regressions (ΔAIC). For locations exhibiting robust nonlinear behavior, the critical temperature (*T*_c_) separating muted and activated interaction regimes and the activated-regime slope (*m*_a_) are used to characterize the onset and strength of nonlinear amplification. (**D**) Multilevel validation strategy for the proposed XAI framework from three complementary perspectives: (i) Algorithmic reproducibility assesses robustness across observational datasets, ML algorithms, prediction directions, and predictor configurations; (ii) statistical robustness evaluates the stability of the diagnosed nonlinear behavior using complementary statistical tests, including bootstrap resampling, likelihood-ratio tests, breakpoint slope significance tests, and the sample-size-independent Cohen’s *f*^2^ test; (iii) independent physical validation compares the XAI-diagnosed interaction with physically independent evidence from independently diagnosed soil-moisture thresholds, evaporative regime transition diagnostics, surface energy-balance analysis, and prescribed land-condition experiments from LFMIP experiments.

## RESULTS

### An explainable ML framework with multilevel validation

To investigate the dynamics of the most extreme heat waves, we develop an XAI framework that combines decision-tree–based ML with interpretable feature attribution to diagnose nonlinear interactions (Fig. 1). Compared with conventional statistical or process-based approaches, the XAI method captures nonlinear interactions directly from observations without imposing predefined functional forms while providing event-level attribution of the atmospheric, land-surface, and interactive contributions to extreme temperature anomalies (*33*–*36*). We focus on two well-established drivers of extreme heat in the extratropics (fig. S1) (*1*, *5*, *21*–*23*, *30*): mid-tropospheric high-pressure anomalies, represented by 500-hPa geopotential height (Z500), and near-surface soil moisture (SM). Z500 anomalies characterize variations in large-scale anticyclonic circulation and promote surface warming by enhancing incoming solar radiation and adiabatic heating associated with subsiding air masses (*37*, *38*), hereafter referred to as the direct contribution of circulation to temperature ($C_{Z500}$). SM anomalies quantify variations in land-surface water availability and modulate temperature by conditionally regulating evapotranspiration, thereby altering the partitioning of net radiation into latent versus sensible heat fluxes (*25*–*28*), hereafter referred to as the direct contribution of soil moisture to temperature ($C_{\text{SM}}$). When soil moisture is depleted and temperature reaches a sufficiently high level, the two drivers reinforce each other through a positive multivariate interaction: high-pressure systems intensify surface heating and soil drying, which, in turn, modifies surface energy partitioning and boundary-layer dynamics, enhancing upper-level blocking (fig. S1) (*31*, *32*). This nonlinear reinforcement manifests as the three-way circulation–soil moisture–temperature interaction (Fig. 1A), quantified as the Z500-SM-T interaction contribution ($C_{\text{Interact}}$).

The framework consists of two sequential steps (Fig. 1, B and C). First, a gradient boosting regression (GBR) model is trained using ERA5 and ERA5-Land reanalysis (1979–2023) (*39*, *40*), with the 3-day-mean daily maximum temperature ($T_{x}$) as the target and Z500 and SM as predictors (Fig. 1B; see Materials and Methods). We then apply Shapley additive explanations (SHAP) (*41*, *42*) to decompose predicted $T_{x}$ anomalies into additive contributions from $C_{Z500}$, $C_{\text{SM}}$, and their interaction $C_{\text{Interact}}$, defined as the component of the prediction that arises only when Z500 and SM act jointly (see Materials and Methods). In the second step, segmented regression is applied to quantify whether the interaction contribution exhibits threshold-dependent behavior during heat waves (Fig. 1C; see Materials and Methods). Nonlinearity is evaluated using the difference in Akaike information criterion (AIC) between segmented and linear regressions (∆AIC) (*43*, *44*), with ∆AIC<−2 reflecting a conservative preference for the segmented model. For locations satisfying this criterion in 99% of annual-replacement bootstrap realizations (see Materials and Methods), we further characterize the interaction using two key metrics: the critical temperature ($T_{c}$) separating muted and activated regimes, and the activated-regime slope ($m_{a}$), which quantifies the amplification rate beyond the threshold.

To establish confidence in the proposed XAI framework, we implement a hierarchical validation framework that evaluates the diagnosed nonlinear interaction (Fig. 1D). Rather than relying on a single diagnostic, each validation level addresses a distinct source of uncertainty, collectively evaluating the algorithmic reproducibility of the threshold-activated interaction, the statistical robustness of the nonlinearity diagnostics, and the physical consistency of the proposed land-air multivariate interaction. Accordingly, we organize the validation into three complementary levels:

1) Algorithmic reproducibility. We evaluate whether the diagnosed interaction depends on the observational dataset, ML algorithm, prediction direction, or predictor configuration by repeating the analyses using the independent E-OBS observational dataset (*45*), the XGBoost algorithm, reversed prediction direction with temporal lag between predictors and target variable, and expanded predictor sets including relative humidity (RH) and surface wind speed (see the “Algorithmic robustness across datasets, ML algorithms, and prediction directions” and “Robustness to predictor selection” sections).

2) Statistical robustness. We assess the robustness of the diagnosed threshold-dependent behavior using complementary statistical approaches, including bootstrap resampling, likelihood-ratio tests (*46*), segmented slope-difference tests (*47*, *48*), and the sample-size-insensitive metric Cohen’s *f*^2^ (*49*) (see the “Statistical robustness of nonlinearity assessment” section).

3) Physical consistency. We compare the XAI-diagnosed interaction with well-established land-atmosphere diagnostics, including independently diagnosed critical soil moisture (*50*), evaporative-regime transition assessment (*31*), surface energy-balance attribution (*51*), and prescribed land-condition experiments from Land Feedback Model Intercomparison Project (LFMIP) (*52*) (see the “Independent physical diagnostics” section).

### Threshold-dependent behavior of the Z500-SM-T interactions

To interpret the Z500-SM-T interactions and their threshold-dependent behavior diagnosed by the XAI-nonlinearity framework, we begin with Central Europe (CEU), a well-established heat-wave hot spot that has experienced some of the strongest observed increases in extreme summer temperatures (*1*, *16*–*19*, *23*, *24*). At the peak of the 2022 CEU heat wave (Fig. 2A), a recent record-breaking event, the Z500-SM-T interaction term ($C_{\text{Interact}}$) accounts for 13% [$C_{\text{Interact}}/\left(C_{Z500}+C_{\text{SM}}+C_{\text{Interact}}\right)$] of the predicted temperature $\left(T_{x}\right)$ anomaly (Fig. 2B; see Materials and Methods). By comparison, the direct contributions from Z500 ($C_{Z500}$) and SM ($C_{\text{SM}}$) contribute to 55 and 32%, respectively. Across the temperature peaks of the 10 strongest CEU heat waves over the past 45 years (1979–2023), the mean relative contributions are 65% from Z500, 25% from SM, and 10% from their interactions (fig. S2). Notably, $C_{\text{Interact}}$ increases sharply during the hottest phases of heat-wave evolution, particularly near temperature peaks (fig. S3), suggesting a threshold-dependent behavior. To quantify this behavior, we regress $C_{\text{Interact}}$ against $T_{x}$ and find that a segmented regression provides a statistically better description than a linear regression (Fig. 2C), with $∆{\text{AIC}}_{\text{interact}}=-23.2$ [99% confidence interval (CI) from annual-replacement bootstrapping: −33.6 to −16.1; see Materials and Methods]. The nonlinearity of the direct contributions ($C_{Z500}$ and $C_{\text{SM}}$) is comparatively lower, with $∆{\text{AIC}}_{Z500}=0.1$ (99% CI: −2.6, 2.3) and $∆{\text{AIC}}_{\text{SM}}=-2.9$ (99% CI: −6.9, 1.2) (fig. S4). Using a deliberately conservative criterion that requires the entire 99% bootstrap CI to satisfy ∆AIC<−2, robust nonlinearity is retained exclusively for the interaction contribution. This segmented relationship between $C_{\text{Interact}}$ and $T_{x}$ identifies a critical temperature threshold ($T_{c}=298.1$ K), corresponding to 3.8 K above the multiyear summer mean. Once this threshold is exceeded, $C_{\text{Interact}}$ intensifies at the rate of 0.10 K K^−1^, hereafter referred to as the activated-regime slope ($m_{a,\text{interact}}$ or $m_{a}$ for simplicity, as only $C_{\text{Interact}}$ shows robust nonlinearity), indicating a transition from a muted to an activated interaction regime.

**Fig. 2.**
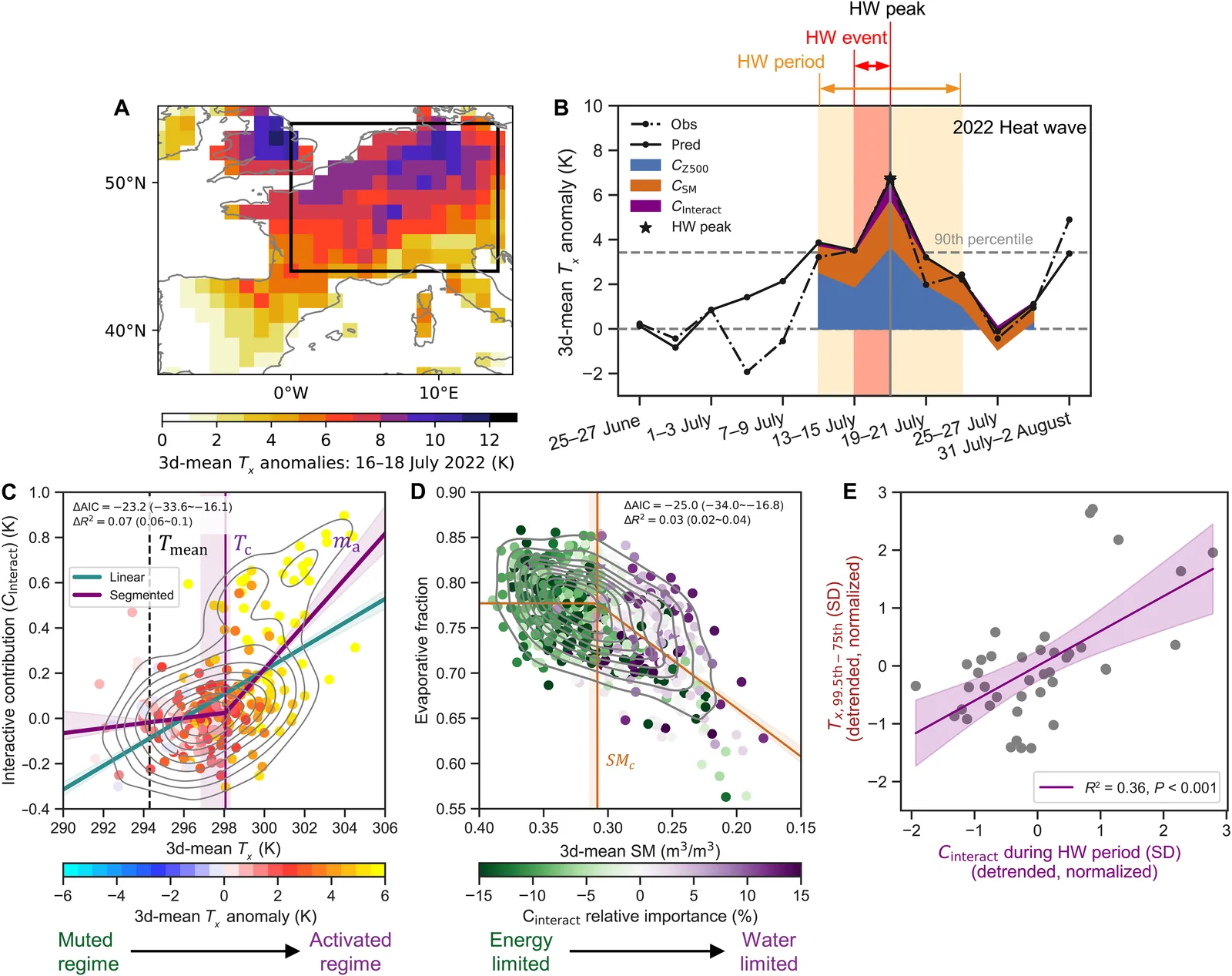
Role of Z500-SM-T interactions in amplifying the most extreme heat waves in Central Europe. (**A**) Spatial distribution of anomalies of the 3-day-mean daily maximum temperature ($T_{x}$) at the peak of the 2022 Central European (CEU) heat wave, relative to the 1979–2023 calendar climatology. (**B**) XAI model attribution during the 2022 CEU heat-wave period, showing observed (dashed) and predicted (solid) $T_{x}$ anomaly and their decomposition into contributions from mid-tropospheric geopotential height ($C_{Z500}$, blue), surface soil moisture ($C_{\text{SM}}$, orange), and their interactions ($C_{\text{Interact}}$, purple). The heat-wave period is defined as days with positive observed $T_{x}$ anomaly before and after the heat-wave peak (yellow horizontal span; see Materials and Methods). HW, heat wave. (**C** to **E**) Regression analyses over 1979–2023 summers: (C) segmented and linear regression between $C_{\text{Interact}}$ and $T_{x}$ during all heat-wave periods; (D) segmented regression between surface soil moisture (SM) and EF during all dry-down periods (see Materials and Methods); (E) linear regression between the upper tail width of temperature distribution ($T_{x,99.5\text{th}}-T_{x,75\text{th}}$) and the mean $C_{\text{Interact}}$ during heat-wave periods, with both axes calculated using a 5-year moving window and subsequently detrended and normalized. In (C) to (E), contours indicate the kernel density estimation (levels from 0.1 to 0.9) and shaded envelopes denote the 99% confidence interval (CI) of the fitted regression (see Materials and Methods); ∆AIC and $∆R^{2}$ represent performance differences between the segmented and linear regression, with 99% CIs (LCI − UCI); $T_{\text{mean}}$ denotes the multiyear mean summer $T_{x}$; $T_{c}$ is the critical temperature separating muted and activated interaction regimes; $m_{a}$ is the activated-regime slope of the $C_{\text{Interact}}-T_{x}$ relationship; and ${\text{SM}}_{c}$ is the critical soil-moisture threshold marking the transition from energy- to water-limited evaporation. 3d-mean, 3-day mean.

The diagnosed threshold behavior of the interactive term is robust across complementary validation analyses from both algorithmic and statistical perspectives (figs. S4 to S9; see Materials and Methods). The segmented relationship of $C_{\text{Interact}}-T_{x}$ is consistently reproduced across independent observational datasets, ML algorithms, reversed prediction directions with the temporal lag between predictors and target variable, and expanded predictor configurations including surface wind speed and RH (figs. S4 to S8; see Materials and Methods). Independent statistical assessments further support the segmented relationship (fig. S9; see Materials and Methods). Comparable numbers of observations occur below and above the identified threshold, indicating that the detected transition is not driven by sample imbalance. Bootstrap resampling further confirms the stability of the estimated threshold and segmented relationship, demonstrating that the results are not unduly influenced by a small number of exceptional heat waves. Last, likelihood-ratio tests (*46*) and segmented slope-difference tests (*47*, *48*) consistently favor the segmented model over the linear alternative (both *P* < 0.01; see Materials and Methods).

To understand the physical origin of the identified threshold, we compare the XAI diagnosis with three independent lines of physical evidence (Fig. 1C). First, an independently diagnosed critical soil-moisture threshold (${\mathrm{SM}}_{c}$ = 0.31 m^3^ m^−3^) is identified from the transition between energy-limited and water-limited evaporation using the relationship between EF and soil moisture during summertime dry-down periods (Fig. 2D; see Materials and Methods) (*50*). The independently derived $T_{c}$ and ${\text{SM}}_{c}$ intersect when projected onto the linear summer-wide relationship between temperature and soil moisture (fig. S10). Consistently, potential evaporation anomalies increase more rapidly beyond $T_{c}$, whereas actual evaporation continues at a similar or reduced rate (fig. S10), indicating a shift in surface energy partitioning toward enhanced sensible heating and activation of land-atmosphere interactions (*31*, *32*). Second, decomposition based on the surface energy balance (*51*) reproduces the relative magnitudes of the atmospheric and land-surface contributions diagnosed by SHAP (fig. S11; see Materials and Methods), providing physical support for the marginal contributions. Last, we perform an independent process-based assessment using the prescribed-soil-moisture simulations from LFMIP-pdLC experiment (*52*). Applying the XAI framework to the LFMIP-pdLC simulations reveals a substantially weakened interaction together with greatly diminished evidence for its threshold-dependent behavior (fig. S12; see Materials and Methods). Collectively, these independent physical diagnostics provide evidence that the identified threshold-activated interaction reflects a physically meaningful land-atmosphere feedback rather than statistical covariance alone. At the same time, none of these physical approaches can explicitly separate the nonlinear interaction from the direct effects of atmospheric circulation and soil moisture, highlighting the unique capability of the XAI framework to isolate and quantify the multivariate interaction.

Under continued warming, the upper tail of the summer temperature distribution in CEU broadens substantially, quantified as the difference between the 99.5th and 75th percentiles using a 5-year moving window (fig. S13). During the same period, the exceedance probability of the identified critical temperature increases from 6% (1979–1983) to 23% (2018–2022), closely tracking the rising probability of SM falling below ${\text{SM}}_{c}$. Consequently, an increasing fraction of summer days operates within the activated interaction regime, accompanied by a significant strengthening of $C_{\text{Interact}}$ during heat waves (*P* < 0.01) and a 25% expansion of regions exhibiting nonlinear interactions during 2004–2023 relative to 1979–1998 (figs. S14 and S15). We show that the mean interaction contribution during heat-wave periods explains a substantial fraction of the interannual variability in the upper-tail width of the summer temperature distribution [coefficient of determination (*R*^2^) of 0.36, *P* < 0.01; Fig. 2E], exceeding the explanatory power of either the direct circulation contribution (*R*^2^ of 0.14) or the direct soil-moisture contribution (*R*^2^ of 0.30; fig. S14).

### Three-way interactions amplify the most extreme heat waves across humid mid-latitudes when passing critical thresholds

Beyond CEU, extreme summer temperatures have intensified across many mid-latitude regions, at a faster rate than the moderate heat events (*1*, *16*, *17*). Extending our regional analysis to mid-latitudes, we identify widespread but spatially heterogeneous threshold-activated Z500-SM-T interactions during summer heat-wave periods over 1979–2023 (Fig. 3A). Under a conservative model-selection criterion (ΔAIC < −2), ∼15% of mid-latitude land areas exhibit robust nonlinear interactions. These hot spots are concentrated in Central and Eastern Europe, southern Russia and Mongolia, eastern and northwestern North America, northwestern India, the western coast of South America, central and southern Africa, and northern interior Australia. Within regions exhibiting robust nonlinear interaction ($∆{\text{AIC}}_{\text{interact}}<-2$, confirmed in ≥99% of annual-replacement bootstrap realizations; figs. S16 and S17; see Materials and Methods), the Z500-SM-T interaction contributions account for a nonnegligible fraction, on average, 10%, of peak $T_{x}$ anomalies during the heat-wave events (fig. S18). The diagnosed critical temperature threshold averages 4.0 ± 1.6 K (spatial mean ± SD) above the local summer climatology, closely corresponding to independently diagnosed critical soil-moisture thresholds of ∼0.32 m^3^ m^−3^ (fig. S19). Above this threshold, Z500-SM-T interaction strengthens rapidly, with an activated-regime slope of $m_{a}$= 0.1 ± 0.04 K K^−1^ (spatial mean ± SD; Fig. 3B), whereas the direct circulation and soil-moisture contributions remain largely linear across the mid-latitudes (fig. S20). Moreover, regions exhibiting robust threshold-activated interactions coincide with areas showing the largest increases in the most extreme temperatures (1-in-200-year $T_{x}$), quantified by the 99.5th-percentile summer temperatures during 2003–2023 relative to 1979–1999 (Fig. 3C), especially over the Northern Hemisphere mid-latitudes (40°N to 60°N) (Fig. 3D).

**Fig. 3.**
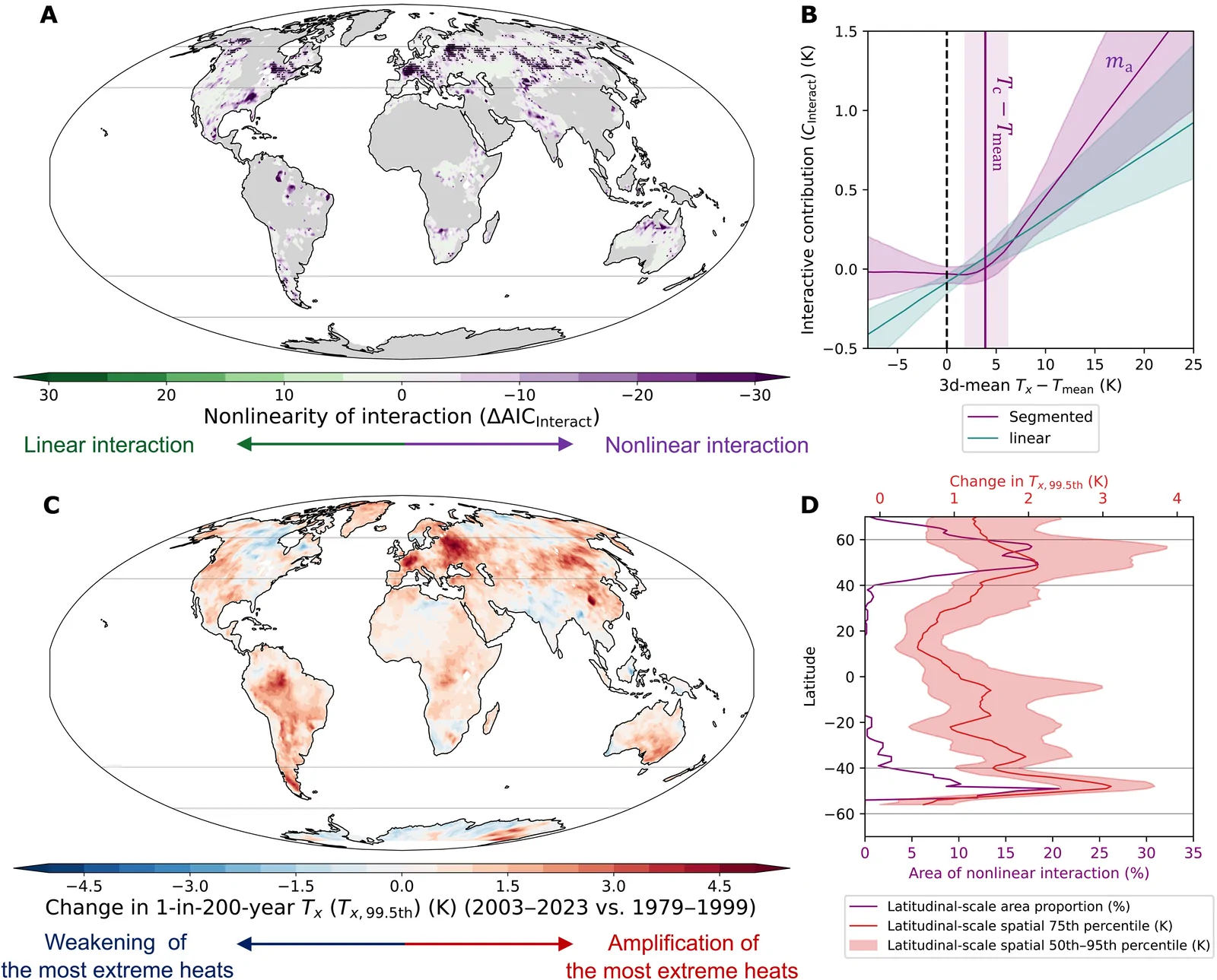
Global patterns of threshold-activated Z500-SM-T interactions and intensification of the most extreme heats. (**A**) Spatial distribution of the nonlinearity of the Z500-SM-T interaction, quantified by $∆{\text{AIC}}_{\text{interact}}$, during summer heat-wave periods over 1979–2023. Black dots indicate grid points exhibiting robust nonlinear behavior, defined by $∆{\text{AIC}}_{\text{interact}}<-2$ and confirmed in ≥99% of annual-replacement bootstrap realizations (see Materials and Methods). Gray shading denotes regions where the ML model explains less than 60% of local temperature variance ($R_{\text{ML}}^{2}<0.6$; see Materials and Methods). (**B**) Spatially composited relationship between the interactive contribution ($C_{\text{Interact}}$) and local temperature ($T_{x}$) anomaly at grid points exhibiting robust nonlinear behavior [black dots in (A); details in fig. S16]. Solid lines show the spatial median response, and shaded envelopes indicate the 10th- to 90th-percentile range across locations. Temperature anomalies are expressed relative to the local summer mean. 3d-mean, 3-day mean. (**C**) Change in the 99.5th-percentile summer temperature ($T_{x,99.5\text{th}}$) between 2003–2023 and 1979–1999. (**D**) Latitudinal distribution of the prevalence of the threshold-activated Z500-SM-T interaction (purple) and the magnitude of the changes in $T_{x,99.5\text{th}}$ (red); shaded envelopes indicate the 50th- to 95th-percentile spatial range. In (A) to (D), gray horizontal lines mark the mid-latitude band (40°N/S to 60°N/S).

To understand the climatic controls on this spatial variability, we stratify both extreme-temperature changes and interaction characteristics according to the aridity index [AI = actual/potential evapotranspiration; (*53*)] across the Northern Hemisphere mid-latitudes. Only grid points where the ML model reliably reproduces local temperature variability ($R_{\text{ML}}^{2}>0.6$, see Materials and Methods) are included. Humid regions (AI = 0.65 to 1.0) exhibit both the largest increases in upper-tail temperatures (Fig. 4A) and the strongest nonlinear interactions (Fig. 4B), with around 28% of humid land areas exhibiting threshold-dependent behavior under the conservative criterion (table S1). Alternative model-selection criteria, bootstrap resampling strategies, likelihood-ratio tests, segmented slope-difference tests, Cohen’s *f*^2^ test consistently identify similar hot spot regions (figs. S16 and S17; see Materials and Methods). Depending on the statistical criterion adopted, 15 to 30% of humid mid-latitude land areas exhibit robust threshold-activated interactions (table S1), demonstrating that the identified spatial pattern is insensitive to statistical methodology.

**Fig. 4.**
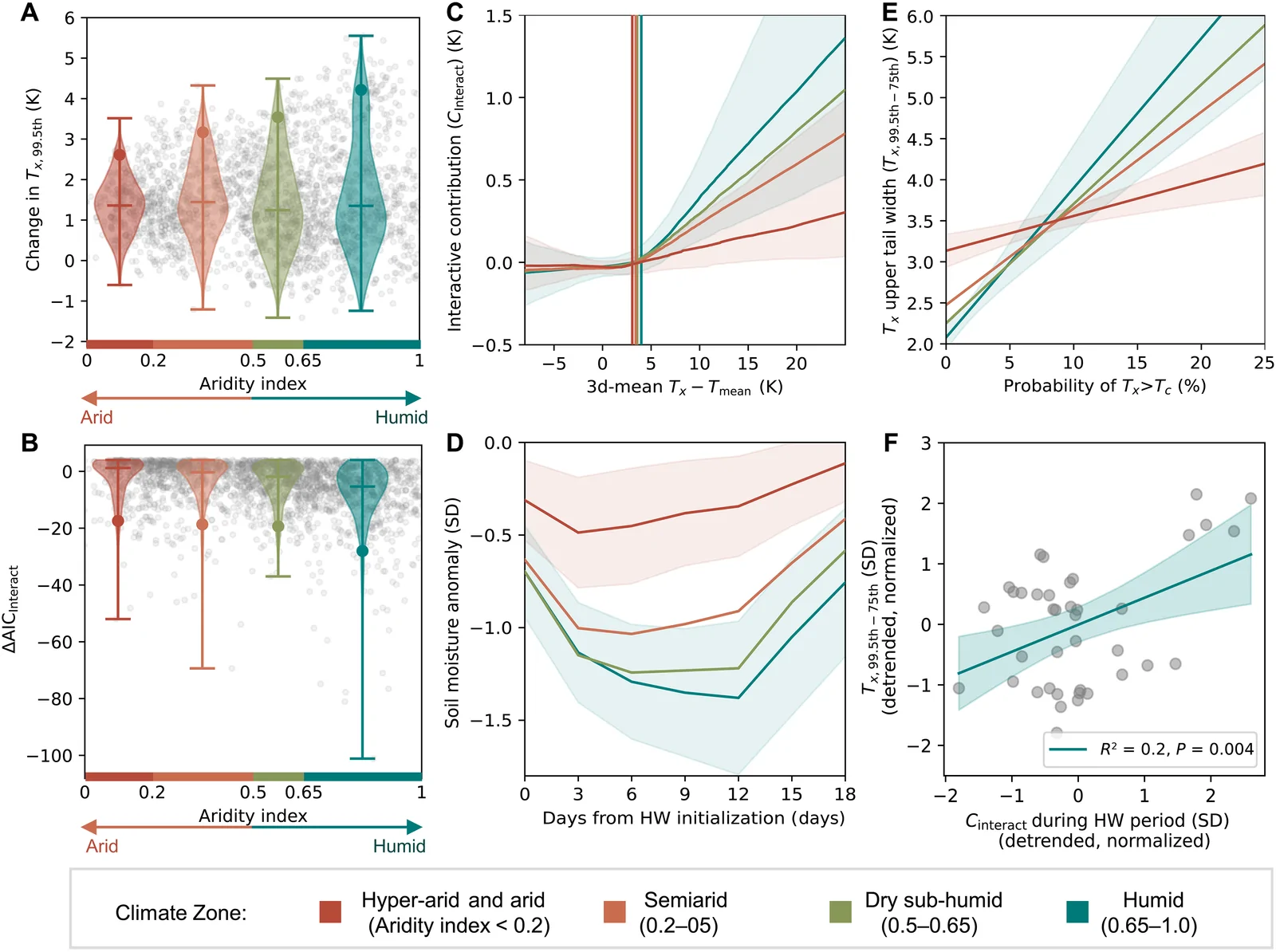
Threshold-activated Z500-SM-T interactions vary across climate zone, with strongest effects in mid-latitude humid regions. (**A**) Changes in the 99.5th-percentile summer temperature ($T_{x,99.5\text{th}}$) between 2003–2023 and 1979–1999 across Northern mid-latitude (40°N to 60°N), stratified by aridity zone and shown as violin plots. (**B**) Nonlinearity of the Z500-SM-T interaction ($∆{\text{AIC}}_{\text{interact}}$), during summer heat-wave periods over 1979–2023, stratified by aridity zone and shown as violin plots. In (A) and (B), gray points indicate all land grid points; violins represent probability density, with solid lines denoting the median, minimum, and maximum and with colored circles indicating the 95th/5th percentile. (**C** to **E**) Spatially composited relationships by aridity zone for summers over 1979–2023: (C) segmented regression between the interactive contribution ($C_{\text{Interact}}$) and local temperature ($T_{x}$) anomaly during heat-wave periods; (D) changes of standardized soil-moisture anomalies following heat-wave onsets; (E) linear regression between the upper tail width of temperature distribution ($T_{x,99.5\text{th}}-T_{x,75\text{th}}$) and the exceedance probability of the critical temperature threshold (probability of $T_{x}>T_{c}$), calculated using a 5-year-moving window. In (C) to (E), shading represents the spatial extent from the 10th to 90th percentiles for the most humid and the most arid zones, with spatial spread for other zones provided in fig. S21. 3d-mean, 3-day mean. (**F**) Linear regression between the upper tail width of temperature distribution ($T_{x,99.5\text{th}}-T_{x,75\text{th}}$) and the mean $C_{\text{Interact}}$ during heat-wave periods in mid-latitude humid regions [aridity index (AI), 0.65 to 1.0]. Both axes are calculated using a 5-year moving window and subsequently detrended and normalized; shaded envelope indicates the 99% CI of the fitted regression. Note that only the locations with $R_{\text{ML}}^{2}>0.6$ are taken into account for all panels.

This spatial pattern reflects contrasting soil-moisture dynamics across climate regimes. Although the critical temperature threshold remains relatively constant across climate regimes (∼4 K above the local climatology), the activated-regime slope varies systematically with aridity, reaching its maximum in humid regions before progressively weakening toward drier climates (Fig. 4C). In humid environments, evapotranspiration is typically energy-limited under normal summer conditions but shifts abruptly to water limitation following intense soil-moisture depletion during prolonged heat waves (*54*). Consequently, humid regions experience the most rapid and pronounced soil-moisture decline during heat-wave evolution, with a spatially averaged reduction of 1.4 SDs within ∼12 days, thereby leading to a stronger interaction activation once the threshold is exceeded (Fig. 4D and fig. S21). As a result, the relationship between the exceedance frequency of the diagnosed critical temperature and upper-tail temperature widening is strongest in humid climates (Fig. 4E). Across mid-latitude humid regions, the upper-tail width of summer temperature distributions has increased significantly over time (*P* < 0.05), with ∼20% of its interannual variance associated with the strength of the Z500-SM-T interaction during heat-wave periods (*R*^2^ = 0.2, *P* < 0.01; Fig. 4F). In comparison, analogous associations with the direct Z500 and SM contributions are substantially weaker (*R*^2^ = 0.11 and 0.03, respectively; fig. S22). These results suggest that ongoing warming increasingly shifts summer temperatures into a regime where nonlinear land-atmosphere interactions preferentially amplify the most extreme heat events in humid mid-latitudes.

### Underestimation of three-way interactions in models linked to biased soil-moisture dynamics

Given the strong observational evidence that the threshold-activated Z500-SM-T interactions amplify the most extreme heat waves, especially in humid mid-latitude regions, we assess whether climate models can reproduce these dynamics. This evaluation is critical for understanding the credibility of future extreme-heat projections. We analyze simulations from 12 CMIP6 models alongside a 40-member CESM2 Large Ensemble (CESM2-LE2) (*55*, *56*), with the ML model separately trained on each CMIP6 and CESM2-LE2 simulations (see Materials and Methods). Our analysis reveals that CMIP6 models consistently underestimate the regional nonlinearity of Z500-SM-T interactions relative to ERA5 (Fig. 5, A and B). Across the hot spots identified in observations (fig. S16; see Materials and Methods), more than 90% of CMIP6 models fail to reproduce the observed regime-shifting behavior (Fig. 5A and fig. S23), with the magnitude of $∆{\text{AIC}}_{\text{interact}}$ undervalued by 93 ± 10% (model mean ± SD) (fig. S24). Although models generally capture the critical temperature threshold $T_{c}$, they substantially underestimate the activated-regime slope $m_{a}$, with mean reductions of 69 ± 10% (Fig. 5B and fig. S24). Analysis of the CESM2-LE2 ensemble confirms that this bias is structural rather than driven by internal variability: the observed activated-regime response lies entirely outside the model range even considering the ensemble range (Fig. 5B).

**Fig. 5.**
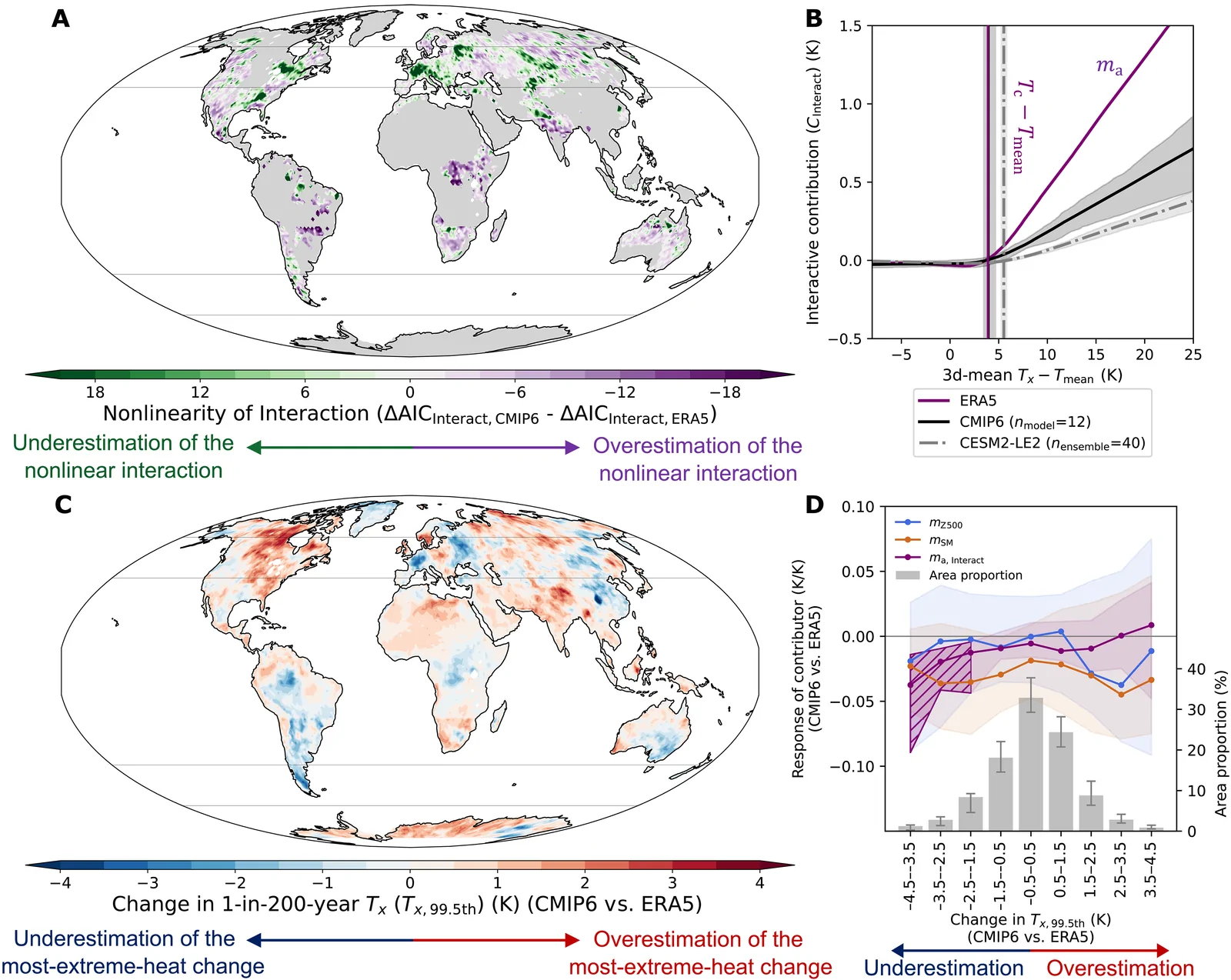
Underestimated nonlinearity of Z500-SM-T interactions as one contributor to biases in simulated extreme-heat changes. (**A**) Difference between the CMIP6 multimodel mean and ERA5 in the nonlinearity of the Z500-SM-T interaction ($∆{\text{AIC}}_{\text{interact}}$) during summer over 1979–2023. Gray shading denotes regions where the ML model explains less than 60% of local temperature variance in observation ($R_{\text{ML}}^{2}<0.6$; see Materials and Methods). (**B**) Regression between the interactive contribution ($C_{\text{Interact}}$) and local temperature anomaly ($T_{x}$) in ERA5 (purple, solid), CMIP6 multimodel mean (dark gray, solid), and CESM2-LE2 ensemble mean (light gray, dashed), evaluated at grid points exhibiting robust nonlinear behavior in observation ($∆{\text{AIC}}_{\text{interact}}<-2$ and confirmed in ≥99% of annual-replacement bootstrap realizations; see Materials and Methods). 3d-mean, 3-day mean. (**C**) Difference between ERA5 and the CMIP6 multimodel mean in changes of the 99.5th-percentile summer temperature ($T_{x,99.5\text{th}}$) between 2003–2023 and 1979–1999. (**D**) Model bias in response of the three investigated contributors ($m_{Z500},\;m_{\text{SM}}$, and $m_{a,\text{interact}}$; see Materials and Methods) across grid points where ML model explains more than 60% of local temperature variance ($R_{\text{ML}}^{2}>0.6$), identified for each model separately. The histogram below shows the CMIP6 multimodel mean area fraction within bins of biases in simulated $T_{x,99.5\text{th}}$ change (from −4.5- to −3.5-K underestimation to +3.5- to +4.5-K overestimation), defined relative to ERA5; error bars indicate the intermodel range (10th to 90th percentiles). In (B) and (D), solid lines denote multimodel or multiensemble mean, and shaded envelopes show intermodel or interensemble spread of 10th- to 90th-percentile range. The purple hatched shaded envelope in (D) indicates the model spread does not overlap with zero.

Regions where models underestimate interaction nonlinearity also show underestimated increases in $T_{x,99.5\text{th}}$ (Fig. 5C). To attribute model biases in extreme-temperature trends to individual contributors, we compare the temperature sensitivity of each contribution ($m_{Z500}$, $m_{\text{SM}}$, and $m_{a,\text{interact}}$) in CMIP6 models against ERA5 (Fig. 5D; see Materials and Methods). Mid-latitude grid points with reliable ML performance (${R^{2}}_{\text{ML}}>0.6$) are grouped into nine categories on the basis of model bias in simulated changes of $T_{x,99.5\text{th}}$, ranging from strong underestimation (−4.5 to −3.5 K) to strong overestimation (+3.5 to +4.5 K). These categories are defined separately for each model, with their areal fractions shown in the histogram of Fig. 5D. Results indicate that CMIP6 models exhibit no systematic bias in the response of mid-tropospheric geopotential height ($m_{Z500}$), showing the largest intermodel spread for this contributor. By contrast, the soil-moisture response ($m_{\text{SM}}$) tends to be underestimated when averaged across the mid-latitudes, although this bias does not exhibit strong intermodel agreement. While the magnitude of the interaction bias ($m_{a,\text{interact}}$) is smaller than that of the soil-moisture bias when averaged over all mid-latitudes, the interaction term shows a unique behavior: an underestimation of the interaction is found specifically where heat-wave trend biases are largest (−4.5 to −1.5 K). Over these locations, we find a ∼90% intermodel agreement in the sign of the interaction bias (see hatched envelope in Fig. 5D). This pattern indicates that Z500-SM-T interaction plays an important role in shaping model biases in the most extreme heat regimes, likely linked to deficiencies in simulated soil-moisture dynamics. Nevertheless, the substantial intermodel spread, particularly in the circulation and soil-moisture responses, and the presence of regions with excessive warming underscore the need for further investigation using process-based diagnostics and more model-specific analyses.

To diagnose the physical origin of this bias, we examine soil-moisture dynamics during heat waves, focusing on CEU where observed regime shifts are pronounced but poorly represented in models (Fig. 6A). In ERA5, soil moisture at heat-wave onset averages ∼0.30 m^3^ m^−3^ and declines rapidly at a rate of ∼2.4% day^−1^ (quantified via evaporative efficiency $E_{\text{ET}}$; see Materials and Methods). CMIP6 models instead simulate drier initial conditions (∼0.20 ± 0.03 m^3^ m^−3^; model mean ± SD; fig. S25) and a slower subsequent drying rate (1.4 ± 0.7% day^−1^; Fig. 6B). Across models, slower soil-moisture decline rates are associated with weaker interaction nonlinearity ($∆{\text{AIC}}_{\text{interact}}$) [correlation coefficient (*r*) = −0.65, *P* = 0.02; Fig. 6C] and reduced activated-regime slopes $m_{a}$ (*r* = 0.71, *P* < 0.01; fig. S25). In addition, soil-moisture decline rates correlate positively with initial soil moisture at heat-wave onset (*r* = 0.62, *P* = 0.02; Fig. 6D), indicating that overly dry starting conditions impose strong water limitations on subsequent evaporation.

**Fig. 6.**
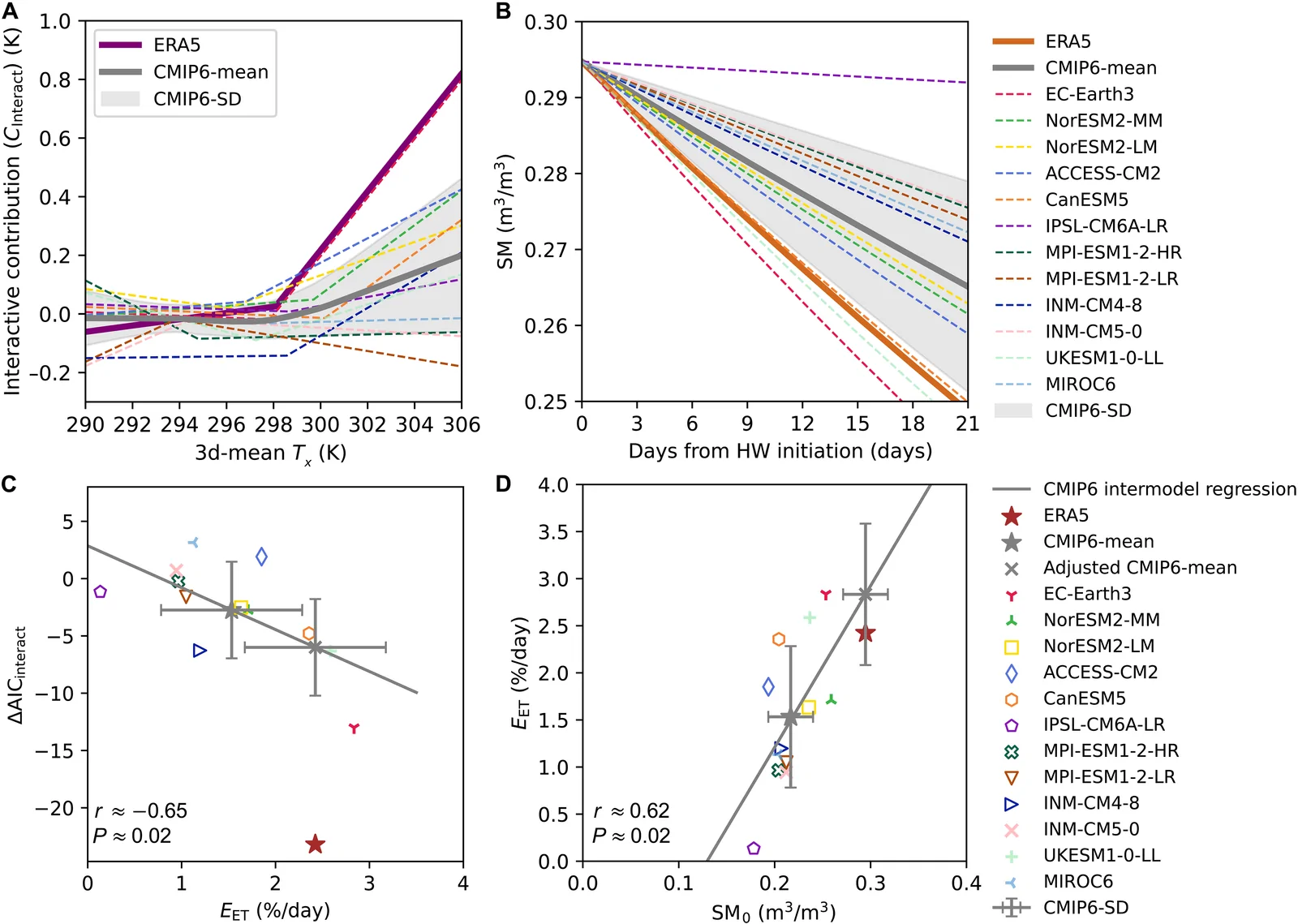
Premature soil drying and overly slow subsequent drying contribute to the underestimation of nonlinear Z500-SM-T interaction in climate models. (**A**) Nonlinear relationship between the Z500-SM-T interaction ($C_{\text{Interact}}$) and local temperature ($T_{x}$) during summer heat-wave periods (1979–2023) over CEU, shown for ERA5 and 12 CMIP6 models. 3d-mean, 3-day mean. (**B**) Soil-moisture decline rate within 21 days following heat-wave onset (1979–2023) in ERA5 and 12 CMIP6 models over CEU (see Materials and Methods), calculated assuming a common observed initial soil moisture at heat-wave onset. In (A) and (B), gray lines show CMIP6 multimodel mean and shaded envelopes indicate intermodel variability (±1 SD). (**C** and **D**) Intermodel relationships during summer heat-wave periods (1979–2023) over CEU: (C) regression between the soil-moisture decline rate within 21 days after heat-wave onset ($E_{\text{ET}}$) and nonlinearity of the Z500-SM-T interaction ($∆{\text{AIC}}_{\text{Interact}}$); (D) regression between the initial soil moisture at heat-wave onset (${\text{SM}}_{0}$) and the subsequent 21-day soil-moisture decline rate ($E_{\text{ET}}$). The adjusted CMIP-mean represents the value estimated from the intermodel regression across CMIP6 models, using the corresponding ERA5 value as the predictor.

## DISCUSSION

Using an explainable ML framework, we identify a three-way interaction among atmospheric circulation, soil moisture, and surface air temperature that becomes active beyond critical temperature and soil-moisture thresholds and specifically contributes to the magnitude of the most extreme heat waves across humid mid-latitude regions. Across the identified hot spots, this interaction contributes ∼10% of peak heat-wave intensity. It becomes active once temperature exceeds a critical threshold of ∼4 K above the local summer climatology, corresponding closely to an independently diagnosed critical soil-moisture threshold of ∼0.32 m^3^ m^−3^. Under continued warming, exceedance of this threshold has become increasingly frequent, allowing more summer days to enter the activated interaction regime. Consequently, the nonlinear interaction explains ∼20% of the observed upper-tail widening of summer temperature distributions across humid mid-latitudes and up to 36% in CEU, where its spatial extent has expanded by 25% over recent decades.

One of the principal conceptual advances of this study is extending the well-established pairwise soil moisture–temperature framework (*25*, *26*) to a threshold-activated three-way interaction among atmospheric circulation, soil moisture, and temperature that is consistently observed across heat-wave events and regions (Fig. 1 and fig. S1). By explicitly incorporating circulation into the interaction process, we reveal that the strongest nonlinearity resides in the interaction, whereas the direct contributions of circulation and soil moisture remain largely linear during the evolution of heat waves (Fig. 2 and fig. S4). This identified threshold-activated interaction provides a potential explanation for why record-breaking heat has intensified particularly rapidly in humid mid-latitude regions (Fig. 3) (*1*, *16*). In these regions, soils are substantially wetter before heat-wave onset and can therefore sustain much larger soil-moisture deficits as heat waves intensify (fig. S21). Consequently, although mean land-atmosphere coupling is comparatively weak under normal summer conditions, once the critical threshold is exceeded, the interaction strengthens more rapidly and reaches greater magnitudes in humid regions than in drier climates, producing stronger nonlinear amplification (Fig. 4). With continued warming, temperatures in humid regions increasingly exceed this critical threshold, allowing more heat waves to enter the activated interaction regime (fig. S22). Record-shattering heat waves may therefore arise not simply from the overall warming trend (*15*), but increasingly from nonlinear circulation–soil moisture–temperature interactions that disproportionately amplify the most extreme events.

The identified threshold-activated interaction also provides a complementary view of the climate-model biases in heat and dryness simulation (*16*). Previous studies have primarily attributed model deficiencies in hot-dry extremes to insufficient soil-moisture depletion in dry and semiarid regions (*57*), largely driven by the systematic overestimation of humidity (*58*). Our results suggest a distinct bias mechanism operating in humid climates. Compared with observations, CMIP6 models tend to simulate soils that are already excessively dry at heat-wave onset, leaving insufficient available soil moisture for the strong depletion observed during the hottest stages of heat waves (Fig. 6). This bias likely reflects an overemphasis on water limitation under moderate conditions in models, potentially linked to simplified representations of vegetation rooting depth, subgrid heterogeneity, and soil-moisture buffering processes (*59*, *60*). Consequently, the transition into the activated interaction regime is reached prematurely and substantially weakened, suppressing the nonlinear amplification of extreme temperatures (Fig. 5). The model deficiency in humid climates is therefore not simply the climatological soil-moisture state but also the timing of soil-moisture depletion relative to heat-wave evolution.

Several limitations should nevertheless be acknowledged. First, the identified Z500-SM-T interaction represents one important amplification pathway rather than a universal mechanism governing all regional heat extremes. For example, western North America, eastern Australia, and parts of central Africa exhibit substantial increases in upper-tail summer temperatures despite comparatively weak nonlinear interactions, suggesting that other mechanisms, including aerosol forcing, vegetation dynamics, topography, and land-ocean contrasts, may dominate heat amplification in these regions (*61*, *62*). Second, although the identified interaction preferentially amplifies the most extreme heat waves (Fig. 1 and fig. S1), other land-atmosphere processes likely contribute throughout the full heat-wave life cycle, particularly during initiation and decay, including convection, precipitation recycling, cloud feedbacks, and boundary-layer processes (*63*–*65*) (text S1 and figs. S26 and S27). Third, although the LFMIP-pdLC experiment further supports the role of land-atmosphere coupling identified in the ML model (fig. S12), this analysis is based on a single model, and the generalizability of this result across climate models remains to be assessed. Last, our main framework diagnoses contemporaneous interactions and does not explicitly resolve temporal dependencies among circulation, soil moisture, and temperature (*30*). Nevertheless, our additional lagged analysis over CEU shows that the threshold-dependent interaction remains evident across all three prediction directions with a 3-day lag (fig. S5), providing further support for mutual, temporally ordered interactions among the three components. These results strengthen the interpretation of the diagnosed multivariate interaction as a circulation–soil moisture–temperature feedback, while the regional scope of the lagged analysis warrants caution in extending this temporal interpretation globally. Incorporating additional physical drivers together with temporal interactions and process-based model experiments at a global scale represents an important direction for future research.

Our findings suggest that future record-shattering heat waves cannot be understood solely from progressively warmer mean climates or pairwise land-atmosphere feedbacks but can emerge from threshold-dependent multivariate interactions activated beyond critical environmental states. Methodologically, our study demonstrates the value of XAI for identifying such nonlinear interactions while also emphasizing that XAI alone cannot establish physical mechanisms or causality and should therefore be interpreted alongside algorithmic, statistical, and independent physical evidence. This integrated framework may be further extended to other climate extremes shaped by coupled interactions among the atmosphere, land, ocean, and biosphere. More broadly, our results highlight the importance of adequately representing nonlinear multivariate feedbacks in climate models and accounting for them in heat-risk assessment and early-warning systems to better anticipate extreme heat in a warming climate.

## MATERIALS AND METHODS

### Datasets and data preprocess

ERA5-land and ERA5 reanalysis datasets during 1950–2023 are used (*39*, *40*). From ERA5-land dataset, daily maximum 2-m temperature ($T_{x}$, maximum of 6-hourly data at 00:00, 06:00, 12:00, and 18:00 UTC), daily mean (average of 6-hourly data) of surface soil moisture (SM; 0 to 7 cm), surface latent heat flux (LE), surface sensible heat flux (H), and total precipitation are downloaded with resolution of 1° × 1°. From ERA5 data dataset, daily mean (average of 6-hourly data) of geopotential height at 500 hPa (Z500) is downloaded with resolution of 1° × 1°. All data are reprocessed into 3-day mean data without overlapping (1 year includes 365/3≈121 timesteps) considering the general life span of heat-wave events. To test the robustness, temperature from E-OBS (*45*) is also used for CEU regional analysis.

We use multi–Earth system models (ESMs) from CMIP6 and single model initial condition large ensemble (SMILE) to assess the model performance regarding to nonlinear land-atmosphere interaction for heat waves. We select 12 state-of-the-art CMIP6 ESMs (one ensemble for one model) according to data availability, including EC-Earth3, NorESM2-MM, NorESM2-LM, ACCESS-CM2, CanESM5, IPSL-CM6A-LR, MPI-ESM1-2-HR, MPI-ESM1-2-LR, UKESM1-0-LL, INM-CM4-8, INM-CM5-0, and MIROC6. We use 40 ensembles of CESM2-LE2 (*55*, *56*) as the representation for SMILE. Consistent with ERA5 analysis, we use daily maximum 2-m temperature, surface soil moisture (0 to 10 cm), and geopotential height at 500 hPa from the historical (1950–2014) and SSP3–7.0 (a medium to high reference scenario, 2015–2023) simulations. All data are regridded into 1° × 1° and reprocessed into 3-day mean data without overlapping.

To focus on the local physical mechanism in a synoptic time scale, we use the anomaly of data, which refers to data without seasonal cycles and the interannual trends. To remove the seasonal cycle for each grid (or for regional-mean data), the climatology of the corresponding calendar timestep with a 15-day moving window (one window includes five 3-day-mean data) is subtracted. To remove the overall global warming trend for each grid (or for regional-mean data), the 5-year-moving mean (from 2 years before to 2 years after) of the corresponding calendar timestep with a 15-day moving window is subtracted.

### Definition of summer heat-wave event and heat-wave period

To provide the ML model with as large amount of data sample as possible, we use all summer season data during 1950–2023 to train the ML model. However, we only focus on summer heat-wave period (as defined in the following) during 1979–2023 to investigate the heat-wave mechanisms, considering the potentially different physical processes during relatively hot and cold spells and better data quality in the satellite era (after 1979). The summer season is defined as June, July, and August for the temperate region in the Northern Hemisphere (23.5°N to 90°N, excluding Greenland); December, January, and February for temperate region in the Southern Hemisphere (23.5°S to 60°S, excluding Antarctica); and the whole 12 months for the tropics (23.5°N to 23.5°S).

For each grid (or for regional-mean data), heat-wave events are defined as when observed $T_{x}$ anomalies (without seasonal cycle, without warming trend) exceeding the 90th percentile of summer seasons during 1979–2023 for at least 6 days, e.g., the red vertical span in Fig. 2B. Heat-wave periods are defined as the duration from the initiation to termination of the heat waves, e.g., the yellow vertical span in Fig. 2B. The initiation and termination of a heat-wave event are defined when temperature anomalies first exceed and drop below 0, respectively. If initiation (or termination) does not occur within five timesteps (i.e., equal to 15 days) before (or after) the heat-wave peak, then we take the fifth timestep before (or after) the heat-wave peak as the initiation (termination) to keep each heat-wave period no longer than 1 month. Including the whole heat-wave period when analyzing the nonlinearity of the three contributors enables us to investigate the complete evolution process of the heat-wave events.

### Explainable machine learning

We use the 3-day mean data in summer seasons during 1950–2023 to train ML models to guarantee the sufficiency of data amount (74 years×31 timesteps per year=2294 timesteps for temperate region and 74 years×121 timesteps per year=8954 for tropic region). We also provide results with only data during 1979–2023 used to train ML model in supplementary information (fig. S28), which qualitatively confirms the main finding. For each grid (or for regional-mean data), we train the GBR model (python package: sklearn.ensemble.GradientBoostingRegressor) to predict $T_{x}$ with Z500 and SM as inputs. We use Bayesian searcher (python package: skopt.BayesSearchCV) to optimize the hyperparameters. Both input and target variables are deseasonalized and detrended. Following a previous study (*66*), for each grid (or for regional-mean data), we implement repeated fivefold cross-validation 100 times for model training, evaluation, and interpretation, with data randomly shuffled for each run (in total, 5 folds×100 times=500 runs). Fivefold cross-validation means the dataset is split into five equal-sized (or as close to equal as possible) subsets. The model is trained on four of these subsets (training set) but only validated and interpreted with the remaining one subset (testing set). In this way, every individual data would be incorporated into 400 various training sets and be used as the validation set 100 times, which therefore mitigates the overfitting, randomness, and potential bias caused by model baseline.

We use the SHAP approach, which is grounded in the Shapley value from the cooperative game theory (*41*), to interpret the trained GBR (or XGboost) models. The Shapley value quantifies the marginal contribution of an individual “player” to the total gain of a game, as well as the joint contribution of a pair of “players.” The explainable AI framework (*42*) provides a unified and computationally efficient way to estimate these marginal contributions from a trained ML model

$$\phi_{i}=\sum_{S\subseteq M\backslash \left\{i\right\}}\frac{|S|!\left(M-|S|-1\right)!}{M!}\left[f_{x}\left(S\cup \left\{i\right\}\right)-f_{x}\left(S\right)\right]$$ (1)

$$\begin{array}{cc} \phi_{i,j}= & \sum_{S\subseteq M\backslash \left\{i,j\right\}}\frac{|S|!\left(M-|S|-2\right)!}{2\left(M-1\right)!}\\ & \left[f_{x}\left(S\cup \left\{i,j\right\}\right)-f_{x}\left(S\cup \left\{i\right\}\right)-f_{x}\left(S\cup \left\{j\right\}\right)+f_{x}\left(S\right)\right] \end{array}$$ (2)

where $f_{x}$ denotes the trained GBR (or XGBoost) models, M is the full set of M predictors, S is the subset of M, i and j are individual predictors (i≠j), $\phi_{i}$ is the whole contribution of predictor i (i.e., Shapley value), and $\phi_{i,j}$ is the interactive contribution of predictor i and j (i.e., Shapley interaction value).

In our case, we consider two players, i.e., Z500 and SM, and their “total gains” corresponds to the predicted $T_{x}$ ($T_{x,\text{pred}}$) by GBR (or XGboost) models. Therefore, M={Z500,SM} and *M* = 2, and S, in total, includes four possible subsets: {Z500, SM}, {Z500}, {SM}, and ∅ [no predictors, where $f_{x}\left(\emptyset \right)$ equals the expected value of the model’s predictions]. Our target variable is temperature anomaly ($dT_{x}$); hence, $f_{x}\left(\emptyset \right)=\bar{dT_{x}}\approx 0$.

If i=Z500, then M\{i}={{SM},∅}, and Eq. 1 can be written as follows (Eq. 3)

$$\phi_{Z500}=\frac{1}{2}\left[f_{x}\left(Z500,\text{SM}\right)-f_{x}\left(\text{SM}\right)+f_{x}\left(Z500\right)-f_{x}\left(\emptyset \right)\right]$$ (3)

where the SHAP value, $\phi_{Z500}$, represents the total contribution of Z500, consisting of the independent contribution ($C_{Z500}$) plus 50% of the interactive contribution ($C_{\text{interact}}$), because the SHAP framework distributes the interactive contribution equally among the involved predictors.

Similarly, if i=SM, then M\{i}={{Z500},∅}, and Eq. 1 can be written as follows (Eq. 4)

$$\phi_{\text{SM}}=\frac{1}{2}\left[f_{x}\left(Z500,\text{SM}\right)-f_{x}\left(Z500\right)+f_{x}\left(\text{SM}\right)-f_{x}\left(\emptyset \right)\right]$$ (4)

where the SHAP value, $\phi_{\text{SM}},$ represents the total contribution of SM, consisting of the independent contribution ($C_{\text{SM}}$) plus 50% of the interactive contribution ($C_{\text{interact}}$).

If i=Z500 and j=SM, then M\{i,j}=∅, and Eq. 2 can be written as follows (Eq. 5), which explicitly define the interactive term

$$\phi_{Z500,\text{SM}}=\frac{1}{2}\left[f_{x}\left(Z500,\text{SM}\right)-f_{x}\left(Z500\right)-f_{x}\left(\text{SM}\right)+f_{x}\left(\emptyset \right)\right]$$ (5)

Similarly, if i=SM and j=Z500, then Eq. 2 can be written as follows (Eq. 6)

$$\phi_{\text{SM},Z500}=\frac{1}{2}\left[f_{x}\left(Z500,\text{SM}\right)-f_{x}\left(\text{SM}\right)-f_{x}\left(Z500\right)+f_{x}\left(\emptyset \right)\right]$$ (6)

This leads to the quantification of $C_{Z500}$, $C_{\text{SM}}$, and $C_{\text{interact}}$ as follows (Eqs. 7 to 9 and, therefore, Eq. 10)

$$C_{Z500}=\phi_{Z500}-\phi_{Z500,\text{SM}}=f_{x}\left(Z500\right)-f_{x}\left(\emptyset \right)$$ (7)

$$C_{\text{SM}}=\phi_{\text{SM}}-\phi_{\text{SM},Z500}=f_{x}\left(\text{SM}\right)-f_{x}\left(\emptyset \right)$$ (8)

$$\begin{array}{c} C_{\text{interact}}=\phi_{Z500,\text{SM}}+\phi_{\text{SM},Z500}=f_{x}\left(Z500,\text{SM}\right)\\ -f_{x}\left(Z500\right)-f_{x}\left(\text{SM}\right)+f_{x}\left(\emptyset \right) \end{array}$$ (9)

$$C_{Z500}+C_{\text{SM}}+C_{\text{interact}}=f_{x}\left(Z500,\text{SM}\right)-f_{x}\left(\emptyset \right)$$ (10)

As we mentioned above, $f_{x}\left(\emptyset \right)\approx 0$, we therefore obtain $C_{Z500}+C_{\text{SM}}+C_{\text{interact}}=f_{x}\left(Z500,\text{SM}\right)$, i.e., $C_{Z500}+C_{\text{SM}}+C_{\text{interact}}$ is always equal to the predicted $T_{x}$ anomaly.

Using the SHAP implementation in Python (package shap.Explainer, https://github.com/shap/shap), we decompose the $T_{x,\text{pred}}$ of each timestep into a baseline, the direct contribution of Z500 ($C_{Z500}$), direct contribution of SM ($C_{\text{SM}}$), and the interaction contribution of the two drivers ($C_{\text{Interact}}$). Here, “independent” refers to contributions evaluated by conditioning on a single predictor at a time within the SHAP framework and does not imply statistical orthogonality or zero correlation between predictors. Note that the SHAP values are calculated for only the testing set in the fivefold cross-validation, and we report the mean of the 100 repeated runs for better statistical stability.

### Quantification of nonlinearity

To diagnose nonlinear relationship between temperature and the SHAP-derived local contributions ($C_{Z500},C_{\text{SM}},$ and $C_{\text{interact}}$), we apply segmented regression (*67*), a widely used approach for identifying regime-dependent behavior [e.g., the evaporative-regime curve and soil-moisture dynamics; (*47*–*50*)]. We regress the contributions on absolute temperature rather than anomalies to directly link warming-induced increases in threshold exceedance to the amplification of three-way interactions. The segmented model is defined as

$$C=\left\{ \begin{array}{c} m\left(T-T_{c}\right)+C_{c},\;T<T_{c}\\ m_{a}\left(T-T_{c}\right)+C_{c},\;T\geq T_{c} \end{array}\right.$$ (11)

where C refers to the three SHAP-derived contribution, namely, $C_{Z500}$, $C_{\text{SM}}$, and $C_{\text{Interact}}$; T refers to the 3-day mean $T_{x}$; ($T_{c},C_{c}$) marks the critical point that divides the regression into precritical regime (or muted regime) and postcritical regime (or activated regime), with $T_{c}$ referred to as critical temperature; m and $m_{a}$ are the linear-regression slope of the two pieces, with $m_{a}$ referred to as slope of dependence between C and T in activated regime. We use $T_{c}$ and $m_{a}$ to characterize the features of the segmented regression curve.

To assess whether a segmented model provides a superior description relative to a linear model, we evaluate model performance using the AIC (*43*) and the coefficient of determination (*R*^2^ score). AIC is widely used in regression model evaluation because it balances model complexity and goodness of fit by penalizing additional parameters (*44*). Nonlinearity is therefore assessed using the difference in AIC between segmented and linear models

$$∆\text{AIC}={\text{AIC}}_{\text{segmented}}-{\text{AIC}}_{\text{linear}}$$ (12)

Following established practice, a segmented relationship is considered preferable when ∆AIC<−2, reflecting a deliberately conservative criterion. Differences in *R*^2^ score ($∆R^{2}=R_{\text{segmented}}^{2}-R_{\text{linear}}^{2}$) are reported as a supplementary diagnostic.

### Algorithmic robustness across datasets, ML algorithms, and prediction directions

To evaluate the algorithmic robustness of the proposed XAI framework, we examine whether the diagnosed nonlinear interaction depends on the observational dataset, ML algorithm, or prediction direction. Specifically, we repeat the European analysis using the independent E-OBS (*45*) observational dataset, replace the GBR model with XGBoost, and reverse the prediction direction by treating temperature as the predictor and Z500 and SM as target variables, with one timestep between predictors and target variable.

The threshold-activated behavior of the diagnosed Z500-SM interaction is consistently reproduced across both observational datasets (ERA5 and E-OBS) and both ML algorithms (GBR and XGBoost) (fig. S4), demonstrating that the identified nonlinear interaction is insensitive to dataset choice and ML architecture. When the prediction direction is reversed with one timestep between predictors and target variable (using $Z500_{t}$ and ${\mathrm{SM}}_{t}$ to predict $T_{t+1}$, using $Z500_{t}$ and $T_{t}$ to predict ${\mathrm{SM}}_{t+1}$, or using ${\mathrm{SM}}_{t}$ and $T_{t}$ to predict $Z500_{t+1}$), threshold-activated behavior is likewise detected in the temperature-interaction relationships (fig. S5). This result supports the interpretation of reciprocal interactions rather than a purely one-way relationship. Moreover, the persistence of the threshold behavior after reversing the prediction direction reduces concerns that the diagnosed nonlinearity arises solely from mathematical endogeneity or circularity associated with decomposing the target variable.

### Robustness to predictor selection

We examine whether the diagnosed nonlinear interaction depends on the predictor configuration used by the ML model. The main analysis uses two predictors: Z500 and SM. We compare this configuration with three alternative models: (i) a single-predictor model using Z500 alone, (ii) a three-predictor model including surface wind speed (sfcWind), and (iii) a three-predictor model including RH.

The Z500-only model explains 46 ± 14% of the variance in daily maximum temperature across the mid-latitudes, with a 90th-percentile prediction error of 0.94 ± 0.20 K. Including SM substantially improves model performance, increasing the explained variance to 63 ± 14% while reducing the 90th-percentile prediction error to 0.66 ± 0.12 K (fig. S6). This improvement indicates that soil moisture provides substantial predictive information beyond large-scale circulation alone. Including either sfcWind or RH results in only marginal improvements in overall model skill (mean *R*^2^ increases to ∼64%). By contrast, both variables reduce predictive skill for the hottest temperature extremes, increasing the 90th-percentile prediction error to ∼0.74 and 0.71 K, respectively (fig. S6). Moreover, SHAP attribution further shows that the direct contributions of sfcWind and RH remain comparatively weak relative to those from Z500 and SM (figs. S7 and S8). In addition, the threshold-activated interaction between Z500 and SM remains essentially unchanged after including either variable (figs. S7 and S8). By contrast, interactions involving sfcWind or RH do not exhibit evident threshold-activated behavior. These additional experiments indicate that Z500 and SM capture the dominant controls of mid-latitude heat extremes, and the diagnosed threshold-activated interaction among Z500, SM, and T remains robust after accounting for additional atmospheric predictors.

### Statistical robustness of nonlinearity assessment

To evaluate whether the diagnosed threshold behavior reflects a robust nonlinear relationship rather than a consequence of sampling variability, statistical model specification, or sample-size differences, we assess the nonlinearity using four complementary statistical approaches: (i) bootstrap resampling, (ii) likelihood-ratio testing (*46*), (iii) segmented slope-difference testing (*47*, *48*), and (iv) Cohen’s *f*^2^ analysis (*49*). These complementary approaches evaluate different aspects of the diagnosed nonlinearity, including robustness to extreme-event sampling, statistical significance, structural changes in regression slopes, and the magnitude of nonlinear improvement.

#### Bootstrap resampling

Bootstrap resampling is used to evaluate whether the diagnosed threshold behavior is driven by a limited number of exceptional heat waves or by clustered events within individual years. We perform annual-replacement bootstrap resampling, in which all heat-wave samples from one calendar year are replaced by randomly sampled heat-wave observations drawn from the remaining years while maintaining the original sample size. This procedure is repeated 100 times for each of the 45 study years, yielding a total of 4500 bootstrap realizations. For each realization, both segmented and linear regressions are refitted and the corresponding ΔAIC is recalculated. A nonlinear interaction is considered robust when ΔAIC < −2 is satisfied in more than 99% of the bootstrap realizations. To evaluate the sensitivity of this criterion, we repeat the analysis using an alternative model-selection threshold (ΔAIC < 0) and additional bootstrap schemes in which 10 or 20% of the original samples are replaced, in each realization, by randomly resampled observations drawn from the remaining data.

#### Likelihood-ratio test

As a complementary hypothesis-testing approach, we compare the segmented and linear regressions using a likelihood-ratio test (*46*), treating the linear regression as the null model and the segmented regression as the alternative model. The test statistic is calculated as

$$\text{LR}=2\left(\text{log}L_{\text{segmented}}-\text{log}L_{\text{linear}}\right)$$ (13)

where $\text{log}L_{\text{segmented}}$ and $\text{log}L_{\text{linear}}$ denote the log-likelihoods of the segmented and linear models, respectively. Under the null hypothesis, the test statistic approximately follows a chi-square distribution, with degrees of freedom given by the difference in the number of fitted parameters between the two models. A significant result (*P* < 0.05) indicates that the segmented model provides a better description of the data.

#### Segmented slope-difference testing

To verify that the identified breakpoint corresponds to a genuine structural transition rather than a change in model complexity alone, we further test whether the two regression slopes identified by the segmented model differ significantly. Statistical significance of the slope difference is evaluated using the standard coefficient inference implemented in the segmented regression framework (*47*, *48*). A significant slope difference (*P* < 0.05) indicates that the relationship between temperature and the response variable changes significantly across the diagnosed breakpoint.

#### Sample-size effect assessment

Last, we quantify the magnitude of nonlinear improvement using Cohen’s *f*^2^ metric (*49*)

$$\text{Cohen’s}\;f^{2}=\frac{R_{\text{segmented}}^{2}-R_{\text{linear}}^{2}}{1-R_{\text{segmented}}^{2}}$$ (14)

which measures the additional variance explained by the segmented regression relative to the linear model. Unlike significance-based statistics, Cohen’s *f*^2^ quantifies effect of size and is therefore substantially less sensitive to sample-size differences, facilitating comparisons among climate regions with different numbers of heat-wave events. Larger values of Cohen’s *f*^2^ indicate a greater improvement of the segmented regression over the linear alternative.

In the spatial analysis, nonlinear interactions are diagnosed only at grid cells where the ML model reproduces local temperature variability with sufficient skill ($R_{\text{ML}}^{2}>0.6$), ensuring reliable SHAP attribution. Depending on the statistical criterion adopted, including alternative ΔAIC thresholds, bootstrap resampling schemes, likelihood-ratio tests, and segmented slope-difference tests, ∼15 to 30% of humid mid-latitude land areas exhibit robust threshold-activated interactions (table S1), while the identified hot spot regions remain largely unchanged (figs. S16 and S17).

### Independent physical diagnostics

To evaluate whether the XAI-diagnosed interaction is physically consistent with established land-atmosphere theory, we compare the diagnosed threshold behavior and attribution results with three independent physical diagnostics. First, we examine whether the critical temperature threshold identified by the XAI framework is consistent with independently diagnosed evaporative-regime transitions. Second, we compare the SHAP-based attribution with a physically based decomposition derived from the surface energy balance. Third, we use prescribed land-condition experiments from LFMIP (*52*) to provide an independent process-based assessment.

#### Critical soil-moisture threshold

The critical soil moisture is identified on the basis of the relationship between EF and the SM during the dry-down periods, following established approaches in a previous study (*50*). The EF is the ratio of latent heat flux in the total turbulent flux

$$\text{EF}=\frac{\text{LE}}{\text{LE}+H}$$ (15)

where LE and *H* are surface latent and sensible heat fluxes, respectively.

Following Fu *et al.* (*50*), the critical soil moisture is diagnosed using data from summer dry-down periods, which is retained when soil moisture decreases consecutively after rainfall and terminates when soil moisture begins to increase again. During these dry-down periods, the relationship between EF and SM is characterized by fitting a linear-plateau regression

$$\text{EF}=\left\{ \begin{array}{c} k\left(\text{SM}-{\text{SM}}_{c}\right)+{\text{EF}}_{\text{max}},\;\text{SM}<{\text{SM}}_{c}\\ {\text{EF}}_{\text{max}},\;\text{SM}\geq {\text{SM}}_{c} \end{array}\right.$$ (16)

where ${\text{SM}}_{c}$ denotes the critical soil-moisture threshold separating the water-limited ($\text{SM}<{\text{SM}}_{c}$) and energy-limited ($\text{SM}\geq {\text{SM}}_{c}$) evaporative regimes, ${\text{EF}}_{\text{max}}$ represents the plateau value of EF under energy-limited conditions, and *k* is the slope of the EF-SM relationship in the water-limited regime.

To assess whether the linear-plateau segmented regression provides a superior description of the EF-SM relationship relative to the linear regression, we evaluate model performance using $∆\text{AIC}={\text{AIC}}_{\text{segmented}}-{\text{AIC}}_{\text{linear}}$, consistent with the nonlinearity diagnostics applied elsewhere in this study (Eq. 12). We consider the presence of a critical soil-moisture threshold to be statistically supported only when ∆AIC<−2, indicating that the improvement in fit outweighs the penalty associated with additional model parameters.

Following the detection of the critical soil moisture, we further investigate the evaporative regime transition following previous study (*31*, *32*) based the difference in the potential and actual evaporation (fig. S10). This analysis provides a physical mechanism through which soil-moisture depletion amplifies extreme temperatures and influence the troposphere.

#### Surface energy-balance attribution

To independently evaluate the physical interpretation of the SHAP-based attribution, we compare it with an attribution derived from the surface energy balance (*51*). The surface energy balance is expressed as

$$R_{\text{ld}}-R_{\text{lu}}+R_{s}-\text{LE}-H=Q$$ (17)

where $R_{\text{ld}}$ is the downward flux of longwave radiation, $R_{\text{lu}}$ is the emitted upward flux of longwave radiation, $R_{s}$ is the net absorption of solar radiation, LE is the surface latent heat flux, H is the surface sensible heat flux, and Q is the ground heat flux. Note that the $R_{\text{ld}}$ and $R_{s}$ are downward positive, whereas $R_{\text{lu}}$, LE, and H are upward positive.

By using $\sigma {T_{s}}^{4}$ for $R_{\text{lu}}$ (with σ being the Stefan-Boltzmann constant and $T_{s}$ being the surface temperature), we can express $∆T_{s}$ as

$$∆T_{S}=\left(∆R_{\text{ld}}+∆R_{s}-∆\text{LE}-∆H-∆Q\right)/\left(4\sigma {\bar{T_{s}}}^{3}\right)$$ (18)

In this surface energy-balance framework, the effect of high pressure on temperature is majorly through an increase in solar radiation, which is represented by $C_{\text{Rs}}=∆R_{s}/\left(4\sigma {\bar{T_{s}}}^{3}\right)$. This can be interpreted as the marginal contribution of Z500 in the XAI framework, i.e., $C_{Z500}+\frac{C_{\text{interact}}}{2}$. Similarly, soil moisture primarily affects temperature by decreasing latent heat flux, represented by $C_{\text{LE}}=∆\text{LE}/\left(4\sigma {\bar{T_{s}}}^{3}\right)$, which can be interpreted as the marginal contribution of SM in the XAI framework, i.e., $C_{\text{SM}}+\frac{C_{\text{interact}}}{2}$. Compared with surface energy-balance diagnostics, the SHAP-based decomposition shows consistent relative magnitudes between circulation- and land-surface contributions (fig. S11), with the XAI-based contributions slightly larger than those inferred from the energy-balance analysis. This is because the XAI framework attributes temperature variations exclusively to the two selected predictors (Z500 and SM). Consequently, contributions from other energy-balance terms, such as downward longwave radiation, sensible heat flux, and horizontal heat advection, are implicitly redistributed between the baseline value, Z500, and SM in the XAI decomposition.

#### Prescribed land-condition experiments

As a third independent physical assessment, we analyze the prescribed land-condition experiment (LFMIP-pdLC) from the LFMIP (LS3MIP) (*52*). The LFMIP-pdLC simulations use the same climate model, external forcing, sea surface temperature, and sea ice as the corresponding CMIP6 historical and SSP scenario simulations, except that soil moisture is prescribed to the historical mean seasonal cycle during 1980–2014 rather than evolving prognostically. Consequently, comparison between the fully coupled CMIP6 simulations and LFMIP-pdLC provides an independent process-based assessment of the role of evolving soil moisture in the diagnosed circulation–soil moisture–temperature interaction. The prescribed land-condition experiments are now available for only a limited number of CMIP6 models. Among the available LFMIP-pdLC simulations, CMCC-ESM2 provides all variables required for applying the XAI framework at daily resolution, which is therefore selected here. This comparison based on single model output is intended as an independent physical consistency check of the diagnosed mechanism rather than a multimodel evaluation.

Applying the XAI framework to the LFMIP-pdLC simulations substantially weakens the diagnosed interaction compared with the hist+SSP scenario (fig. S12). Relative to the corresponding fully coupled simulations (hist+SSP585, r1i1f1p1), the interaction contribution (*C*_Interact_) is markedly reduced in the corresponding pdLC experiment (amip-lfmip-pdLC, r1i1f1p2, which corresponds to SSP585). Consistently, the evidence supporting a threshold-dependent relationship is substantially weakened: the magnitude of ΔAIC decreases considerably, and its 99% bootstrap CI overlaps zero, indicating that the segmented model is no longer clearly favored over the linear model when soil-moisture variability is suppressed.

### Association between contributors and the upper-tail width of the temperature distribution

To quantify the relationship between the three contributors and changes in the upper tail of the temperature distribution, we analyze the interannual covariability between the heat wave–period mean contributions and the upper-tail width of summer $T_{x}$. The upper-tail width is defined as the difference between the 99.5th and 75th percentiles of summer $T_{x}$ ($T_{x,99.5\text{th}}-$$T_{x,75\text{th}}$), which characterizes changes in the most extreme temperatures relative to moderate warm conditions. This analysis is performed for CEU and extended to mid-latitude humid regions to assess the robustness and spatial consistency of the association. For each region, both the contributor time series and the upper-tail width of $T_{x}$ are computed using a 5-year moving window to suppress short-term variability and to stabilize upper tail quantiles. The resulting time series are then linearly detrended and normalized before regression analysis. Linear regression is applied to quantify the association between interannual variations in the contributor terms and the upper-tail width of the temperature distribution. Regression uncertainty is characterized using the 99% CI of the fitted relationship.

### Spatial division based on model bias in heat-wave changes

To identify the regional sources of bias in CMIP6 simulations of the $T_{x,99.5\text{th}}$ changes, we divide the mid-latitude domain into nine subregions, as shown in Fig. 5D. For each model, we first select grid points with reliable ML performance ($R_{\text{ML}}^{2}>0.6$). These grid points are then grouped into nine bias categories according to the difference between simulated and observed $T_{x,99.5\text{th}}$ changes, ranging from strong underestimation to strong overestimation (−4.5 to −3.5 K, −3.5 to −2.5 K, −2.5 to −1.5 K, −1.5 to −0.5 K, −0.5 to 0.5 K, 0.5 to 1.5 K, 1.5 to 2.5 K, 2.5 to 3.5 K, and 3.5 to 4.5 K). Each model therefore defines its own nine bias ranges based on its individual bias pattern. For each bias range, we then compute the regional mean responses of the three contributors, defined as the linear slopes between temperature and $C_{Z500}$, $C_{\text{SM}}$, and $C_{\text{interact}}$ ($m_{Z500}$, $m_{\text{SM}}$, and $m_{a,\text{interact}}$) during heat-wave periods. Note that the $m_{a,\text{interact}}$ is the slope between $C_{\text{interact}}$ and temperature when $T>T_{c}$.

### Estimating soil-moisture decline rate

The water balance equation during a dry-down (assuming runoff is small) is expressed as

$$\frac{d\text{SM}}{\mathrm{dt}}=P-\text{ET}$$ (19)

where *P* is precipitation and ET is evapotranspiration, with unit of m^3^ m^−3^ day^−1^ to be consistent with the left hand of equation. During mid-latitude heat wave, *P* is approximately zero due to high pressure and clear sky. Thus, the equation simplifies to

$$\frac{d\text{SM}}{\mathrm{dt}}\frac{1}{\text{SM}}=\frac{-\text{ET}}{\text{SM}}$$ (20)

We then call $E_{\text{ET}}=\text{ET}/\text{SM}$ an evapotranspiration efficiency, which measures how efficiently ecosystem uses soil moisture for evapotranspiration (unit: % day^−1^) and is used to assess the soil-moisture decline rate following the heat-wave initiation. By integrating both sides of Eq. 20, we obtain

$$\int \frac{d\text{SM}}{\text{SM}}=\int -E_{\text{ET}}\mathrm{dt}$$ (21)

From Eq. 21, the solution for temporal changes in soil moisture during a heat wave is

$$\text{SM}={\text{SM}}_{0}e^{-E_{\text{ET}}t}$$ (22)

where ${\text{SM}}_{0}$ is the initial soil moisture at the start of the heat-wave period and *t* is the time after heat-wave initiation. Using Eq. 22, we can estimate $E_{\text{ET}}$ by fitting the temporal changes in soil moisture over time after the heat-wave initiation date using an exponential fit (*68*). In CEU, soil moisture declines ∼15 to 27 days after heat-wave onset (fig. S29). Accordingly, $E_{\text{ET}}$ in Fig. 6 is calculated over a 21-day period following heat-wave initiation, with sensitivity tests using 15- and 27-day windows provided in fig. S29, which confirms the negative relationship between the nonlinearity of the interaction and evaporative efficiency.
