# Supplementary material for: Three-way circulation–soil moisture–temperature feedbacks amplify the most extreme mid-latitudinal heat waves
Source: Sci Adv. 2026 Nov 6;12(45):eaeh9111. doi: 10.1126/sciadv.aeh9111 (PMC13742140; doi:10.1126/sciadv.aeh9111)
Supplement: Supplementary file 1 [file sciadv.aeh9111_sm.pdf]

Science Advances
AAAS

Supplementary Materials for

Three-way circulation–soil moisture–temperature feedbacks amplify the most extreme mid-latitudinal heat waves

Yinglin Tian *et al.*

Corresponding author: Yinglin Tian, yinglint@pik-potsdam.de

Sci. Adv. **12**, eaeh9111 (2026)
DOI: 10.1126/sciadv.aeh9111

This PDF file includes:

Supplementary Text S1
Figs. S1 to S29
Table S1

Supplementary Text

Text. S1.

Analysis of the land-air interaction through the full heatwave evolution in Central Europe. It is worth noting that while the positive Z500–SM–T feedback associated with boundary-layer expansion amplifies the most extreme heatwaves at midlatitudes, other land–atmosphere interactions also play crucial roles throughout the full heatwave life cycle. When soil moisture is wetter than climatology, surface heating is primarily driven by radiative forcing, whereas enhanced evaporation with increasing temperature exerts a cooling effect (Supplementary fig. S26). We refer to this situation as the "*moderate state*" (Supplementary fig. S1), in which the positive Z500–SM–T feedback is correlated with enhanced convection (Supplementary fig. S27), likely reflecting intensified latent heat release due to condensation during precipitation *(63)*. Consequently, suppressed convection can yield a negative Z500–SM–T feedback. As heating continues, persistent solar radiation progressively dries the soil, thereby reducing evaporation and thus enhancing surface heating (Supplementary fig. S26, relating to "*heatwave state*" in Supplementary fig. S1). In this way, soil moisture becomes a modulator of temperature. During this state (SM anomaly<0 and $T_x < T_c$), the Z500–SM–T feedback becomes negatively linked to convection and precipitation (Supplementary fig. S27), as dry soil may lead to stronger convection that partly blocks incoming solar radiation *(64, 65)*. However, under this condition, the underlying processes are likely more complex, as negative Z500–SM–T feedbacks can also emerge when convection is reduced. Eventually, once temperature exceeds the critical threshold ("*extreme heatwave state*" in Supplementary fig. S1), the positive Z500–SM–T feedback becomes fully activated through boundary-layer expansion and reinforced Z500 anomalies, corresponding to predominantly positive $C_{interact}$ values and suppressed convection.

Figures

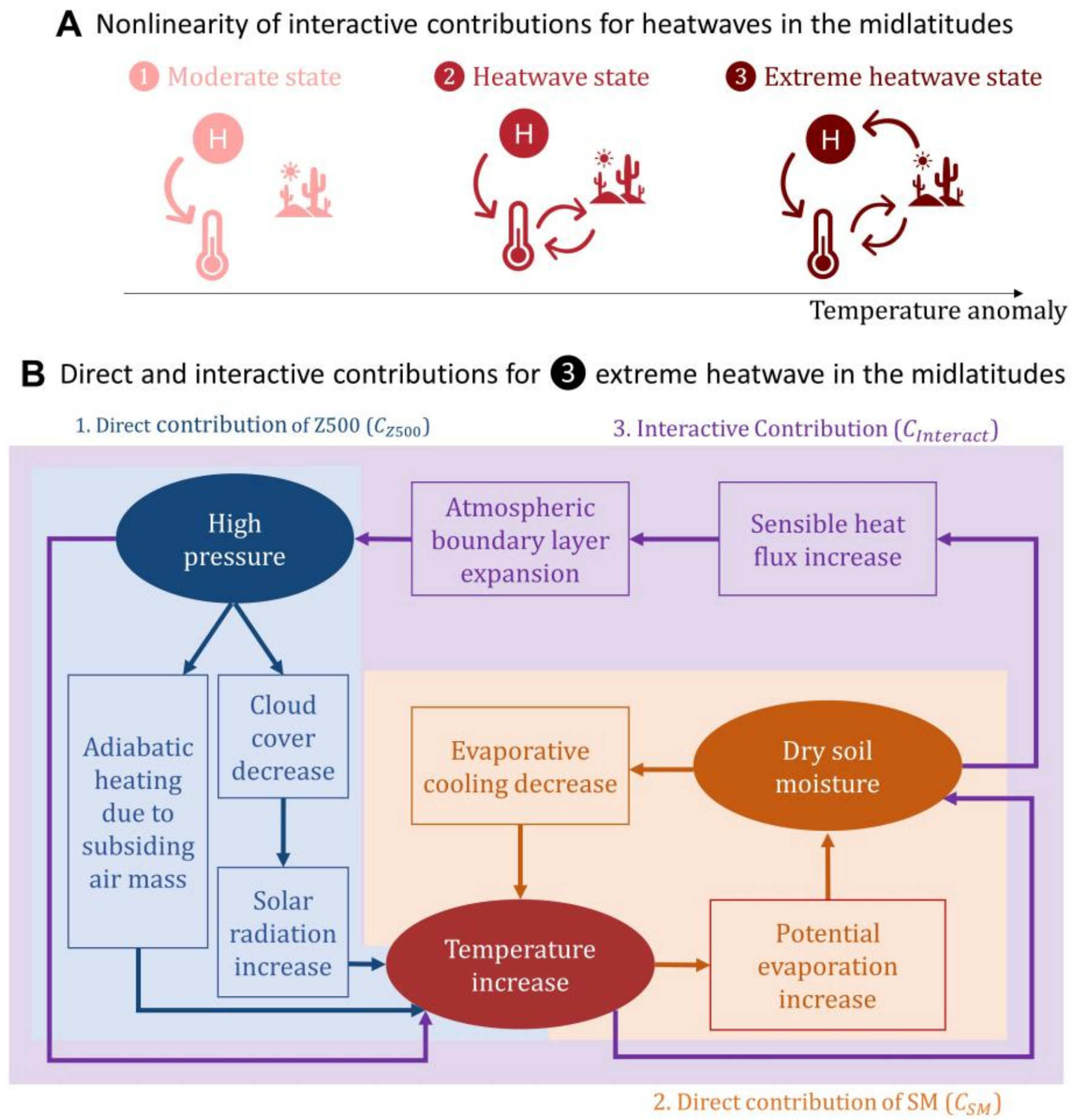

Fig. S1.

Conceptual schematic of the nonlinearity of the Z500–SM–T interaction during the midlatitude heatwaves. (**A**) Three different states during the evolution of heatwaves in the midlatitudes that vary by the temperature anomaly magnitude. Arrows indicate the direction of influence. The third state, i.e., ❸ extreme heatwave state, is investigated in this paper. (**B**) Conceptual schematic of the direct and interactive contributors to the third state. Note that Panel **B** only indicates the mechanisms for ❸ extreme heatwave state in the midlatitudes, while the processes related to other two states, i.e., ❶ moderate state and ❷ heatwave state, are discussed in Supplementary Information Text S1.

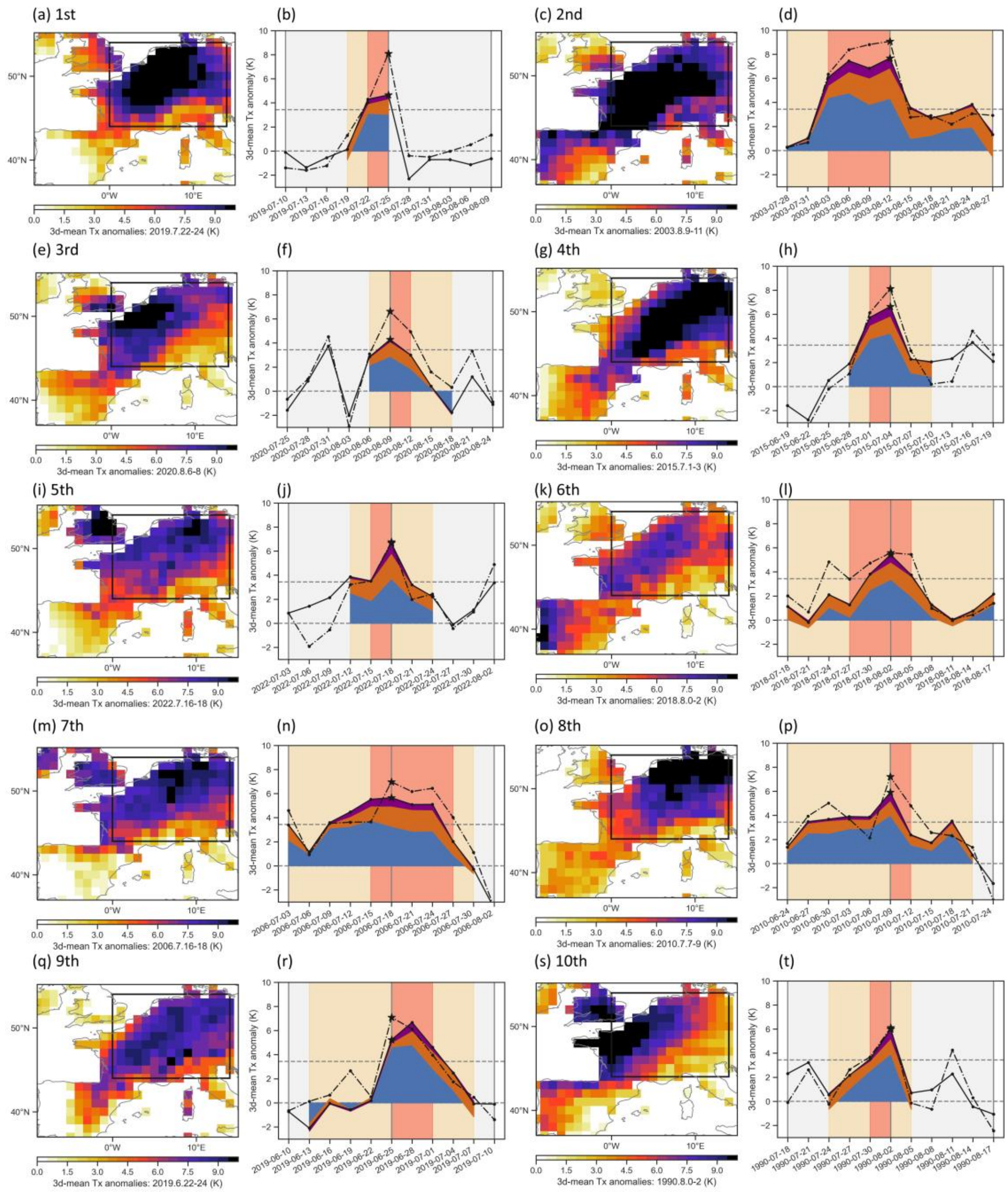

Fig. S2.

Top 10 heatwave events in Central Europe. Similar to Fig. 2A-B but for top 10 heatwave events in Central Europe during summers from 1979 to 2023. **(a, c, e, g, i, k, m, o, q, s)** Spatial distribution of anomalies of the 3-day-mean daily maximum temperature (T_x) at the peaks of the heatwave events, relative to the 1979–2023 calendar climatology. **(b, d, f, h, j, l, n, p, r, t)** XAI model attribution during the heatwave periods, showing observed (dashed) and predicted (solid) T_x anomaly and their decomposition into contributions from mid-tropospheric geopotential height (C_{Z500}, blue), surface soil moisture (C_{SM}, orange), and their interactions ($C_{Interact}$, purple). The heatwave period is defined as days with positive observed T_x anomaly before and after the heatwave peak (yellow horizontal span, see Methods).

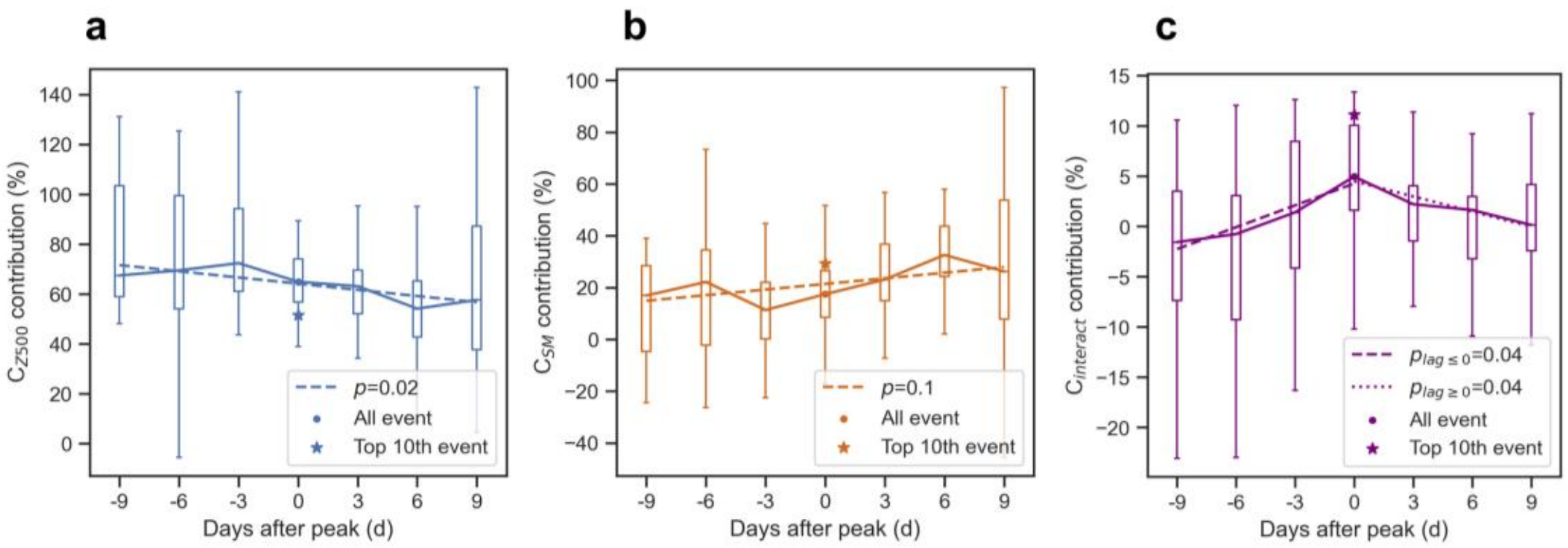

Fig. S3.

Temporal evolution of heatwave contributions in Central Europe. Composite evolution of the three temperature-contribution components before and after heatwave peaks during summers from 1979 to 2023: (**a**) mid-tropospheric geopotential height contribution (C_{Z500}), (**b**) surface soil-moisture contribution (C_{SM}), and (**c**) their interaction term ($C_{Interact}$). Boxplots summarize variability across individual heatwave events at each relative time step: central lines denote medians, boxes indicate interquartile ranges, and whiskers show the 5th–95th percentile. Dashed lines represent linear regressions fitted to the median evolution. Circles and stars mark the mean contribution across all events and across the top 10% most intense heatwave events at the peak, respectively.

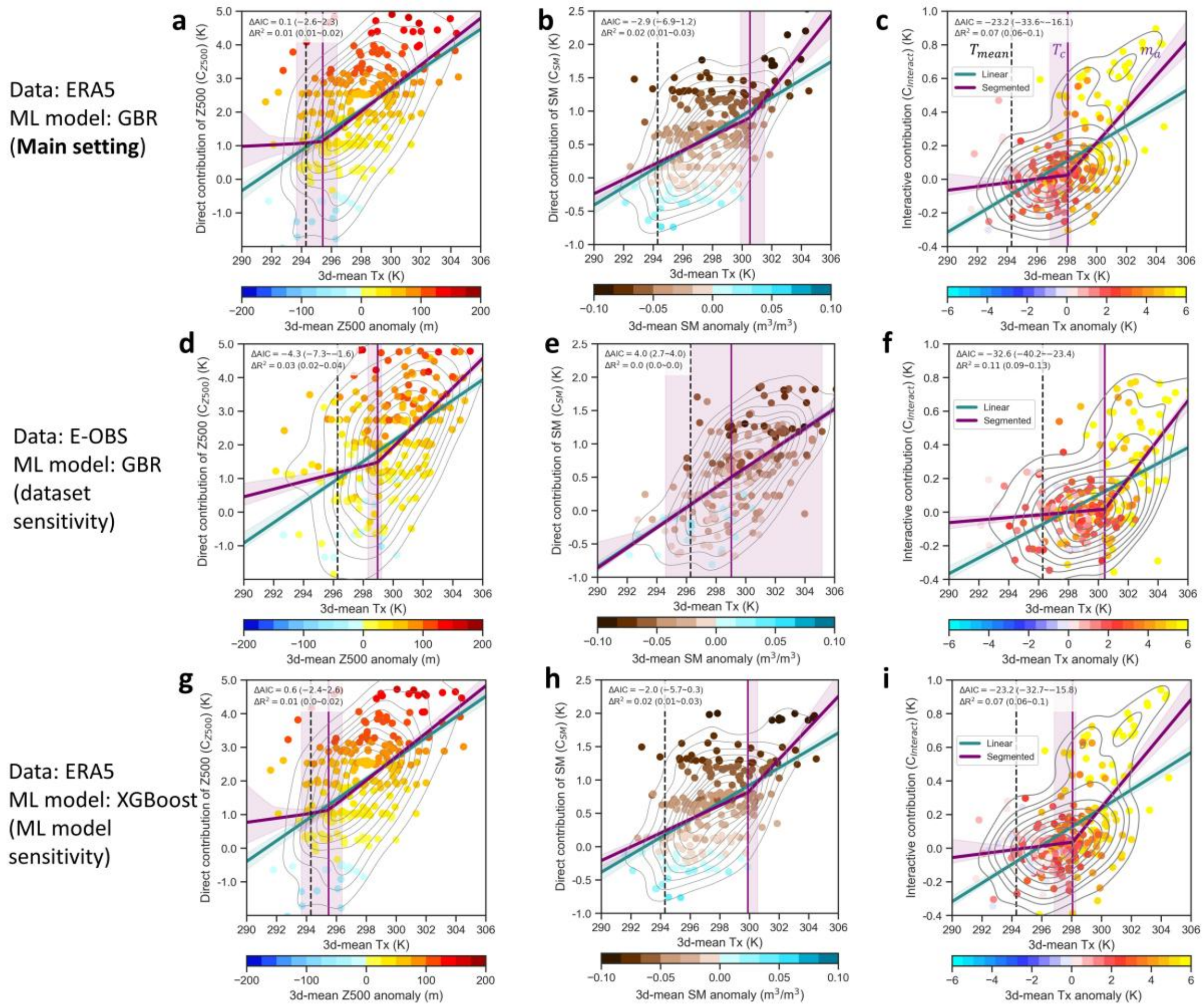

Fig. S4.

Robustness test of datasets and ML model over central Europe. Segmented and linear regression between T_x and the corresponding decompositions, namely, **(a, d, g)** mid-tropospheric geopotential height (C_{Z500}), **(b, e, h)** surface soil moisture (C_{SM}), and **(c, f, i)** their interactions ($C_{Interact}$) during all summer heatwave periods over Central Europe from 1979 to 2023, using (**a, b, c**) ERA5 data+GBR ML model, (**d, e, f**) E-obs data+GBR ML model, (**g, h, i**) ERA5 data+XGBoost ML model. Contours indicate the kernel density estimation (levels from 0.1 to 0.9) and shaded envelopes denote the 99% confidence interval based on annual-replacement bootstrap (see Methods); ΔAIC and ΔR^2 represent performance differences between the segmented and linear regression, with 99% confidence intervals (LCI–UCI); T_{mean} denotes the multi-year mean summer T_x; T_c is the critical temperature separating muted and activated interaction regimes; m_a is the activated-regime slope of the $C_{Interact} - T_x$ relationship.

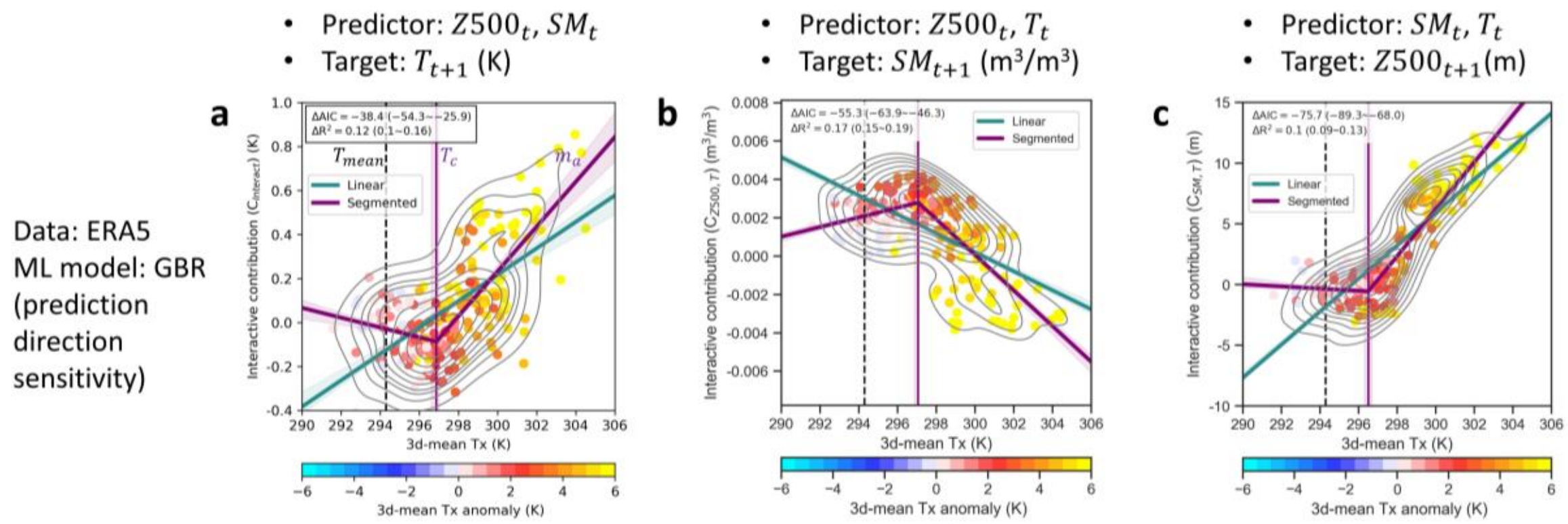

Fig. S5.

Robustness test of XAI prediction direction over central Europe. Segmented and linear regression between T and the interactive contributions during all summer heatwave periods over Central Europe from 1979 to 2023, with ML models using (**a**) $Z500_t$ and SM_t to predict T_{t+1}, (**b**) $Z500_t$ and T_t to predict SM_{t+1}, and (**c**) SM_t and T_t to predict $Z500_{t+1}$. That is, the predictors have 1 lag (3 days) relative to the target variables in ML models. Contours indicate the kernel density estimation (levels from 0.1 to 0.9) and shaded envelopes denote the 99% confidence interval based on annual-replacement bootstrap (see Methods); ΔAIC and ΔR^2 represent performance differences between the segmented and linear regression, with 99% confidence intervals (LCI–UCI); T_{mean} denotes the multi-year mean summer T_x; T_c is the critical temperature separating muted and activated interaction regimes; m_a is the activated-regime slope of the $C_{Interact} - T_x$ relationship.

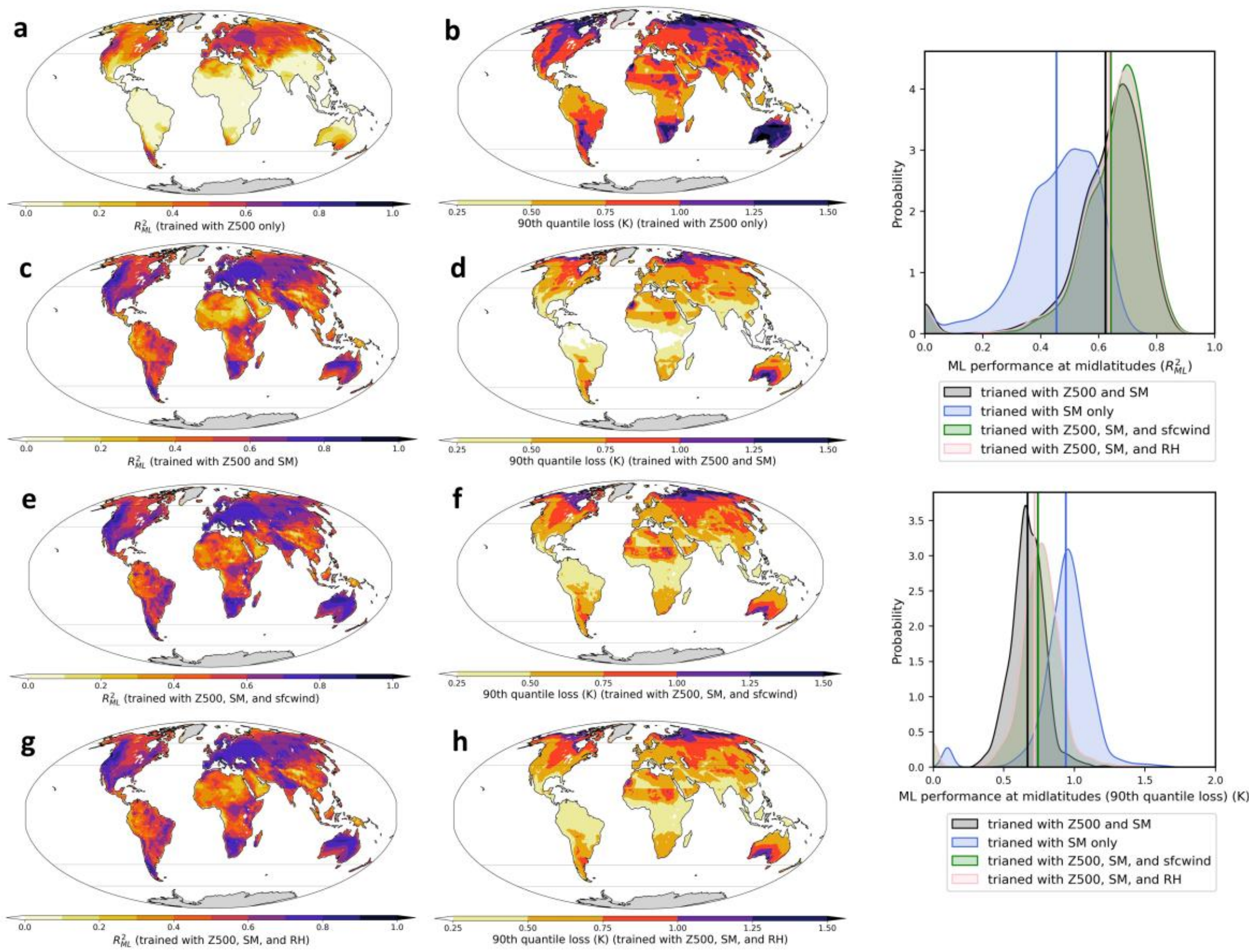

Fig. S6.

Gradient Boosting Regressor (GBR) model performance at the global scale using different predictor sets. **(a, c, e, g)** Spatial distribution of R^2 and **(b, d, f, h)** the 90th-quantile prediction error between GBR-predicted and ERA5 T_x during summer (1979–2023), for models trained with Z500 only **(a, b)**, Z500 + SM **(c, d)**, Z500 + SM + sfcwind **(e, f)**, and Z500 + SM + RH **(g, h)**. Horizontal grey lines indicate the midlatitude band (40°–60°). The Probability distributions at the right side are R^2 and 90th-quantile error at midlatitudes for the different predictor sets.

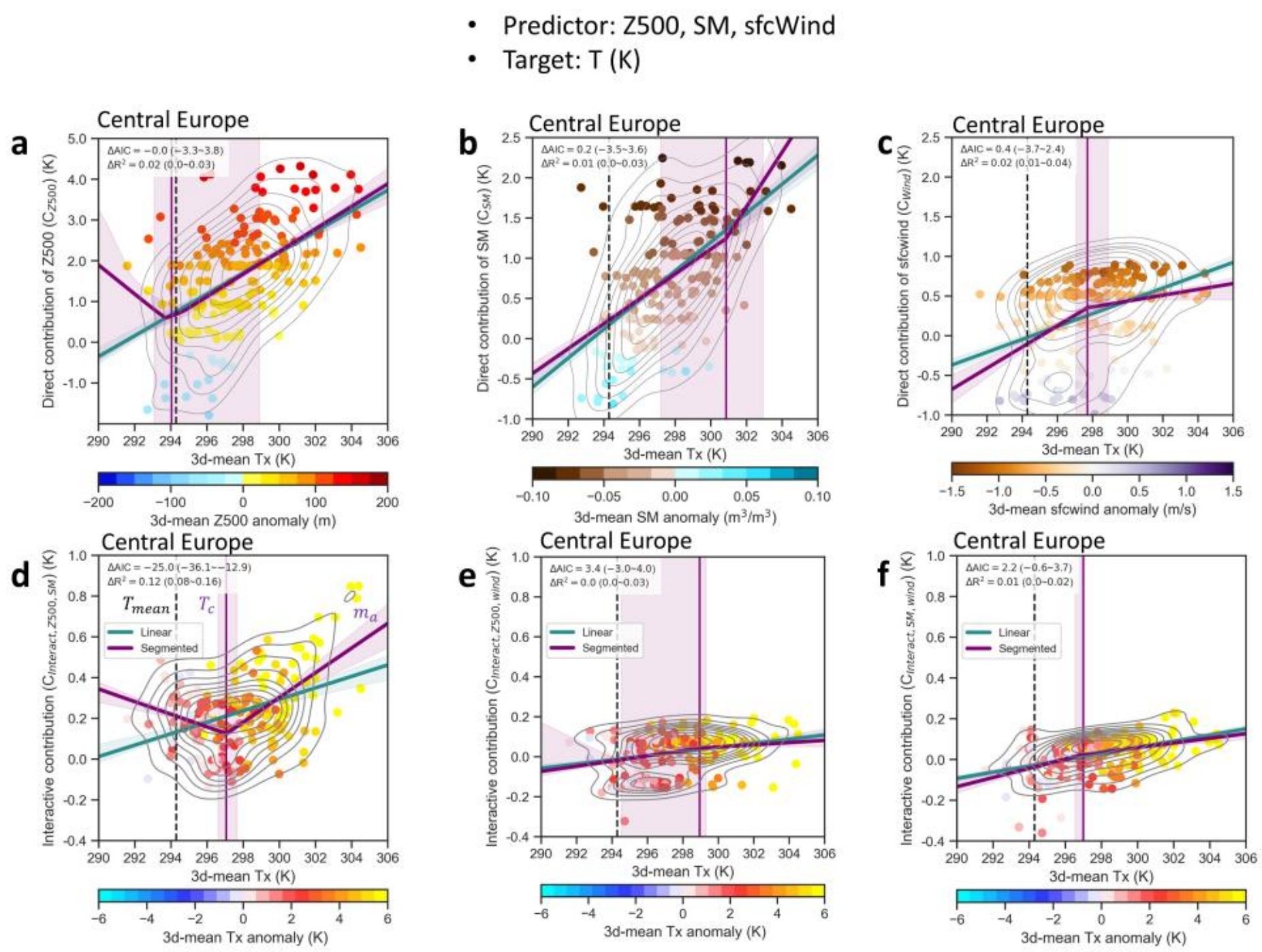

Fig. S7.

Robustness of predictor choice over central Europe and midlatitudes considering wind speed. Regional analysis in Central Europe, with the GBR model trained with Z500, SM, and sfcWind to predict temperature. (**a-c**) Segmented and linear regression between T_x and the SHAP-derived contributions from the GBR machine learning model: direct contribution from Z500 (C_{Z500}; **a**), SM (C_{SM}; **b**), and sfcwind (C_{wind}; **c**), as well as interaction terms, including $C_{interact,Z500-SM}$ (**d**), $C_{interact,SM-wind}$ (**e**), and $C_{interact,SM-wind}$ (**f**), evaluated for all summer heatwave periods over Central Europe (1979–2023). Contours indicate the kernel density estimation (levels from 0.1 to 0.9) and shaded envelopes denote the 99% confidence interval based on annual-replacement bootstrap (see Methods); ΔAIC and ΔR^2 represent performance differences between the segmented and linear regression, with 99% confidence intervals (LCI–UCI); T_{mean} denotes the multi-year mean summer T_x; T_c is the critical temperature separating muted and activated interaction regimes; m_a is the activated-regime slope of the $C_{Interact} - T_x$ relationship.

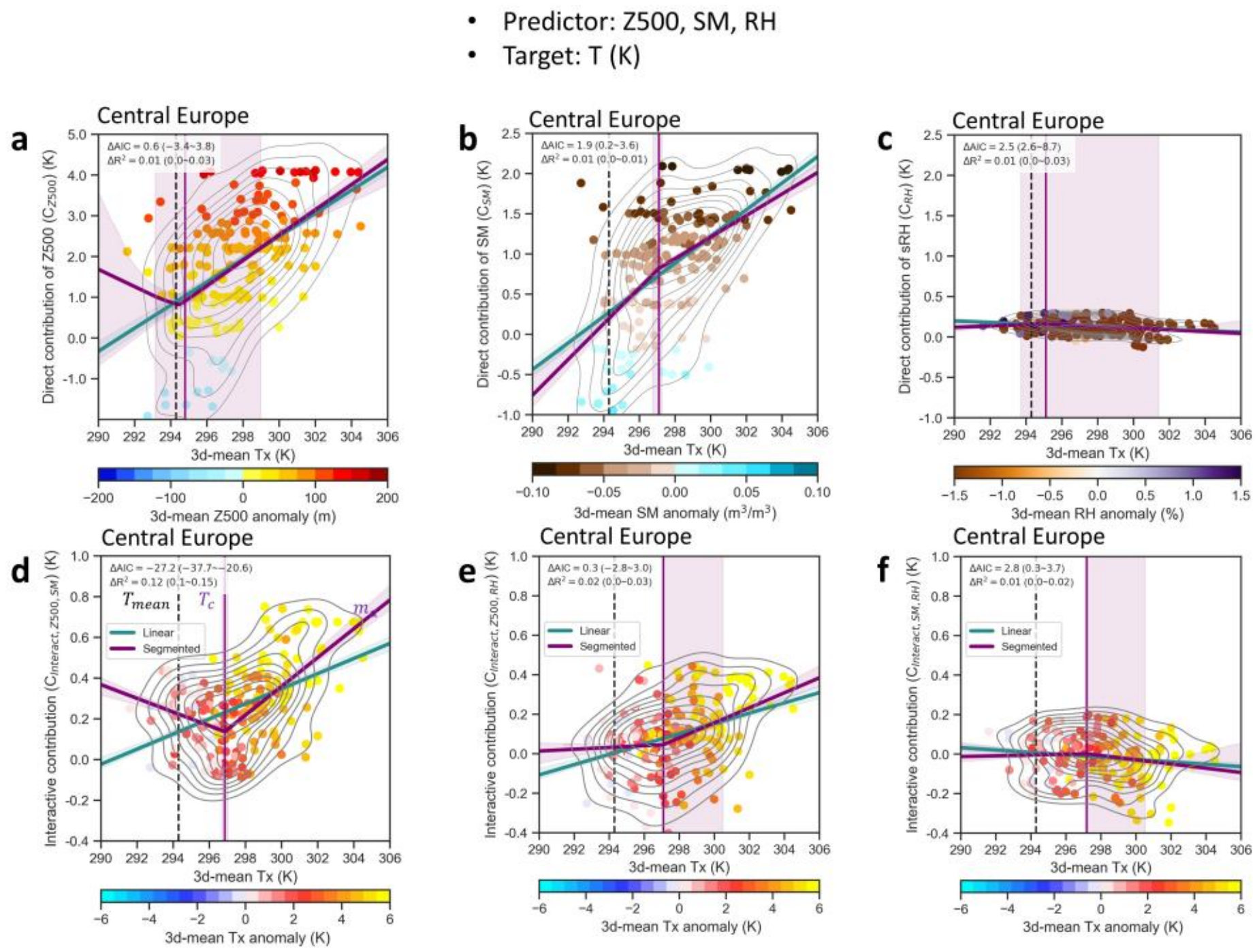

Fig. S8.

Robustness of predictor choice over central Europe and midlatitudes considering relative humidity. Regional analysis in Central Europe, with the GBR model trained with Z500, SM, and RH to predict temperature. (**a-c**) Segmented and linear regression between T_x and the SHAP-derived contributions from the GBR machine learning model: direct contribution from Z500 (C_{Z500}; **a**), SM (C_{SM}; **b**), and RH (C_{RH}; **c**), as well as interaction terms, including $C_{interact,Z500-SM}$ (**d**), $C_{interact,SM-RH}$ (**e**), and $C_{interact,SM-RH}$ (**f**), evaluated for all summer heatwave periods over Central Europe (1979−2023). Contours indicate the kernel density estimation (levels from 0.1 to 0.9) and shaded envelopes denote the 99% confidence interval based on annual-replacement bootstrap (see Methods); ΔAIC and ΔR^2 represent performance differences between the segmented and linear regression, with 99% confidence intervals (LCI–UCI); T_{mean} denotes the multi-year mean summer T_x; T_c is the critical temperature separating muted and activated interaction regimes; m_a is the activated-regime slope of the $C_{Interact} - T_x$ relationship.

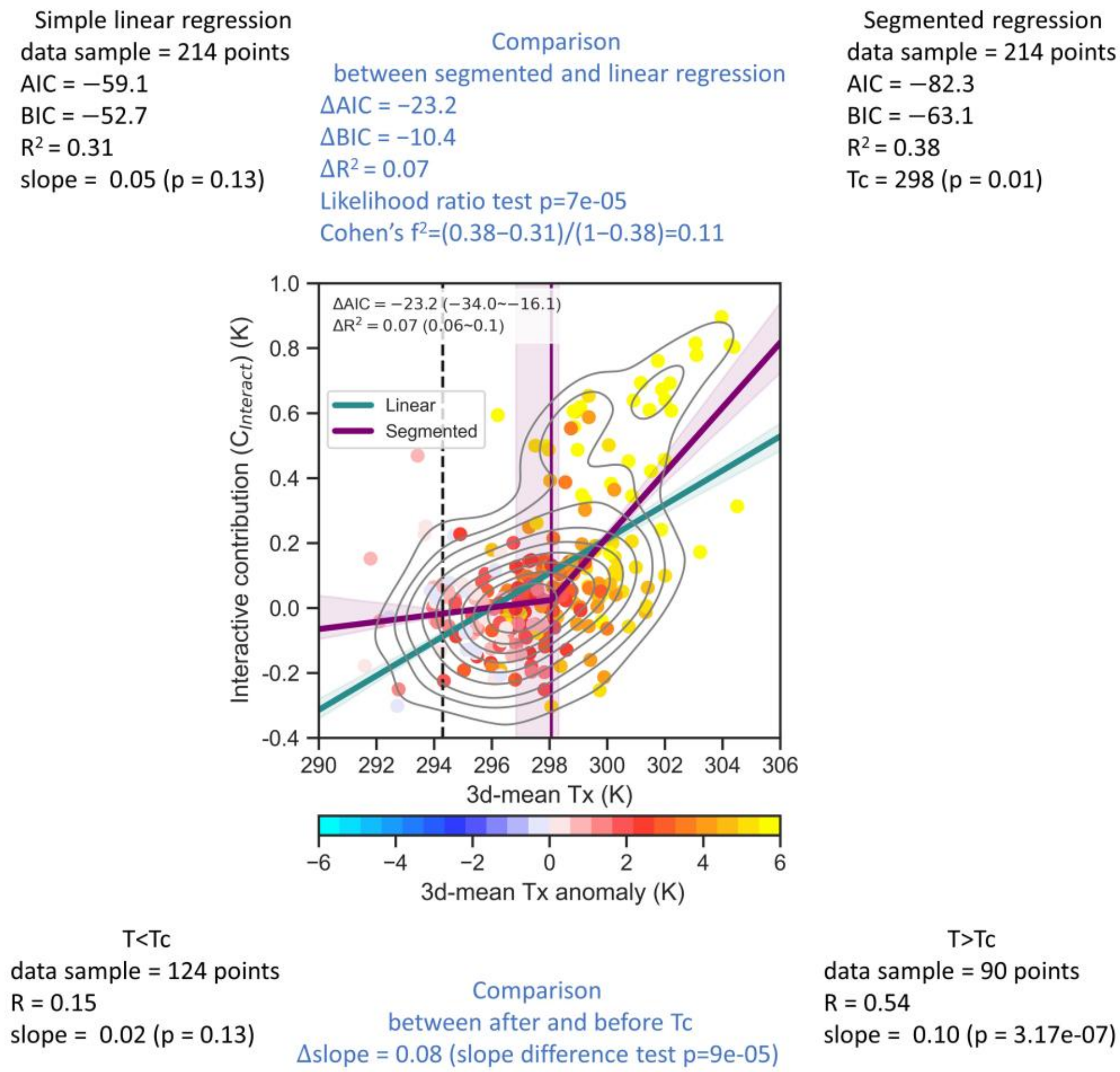

Fig. S9.

Significant tests of the nonlinearity of the three-way interactions in Central Europe based on LRT and segmented slope differences. Segmented and linear regression between T_x and the three-way interactions ($C_{Interact}$) during all summer heatwave periods over Central Europe from 1979 to 2023, using ERA5 data+GBR ML model. Contours indicate the kernel density estimation (levels from 0.1 to 0.9) and shaded envelopes denote the 99% confidence interval based on annual-replacement bootstrap (see Methods); ΔAIC and ΔR^2 represent performance differences between the segmented and linear regression, with 99% confidence intervals (LCI–UCI); T_{mean} denotes the multi-year mean summer T_x; T_c is the critical temperature separating muted and activated interaction regimes; m_a is the activated-regime slope of the $C_{Interact} - T_x$ relationship. The upper text records the performance between the simple linear regression and segmented regression (black text) and their comparison using the difference in AIC, BIC, R^2, the *p*-value from the likelihood ratio test, and Cohen's f^2 value (blue text). The lower text records the parameters of the linear regression before and above the critical temperature (black text) and their slope difference with the significance (blue text).

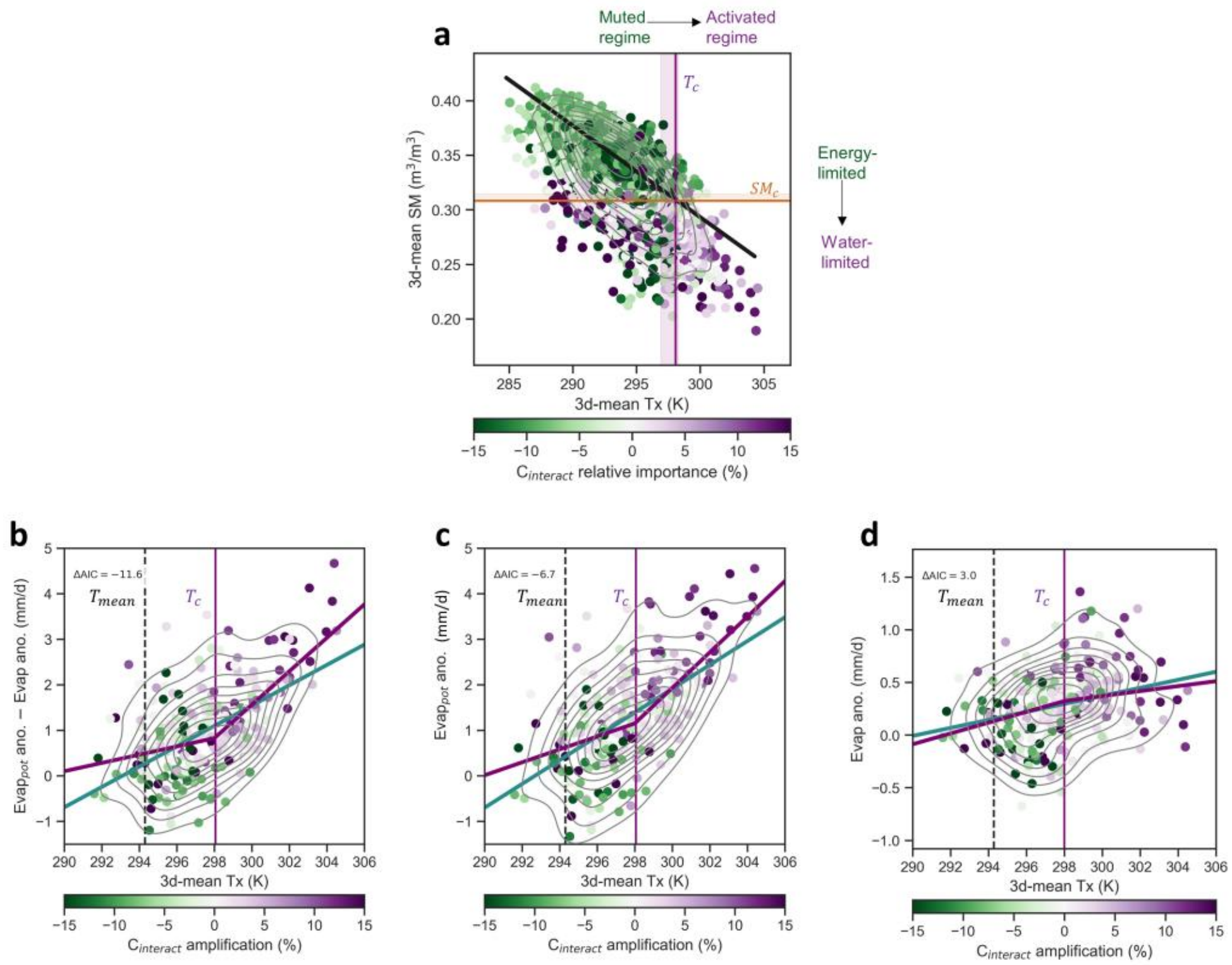

Fig. S10.

Physical interpretation of the nonlinear circulation–soil-moisture–temperature interaction in Central Europe based on critical soil moisture and evaporation diagnostics.

(**a**) Linear regression between the 3-day mean daily maximum temperature (T_x) and surface soil moisture (SM) across all summer days over Central Europe. Contours indicate the kernel density estimation (levels from 0.1 to 0.9), and shaded envelopes indicate the 99% confidence interval for both linear and segmented regressions. T_c denotes the critical temperature marking the transition of land–atmosphere interactions from a muted to an activated regime, while SM_c denotes the critical soil-moisture threshold separating energy- and water-limited evaporation. Notably, T_c and SM_c are identified independently: T_c is diagnosed from the $C_{Interact} - T_x$ relationship during heatwave periods (Fig. 2C), and SM_c is diagnosed from the $SM - EF$ relationship during dry-down periods (Fig. 2D). These independently derived thresholds intersect along the summer-wide $T_x - SM$ regression shown here, indicating that the critical temperature coincides with the critical soil-moisture under typical summer conditions. (**b–d**) Segmented and linear regressions between Central Europe–mean daily maximum temperature anomaly (T_x) and (**b**) the difference between potential and actual evaporation anomaly ($E_{pot} - E$), (**c**) potential evaporation anomaly (E_{pot}), and (**d**) actual evaporation anomaly (E) during all summer heatwave periods from 1979 to 2023. The dashed black vertical line denotes the multi-year mean summer T_x, while the solid purple line indicates the critical temperature threshold (T_c) separating the muted and activated interaction regimes, which is the T_c diagnosed from the $C_{Interact} - T_x$ relationship during heatwave periods (Fig. 2C). Contour indicates the kernel density estimation (levels from 0.1 to 0.9).

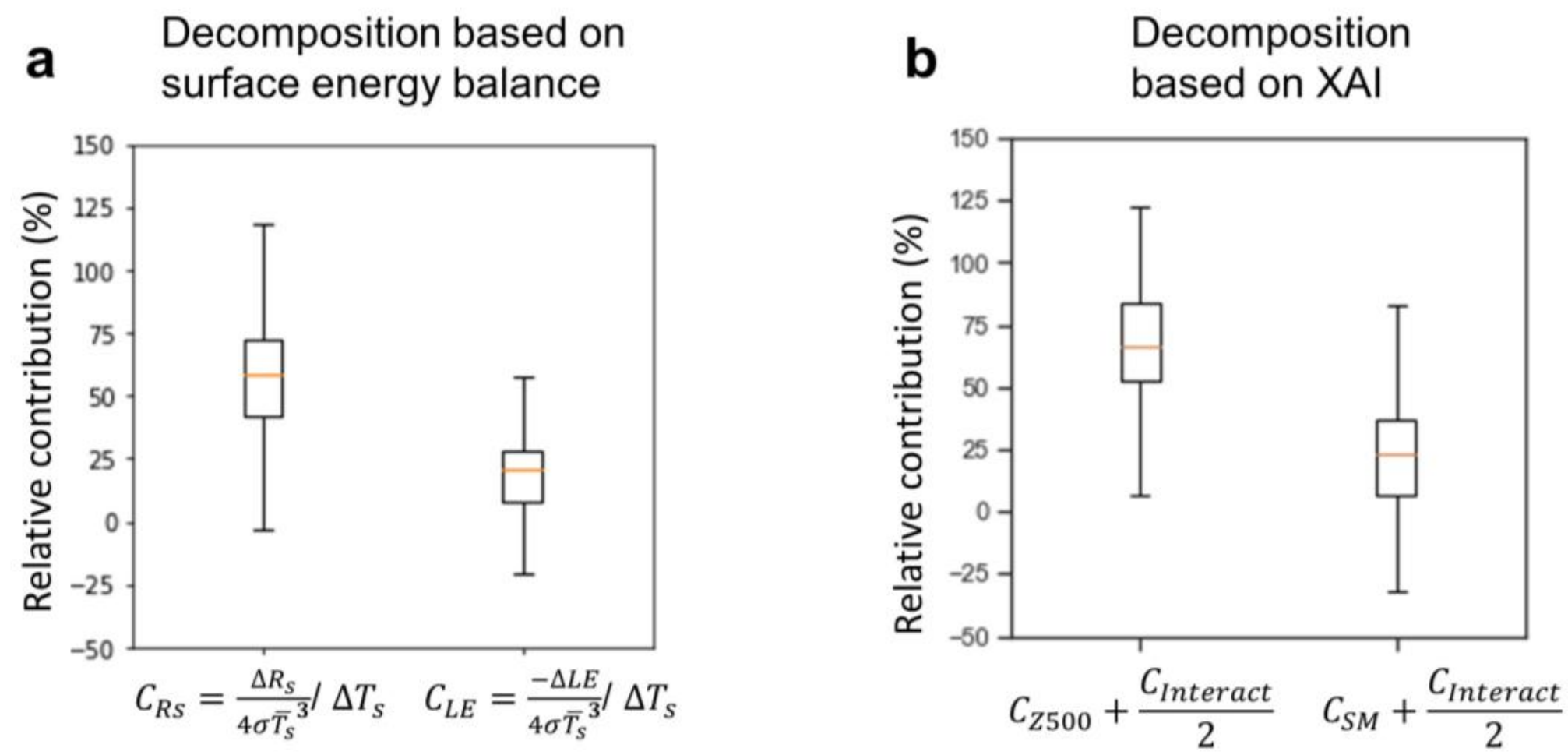

Fig. S11.

Contribution of atmospheric and surface drivers to summer heatwaves in Central Europe. Comparison of contributions estimated from (**a**) surface energy-balance perturbation analysis and (**b**) the explainable artificial intelligence (XAI) framework during summer heatwave days (1979–2023). The XAI-based contributions in panel **b** are slightly larger than those inferred from the energy-balance analysis in panel **a**, because the XAI framework attributes temperature variations exclusively to the two selected predictors (Z500 and soil moisture). Consequently, contributions from other energy-balance terms, such as downward longwave radiation (R_{ld}), sensible heat flux (H), and horizontal heat advection (Q), are implicitly redistributed between Z500 and soil moisture in the XAI decomposition. Boxplots summarize variability across heatwave events: the central line indicates the median, boxes denote the interquartile range, and whiskers represent the 5th–95th percentile.

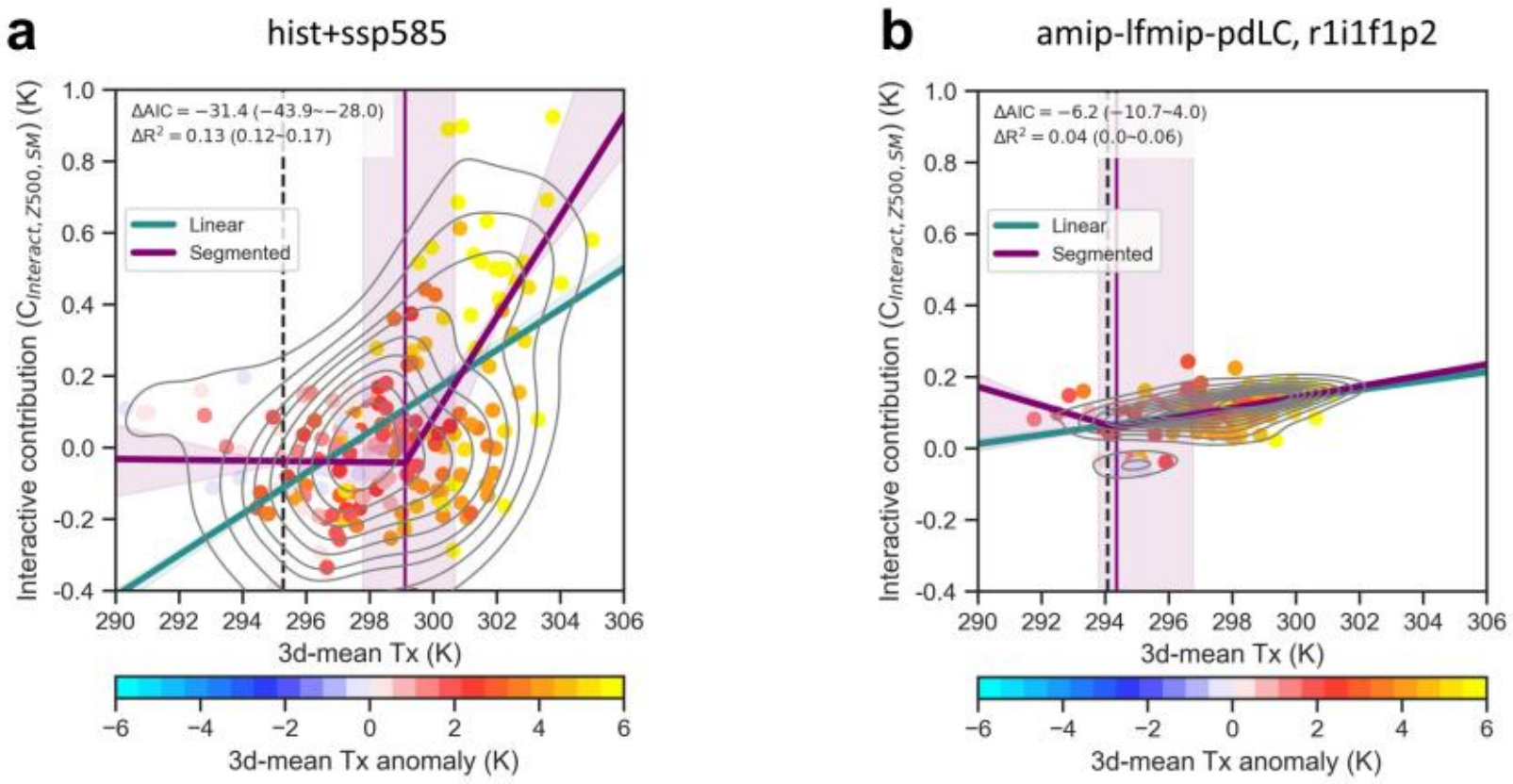

Fig. S12.

Independent process-based assessment of the XAI-diagnosed interaction using the LS3MIP LFMIP-pdLC experiment. Segmented and linear regressions between summer maximum temperature and the interaction contribution for all summer heatwave days (1980–2023) over Central Europe, with the explainable machine-learning model trained separately using output from CMCC-ESM2 from (**a**) the fully coupled CMIP6 historical/SSP585 simulations (ensemble member r1i1p1f1), in which soil moisture evolves prognostically, and (**b**) the LFMIP-pdLC experiment (ensemble member r1i1p1f2, which corresponds to SSP585), in which soil moisture is prescribed to the historical mean seasonal cycle during 1980–2014 following the LS3MIP protocol. Contours indicate the kernel density estimation (levels from 0.1 to 0.9) and shaded envelopes denote the 99% confidence interval based on annual-replacement bootstrap (see Methods); ΔAIC and ΔR^2 represent performance differences between the segmented and linear regression, with 99% confidence intervals (LCI–UCI); T_{mean} denotes the multi-year mean summer T_x; T_c is the critical temperature separating muted and activated interaction regimes; m_a is the activated-regime slope of the $C_{Interact} - T_x$ relationship.

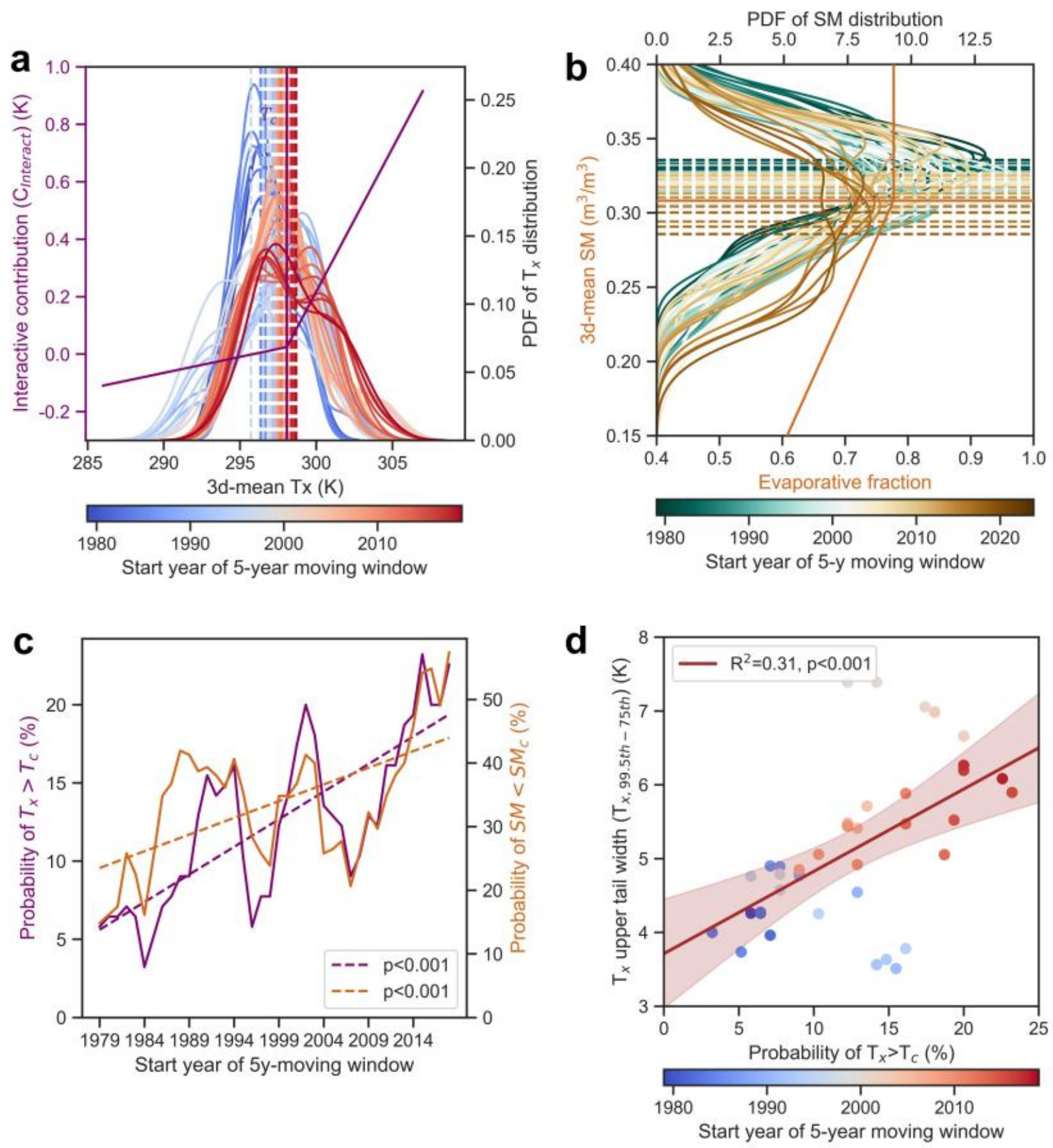

Fig. S13.

Increasing exceedance of the critical temperature enhances land–atmosphere coupling and widens the upper tail of the temperature distribution in Central Europe (1979–2023). (**a**) Temporal evolution of the summer distribution of daily maximum temperature (T_x) computed using a 5-year moving window. (**b**) Corresponding evolution of the summer soil-moisture (SM) distribution using the same 5-year moving window. (**c**) Changes in the summertime probability of the critical threshold crossing, defined as the probabilities of $T_x > T_c$ and $SM < SM_c$, calculated with a 5-year moving window. (**d**) Linear regression between the upper-tail width of the temperature distribution ($T_{x,99.5th} - T_{x,75th}$) and the exceedance probability of the critical temperature threshold (probability of $T_x > T_c$), with both quantities derived from 5-year moving windows.

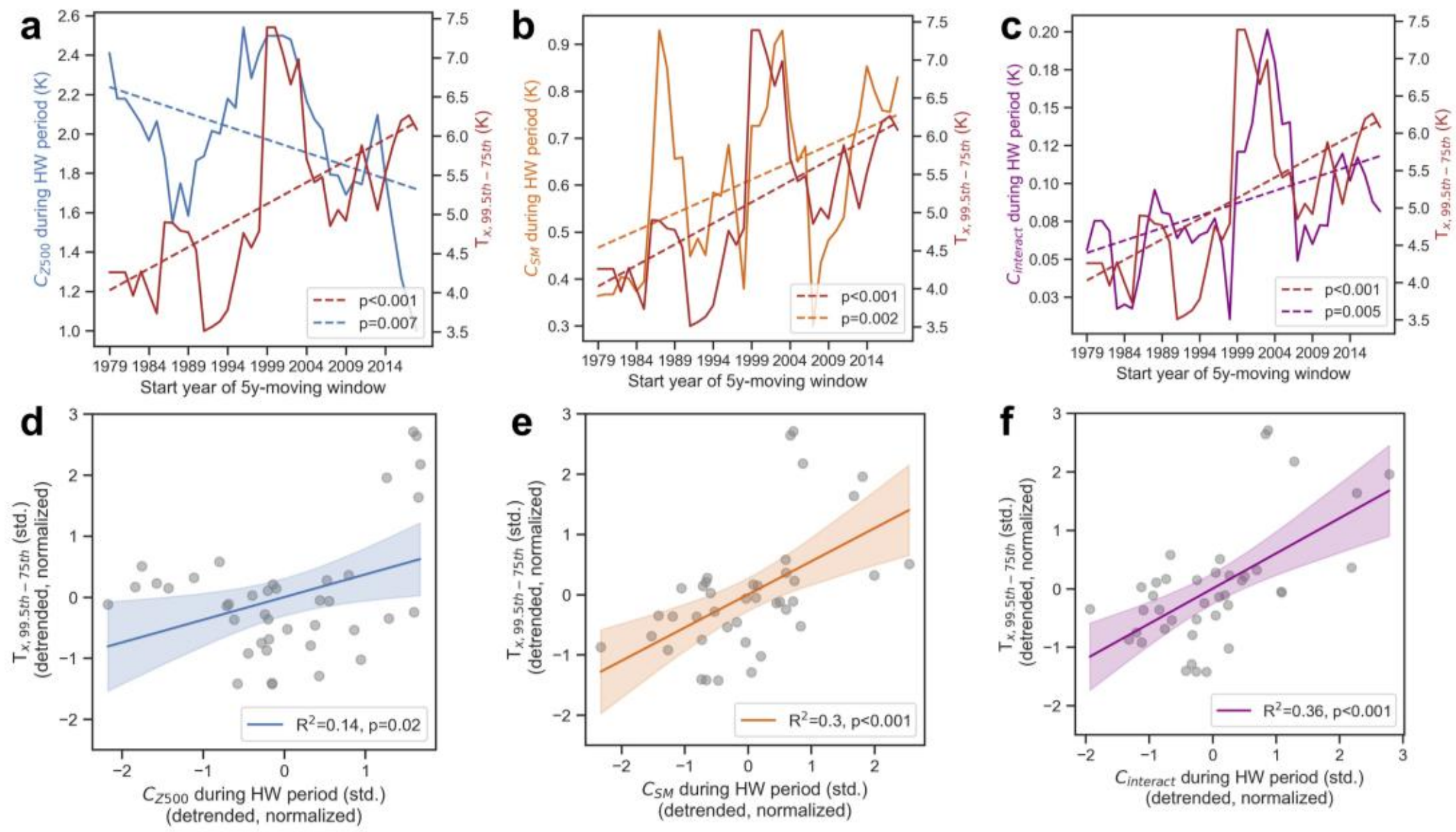

Fig. S14.

Associations between upper-tail temperature variability and circulation, soil moisture, and interaction contributions in Central Europe during summer heatwaves (1979–2023). (**a–c**) Temporal evolution of the 5-year moving-window upper-tail width of daily maximum temperature ($T_{x,99.5th} - T_{x,75th}$) and the corresponding 5-year moving-window mean contributions from (**a**) mid-tropospheric geopotential height (C_{Z500}), (**b**) surface soil moisture (C_{SM}), and (**c**) their interaction ($C_{interact}$). (**d–f**) Linear regressions between the 5-year moving-window upper-tail width of T_x and the corresponding 5-year moving-window mean contributions from (**d**) C_{Z500}, (**e**) C_{SM}, and (**f**) $C_{interact}$ during heatwave periods. In panels (**d, e, f**), all time series are detrended and normalized prior to regression.

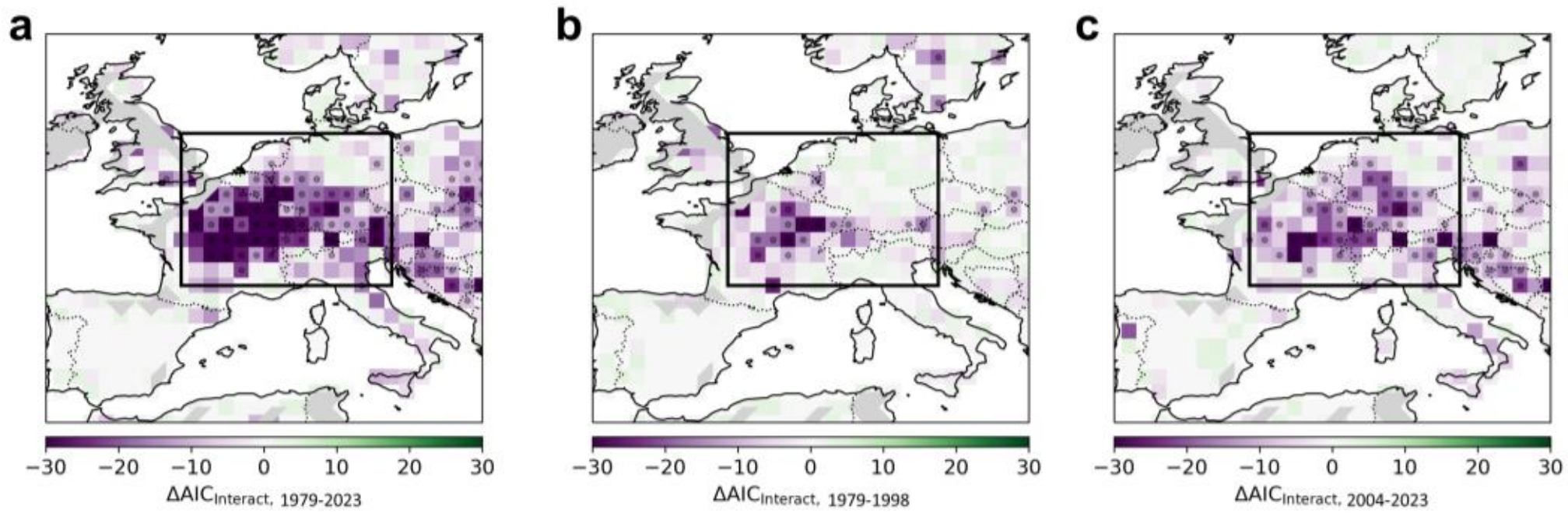

Fig. S15.

Expansion of regions exhibiting nonlinear Z500–SM–T interactions in Central Europe. Spatial distribution of the nonlinearity of the Z500–SM–T interaction, quantified by $\Delta\text{AIC}_{interact}$, during summer heatwave periods over **(a)** 1979–2023, **(b)** 1979–1998, and **(c)** 2004–2023. Black dots indicate grid points exhibiting robust nonlinear behavior, defined by $\Delta\text{AIC}_{interact} < -2$ and confirmed in ≥99% of annual-replacement bootstrap realizations (see Methods). Grey shading denotes regions where the machine-learning model explains less than 60% of local temperature variance ($R^2_{ML} < 0.6$; see Methods). The black rectangle delineates the Central Europe domain.

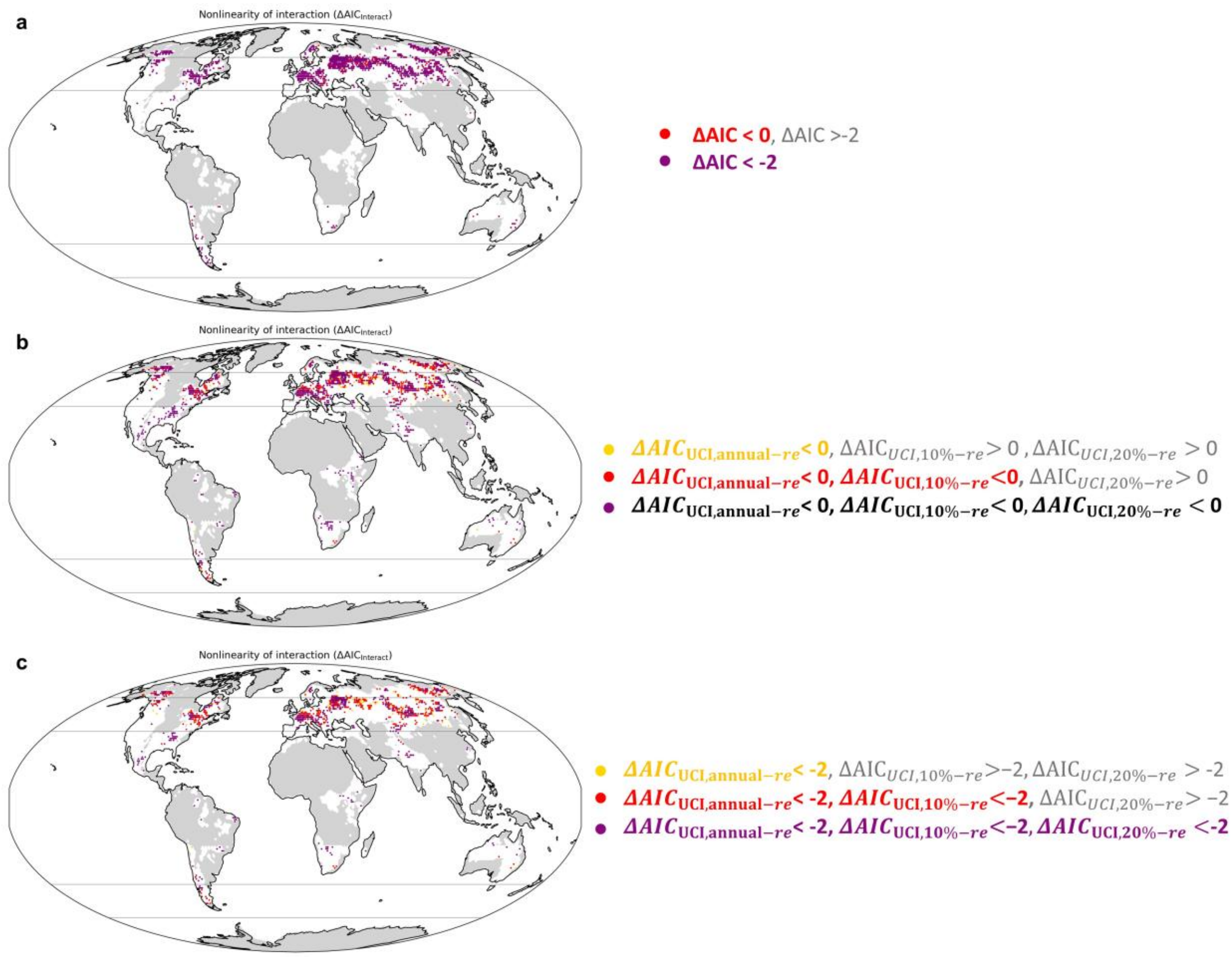

Fig. S16.

Sensitivity of identified nonlinear interaction regions to regression model-selection criteria and bootstrap methods. (a) Grid points exhibiting nonlinear Z500–SM–T interaction under unbootstrapped criteria, with $\Delta AIC < 0$ but $\Delta AIC \geq -2$ (red) and $\Delta AIC < -2$ (purple). **(b)** Grid points exhibiting nonlinear interaction under bootstrapped $\Delta AIC < 0$, confirmed by ≥99% of the annual-replacement bootstrap realizations only (yellow), by both annual- and 10%-replacement bootstraps (red), and by all three bootstrap schemes (annual-, 10%-, and 20%-replacement; purple). **(c)** Same as (b), but for the more stringent criterion $\Delta AIC < -2$. These panels demonstrate that the spatial pattern of nonlinear Z500–SM–T interactions is robust across model-selection thresholds and resampling strategies, while stricter criteria progressively reduce the affected area.

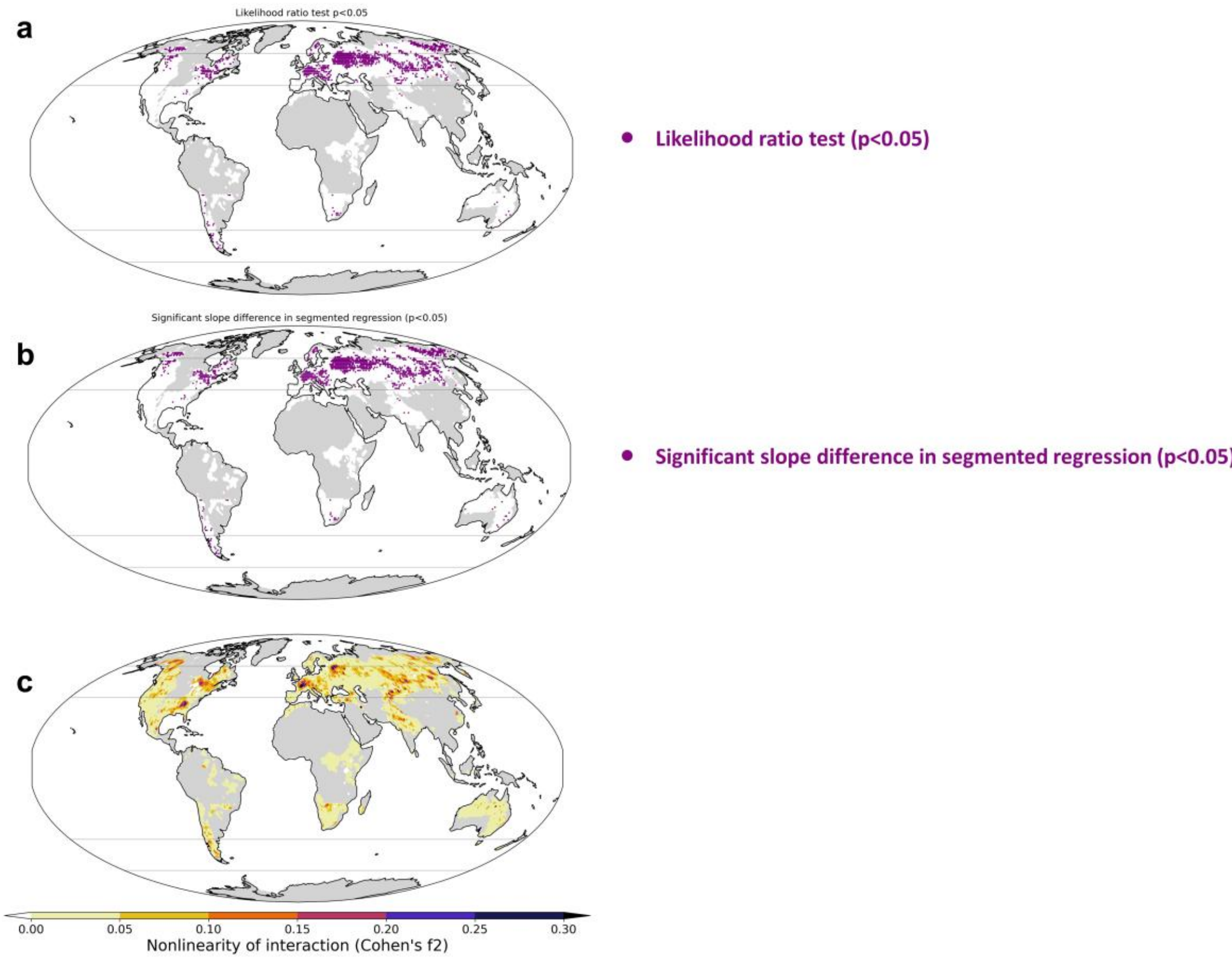

Fig. S17.

Sensitivity of identified nonlinear interaction regions to statistic diganostics. (a) Grid points exhibiting nonlinear Z500–SM–T interaction under likelihood ratio test ($p<0.05$, purple). **(b)** Grid points exhibiting nonlinear Z500–SM–T interaction under segmented slope difference significance test ($p<0.05$, purple). (**c**) Distribution of Cohen's f^2 value for the nonlinear diagnostic of the Z500–SM–T interaction. Fig. S16 and S17 demonstrate that the spatial pattern of nonlinear Z500–SM–T interactions is robust across statistic diganostics.

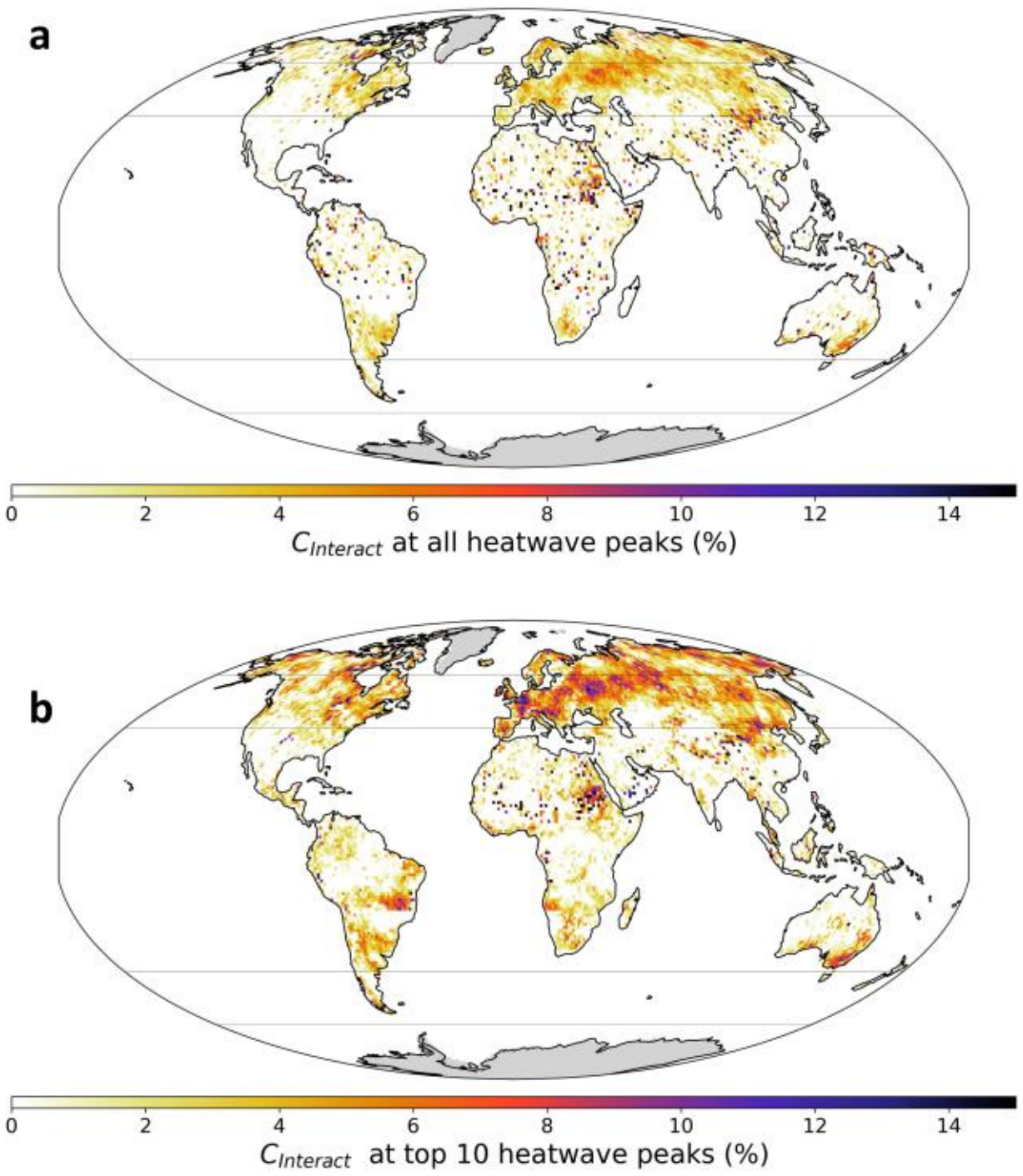

Fig. S18.

Relative contribution of the Z500–SM–T interaction to extreme heatwave peaks. Relative contribution of the Z500–SM–T interaction ($C_{interact}$) to (**a**) all summer heatwave peaks and (**b**) the top 10 most intense summer heatwave peaks over 1979–2023.

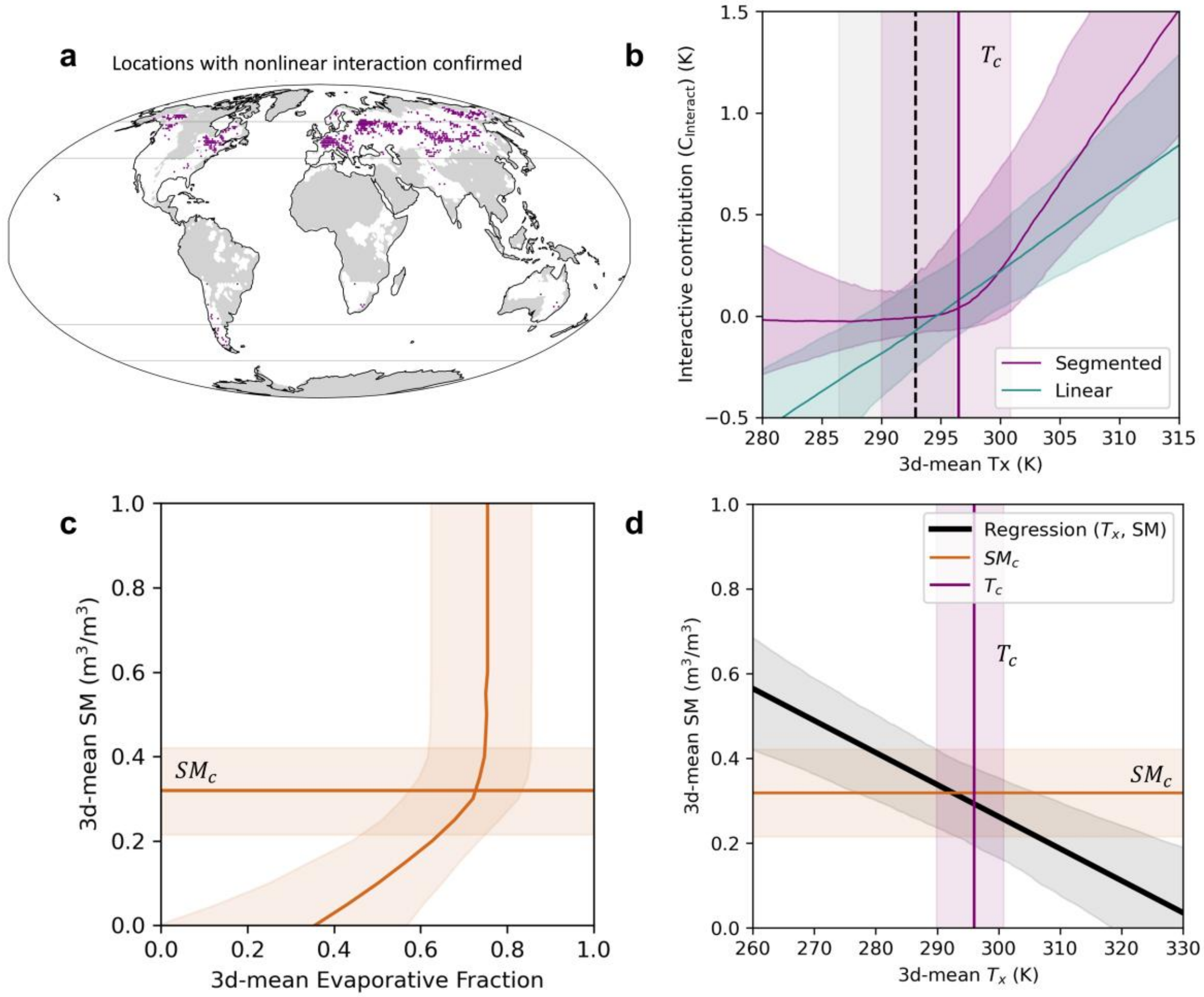

Fig. S19.

Co-occurrence of critical temperature and soil moisture in midlatitude humid regions. (a) Grid points exhibiting robust nonlinear behavior, defined by $\Delta\text{AIC}_{interact} < -2$ and confirmed in ≥99% of annual-replacement bootstrap realizations (see Methods). **(b-d)** Spatially-composited regression analyses over 1979–2023 of grid points shown in panel (a) within midlatitude humid regions (aridity index 0.65–1): **(b)** segmented regression between $C_{Interact}$ and T_x during all heatwave periods; **(c)** segmented regression between SM and EF during all dry-down periods; **(d)** linear regression between T_x and SM across all summer days. T_c and SM_c are identified separately from the $C_{Interact} - T_x$ regression (panel b) and the $SM - EF$ regression (panel c), respectively, and then visualized jointly in panel (d), where they intersect along the summer-wide $T_x - SM$ relationship, indicating coincident threshold crossing of the critical temperature and critical soil-moisture under typical summer conditions.

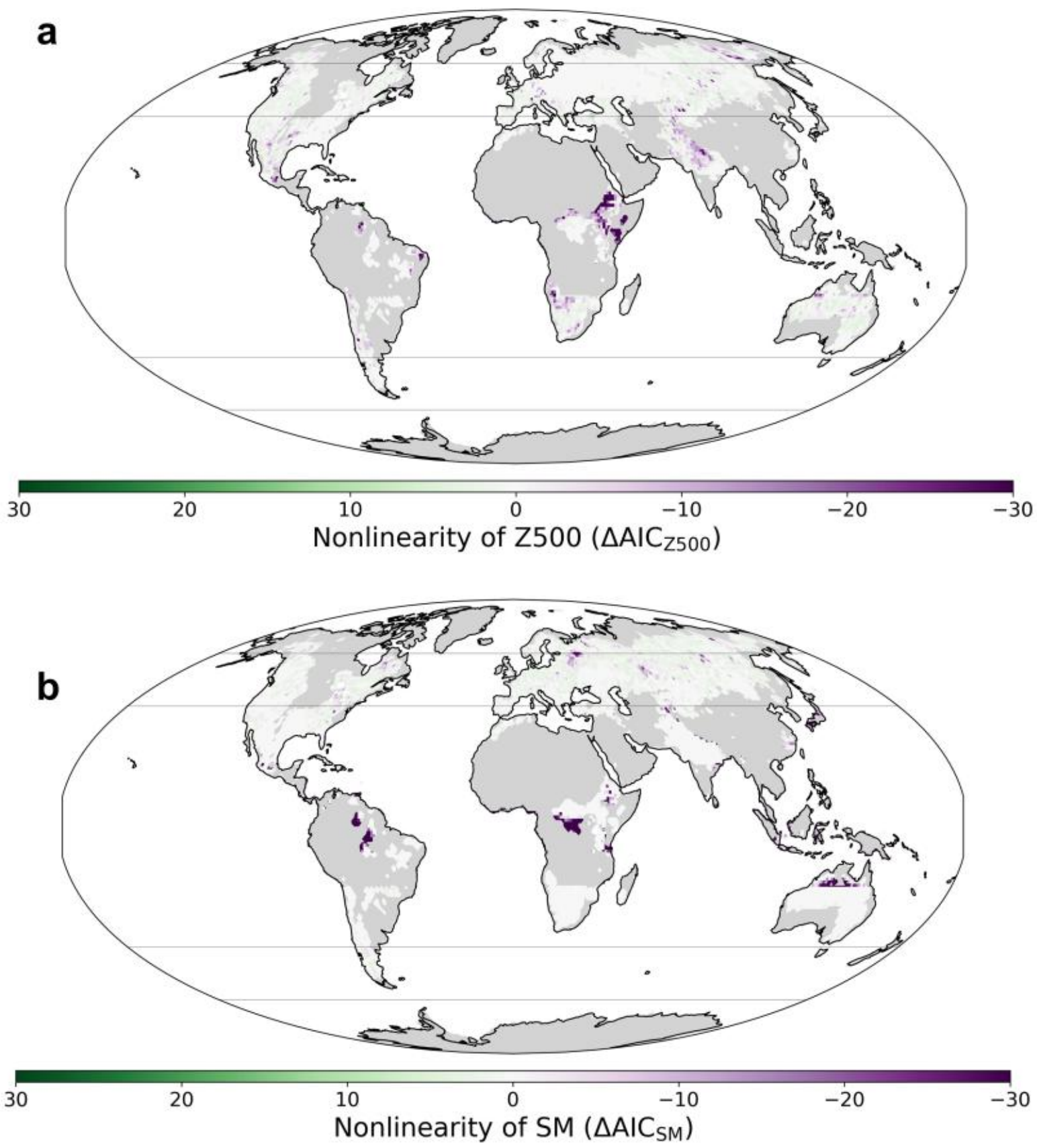

Fig. S20.

Nonlinearity of direct contributions from circulation and soil moisture. Nonlinearity magnitude (ΔAIC) of **(a)** the direct contribution from Z500 (ΔAIC$_{z500}$) and **(b)** the direct contribution from soil moisture (ΔAIC$_{SM}$) during summer heatwave periods (1979–2023). Black dots indicate grid points exhibiting robust nonlinear behavior ($\Delta AIC < -2$, confirmed in ≥99% of annual-replacement bootstrap realizations; see Methods). Grey shading denotes regions where the machine-learning model explains less than 60% of local temperature variance ($R^2_{ML} < 0.6$; see Methods). Grey horizontal lines mark the midlatitude band (40°–60° N/S).

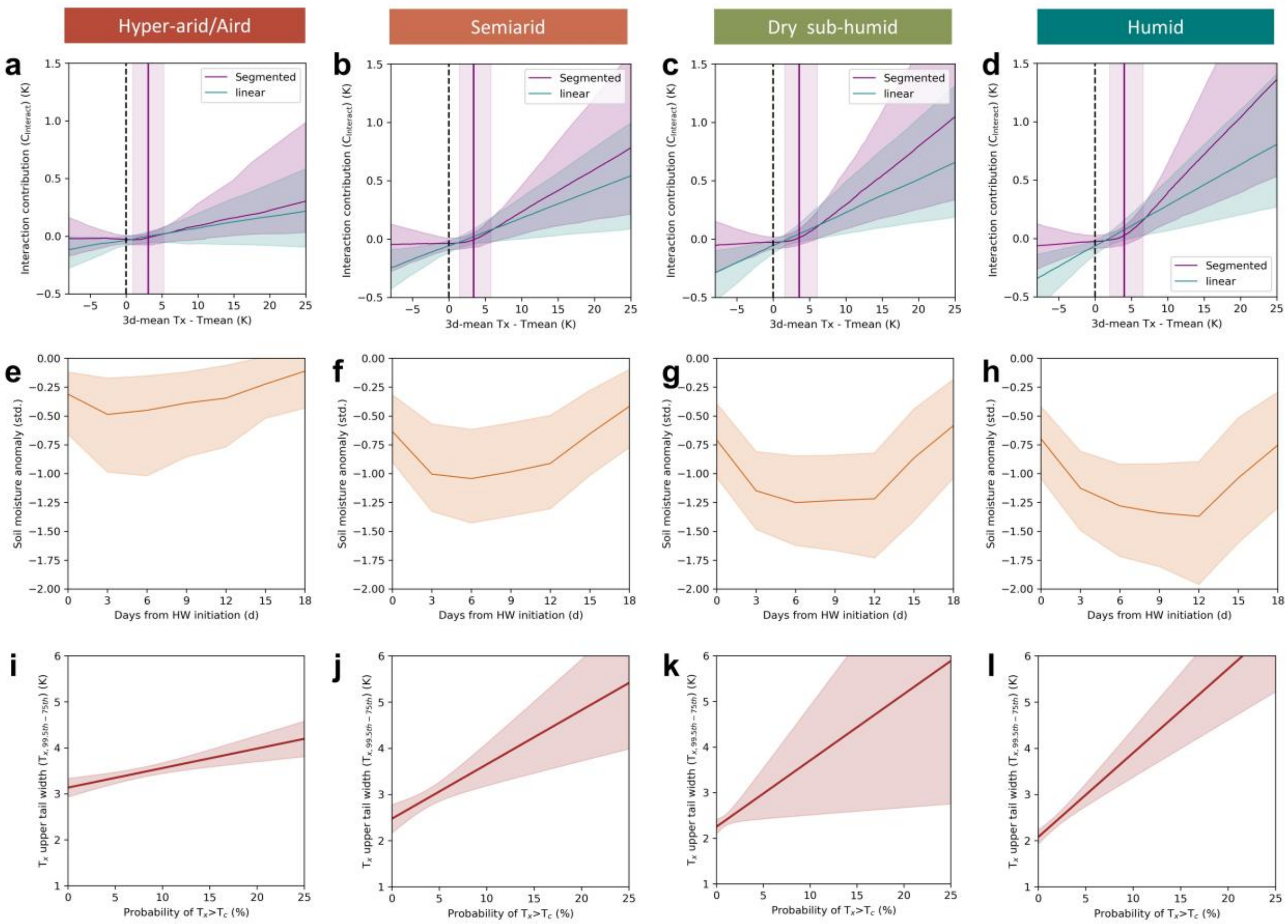

Fig. S21.

Aridity-dependent structure of nonlinear Z500–SM–T interactions. (**a-d**) Spatially composited nonlinear relationships between $C_{Interact}$ and T_x during summer heatwave periods (1979–2023), stratified by aridity index. (**e-h**) Standardized soil-moisture anomalies during heatwave periods by aridity class. (**i-l**) Linear regressions between the 5-year moving-window upper-tail width of temperature ($T_{x,99.5th} - T_{x,75th}$) and the exceedance probability of the critical temperature threshold (probability of $T_x > T_{c,interact}$). Shaded envelopes indicate the 5th–95th percentile spatial spread.

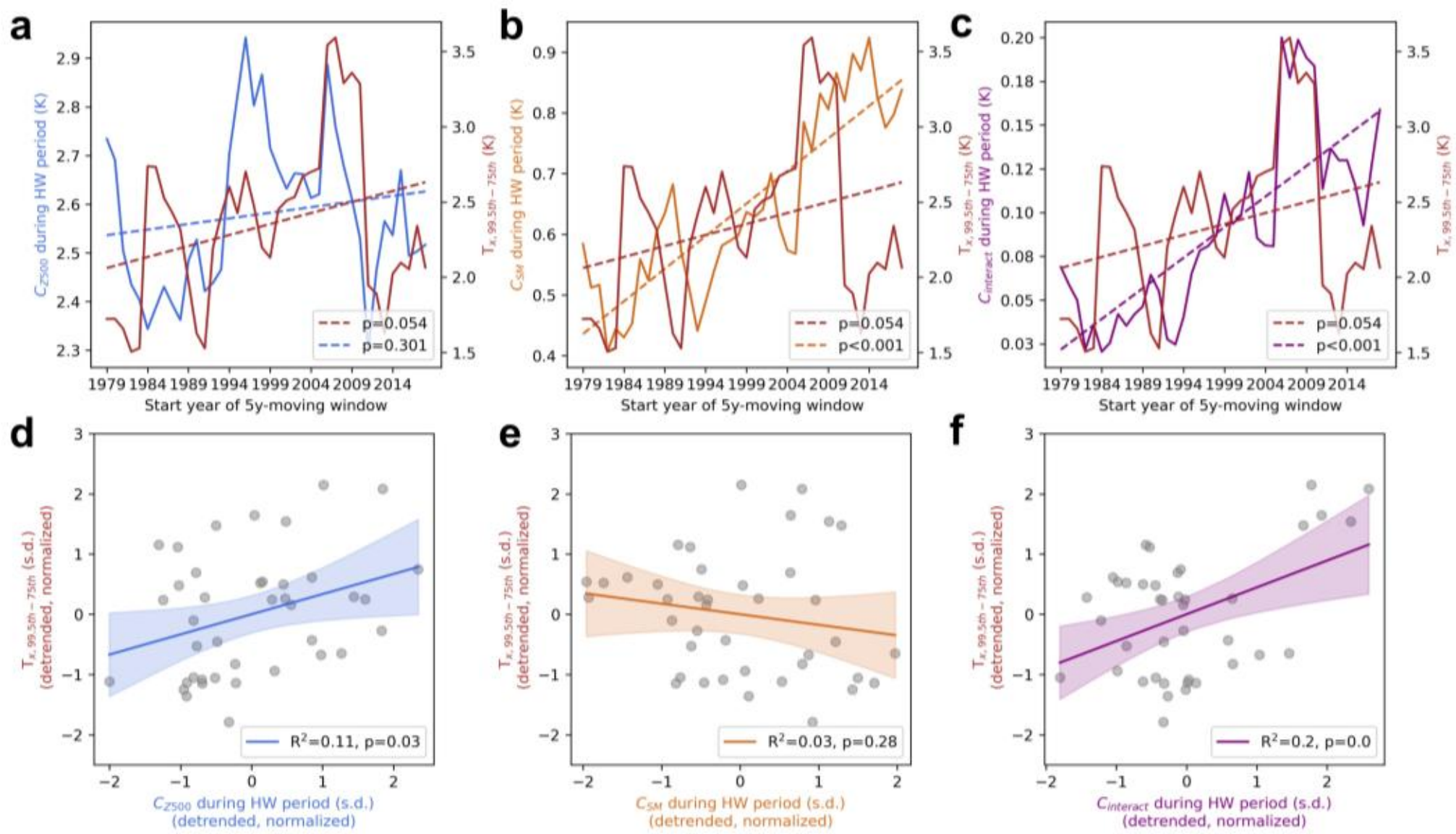

Fig. S22.

Associations between upper-tail temperature variability and circulation, soil moisture, and interaction contributions over midlatitude humid zones (40°–60°, aridity index ranges from 0.65 to 1.0). (**a**–**c**) Temporal evolution of the 5-year moving-window upper-tail width of daily maximum temperature ($T_{x,99.5th} - T_{x,75th}$) and the corresponding 5-year moving-window mean contributions from (**a**) mid-tropospheric geopotential height (C_{Z500}), (**b**) surface soil moisture (C_{SM}), and (**c**) their interaction ($C_{interact}$). (**d**–**f**) Linear regressions between the 5-year moving-window upper-tail width of T_x and the corresponding 5-year moving-window mean contributions from (**d**) C_{Z500}, (**e**) C_{SM}, and (**f**) $C_{interact}$ during heatwave periods. In panels (d, e, f), all time series are detrended and normalized prior to regression.

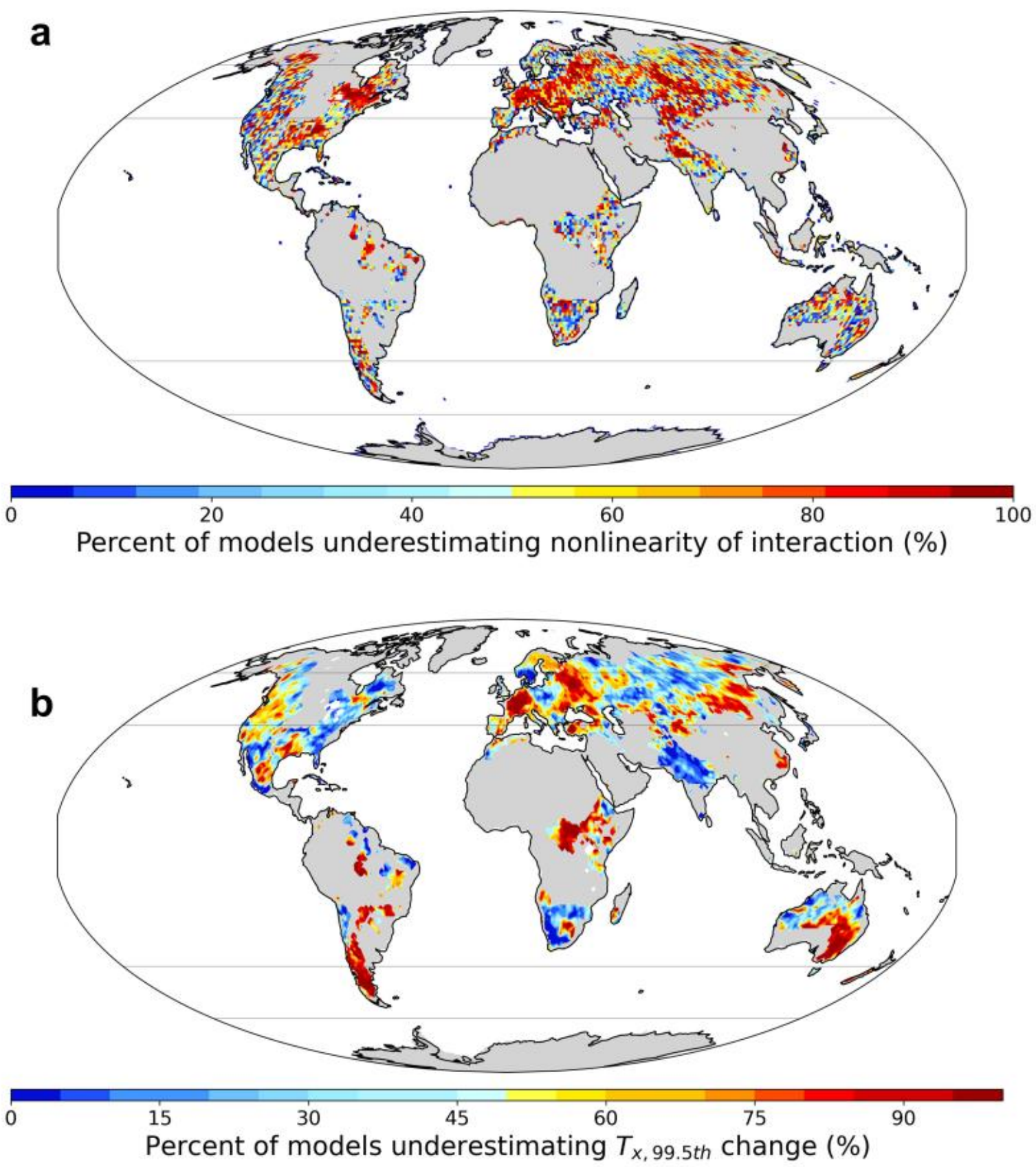

Fig. S23.

Percentage of 12 selected CMIP6 models underestimating (**a**) the nonlinearity magnitude of the interaction contribution ($\Delta AIC_{interact}$) during summer heatwave periods (1979–2023) and (**b**) changes in extreme summer temperature ($T_{x,99.5th}$) between 2004–2023 and 1979–1998. Grey shading indicates regions with $R^2_{ML} < 0.6$, and grey lines denote the midlatitude band (40°–60°).

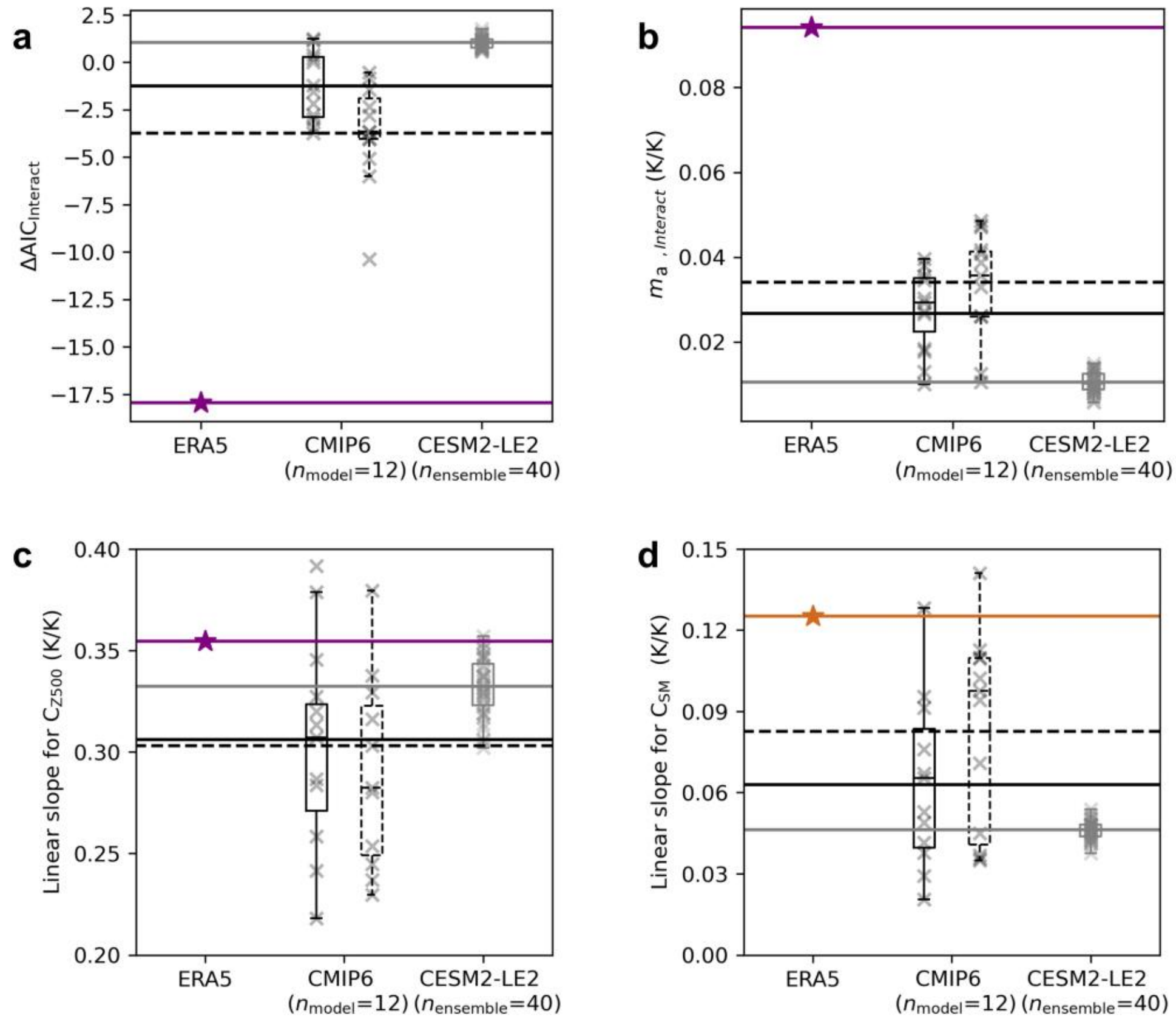

Fig. S24.

Model biases in temperature attribution diagnostics during heatwaves. Differences between ERA5, CMIP6 models, and CESM2-LE2 ensembles at grid points exhibiting robust nonlinear interaction: (**a**) nonlinearity magnitude ($\Delta AIC_{interact}$), (**b**) activated-regime slope (m_a) of $C_{Interact} - T_x$ relationship, (**c**) linear slope of $C_{Z500} - T_x$ relationship, and (**d**) linear slope of $C_{SM} - T_x$ relationship during summer heatwave periods (1979–2023). Metrics are computed per grid point and summarized per model/ensemble using the spatial median. Boxes show the interquartile range, whiskers denote the 5th–95th percentile, and horizontal lines indicate medians. For CMIP6 outputs, solid black boxes and horizontal lines denote estimates based on grid points exhibiting robust nonlinear interactions in observations ($R^2_{ML,obs} > 0.6, \Delta AIC_{interact,obs} < -2$), whereas dashed black boxes and lines denote estimates restricted to locations where the machine-learning model performs well and strong nonlinear behavior is detected in each model ($R^2_{ML,CMIP6} > 0.6, \Delta AIC_{interact,CMIP6} < -2$). This comparison shows that the bias in nonlinear interactions remains robust under this restriction, with only a slight improvement.

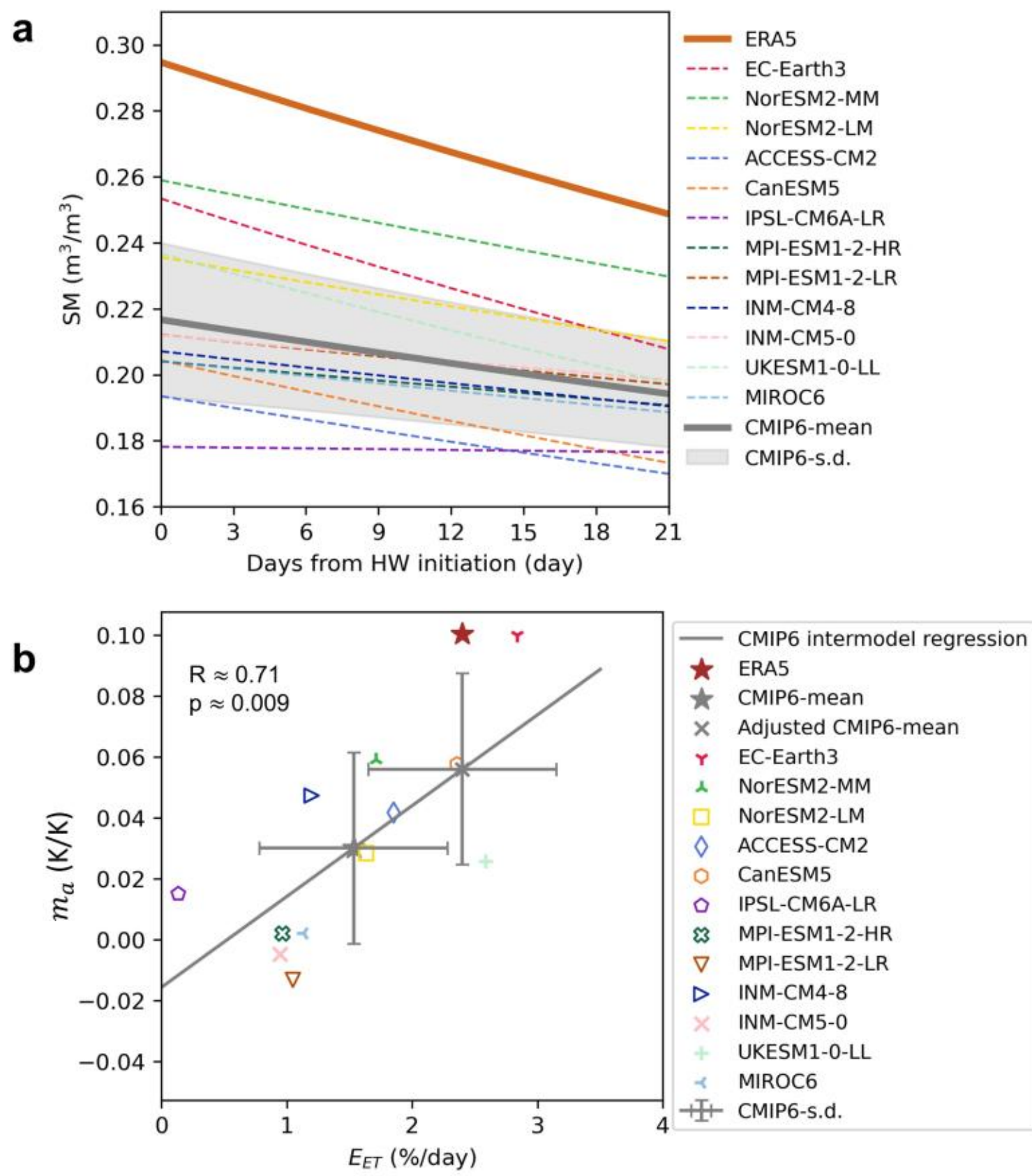

Fig. S25.

Soil-moisture drying dynamics and their link to nonlinear interaction strength. (a) Soil-moisture evolution within 21 days after heatwave onset in Central Europe during 1979–2023, comparing ERA5 with CMIP6 models. **(b)** Inter-model regression between the soil-moisture decline rate within 21 days after heatwave onset (E_{ET}) and the activated-regime slope of the interaction–temperature relationship (m_a) across CMIP6 models. The adjusted CMIP-mean represents the value estimated from the intermodel regression across CMIP6 models, using the corresponding ERA5 value as the predictor.

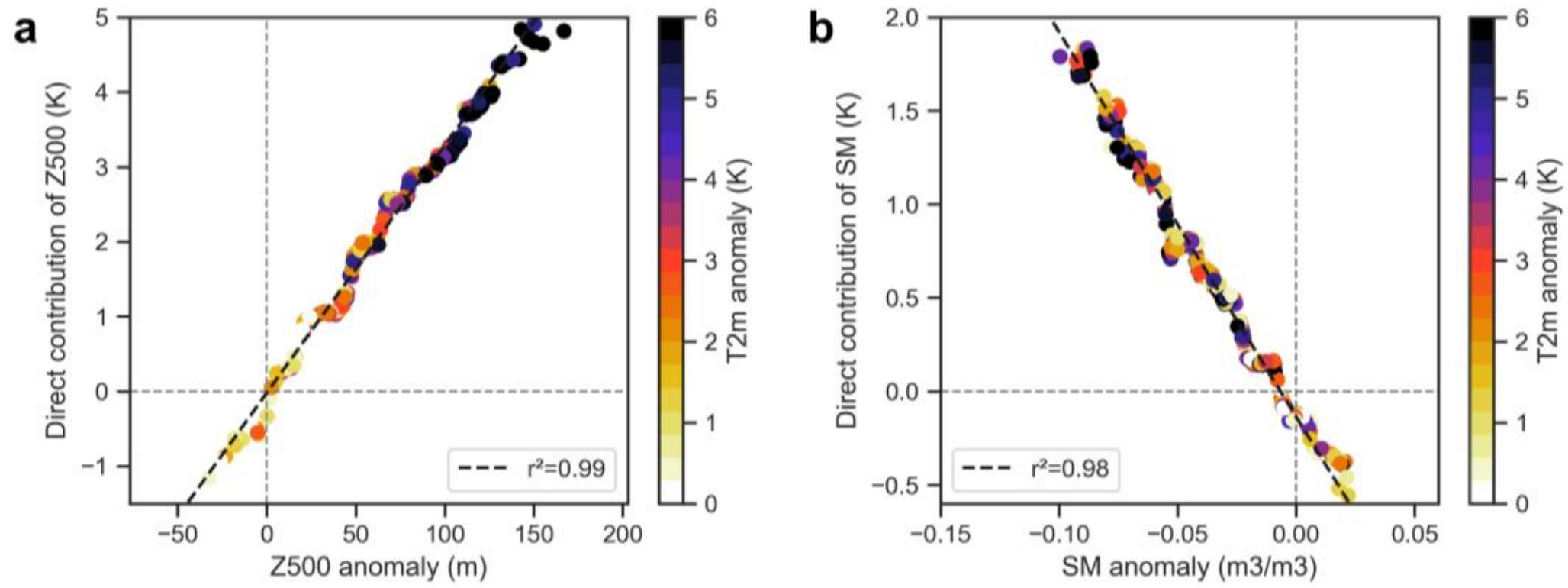

Fig. S26.

Correlation between XAI-derived contributions and observed anomalies. Correlations between XAI-derived contributions and observed anomalies of **(a)** mid-tropospheric geopotential height (C_{Z500}) and **(b)** soil moisture (C_{SM}) in Central Europe during summer heatwave periods (1979–2023).

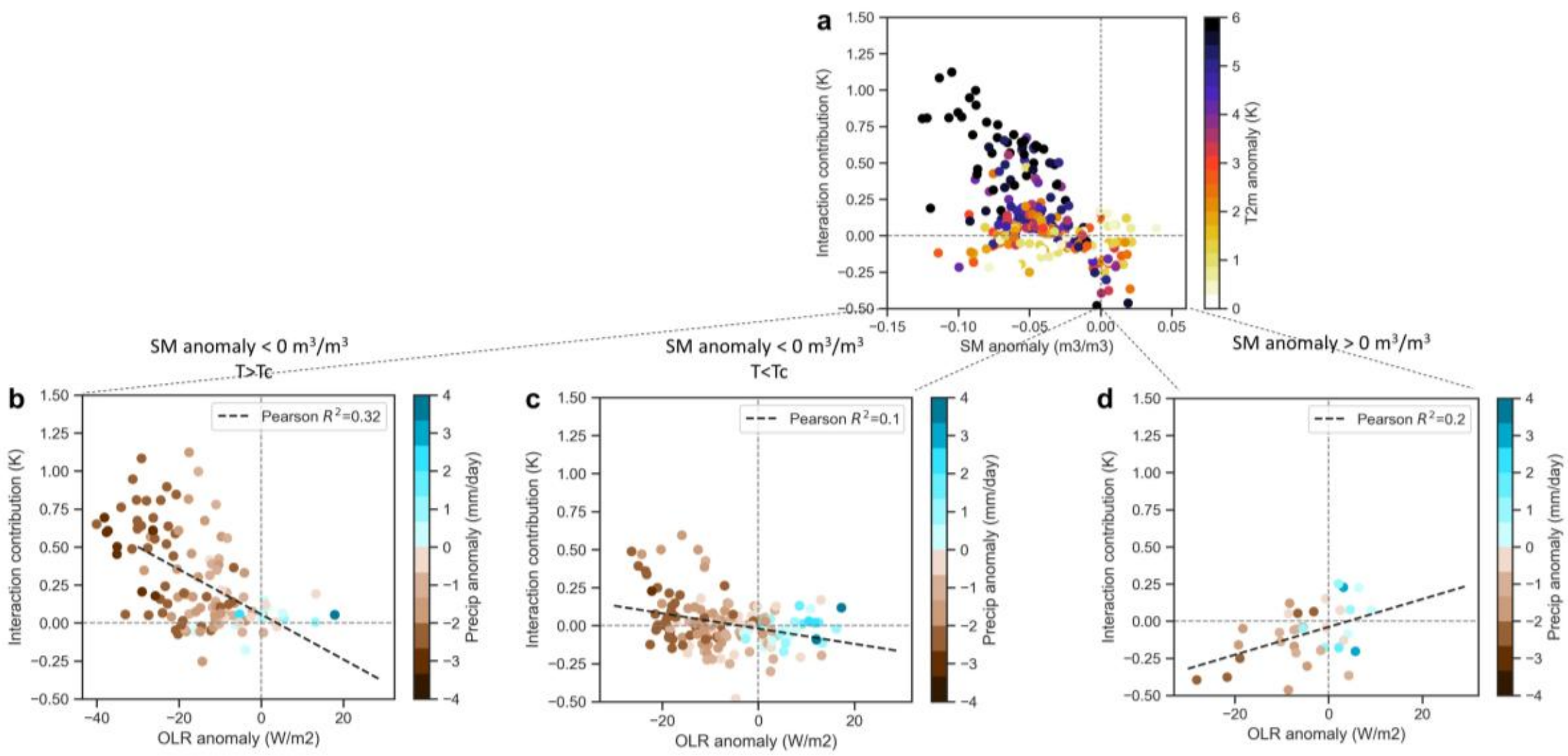

Fig. S27.

Interaction contribution in relation to convection under different hydrothermal conditions. Relationships between the interaction contribution ($C_{interact}$) and convective activity in Central Europe during heatwave periods (1979–2023): **(a)** correlation between $C_{interact}$ and soil-moisture anomaly; **(b–d)** correlations between $C_{interact}$ and outgoing longwave radiation anomaly under **(b)** SM anomaly < 0 and $T_x > T_c$, **(c)** SM anomaly < 0 and $T_x < T_c$, and **(d)** SM anomaly > 0 states. Positive OLR anomalies indicate enhanced downward longwave radiation and increased convection.

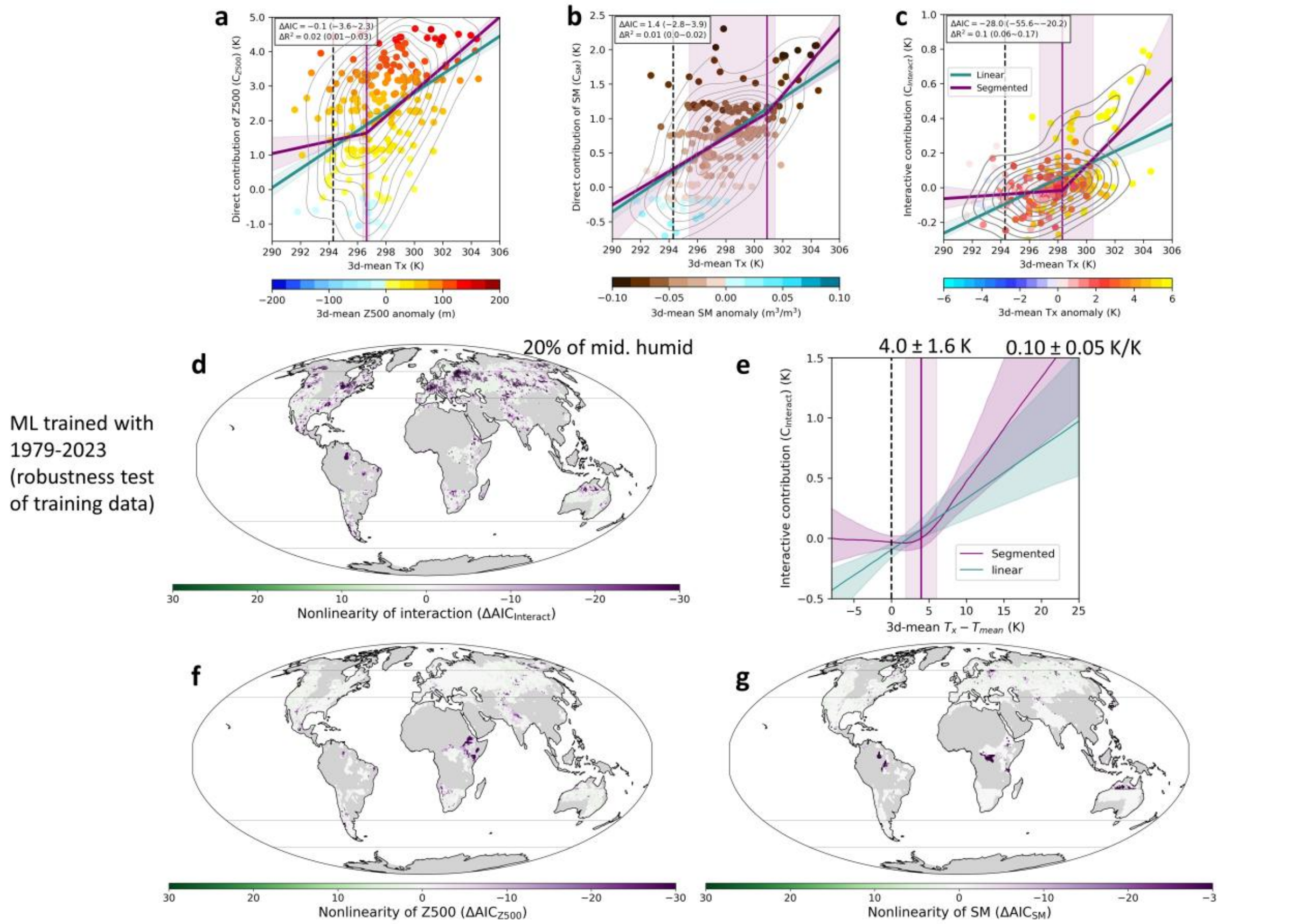

Fig. S28.

Robustness of nonlinear interaction diagnostics to the machine-learning training-period length. Robustness tests using a shortened training period (1979–2023) for the gradient-boosting regression (GBR) model, compared with the main setting (1950–2023). **(a–c)** Central Europe: segmented and linear regressions between T_x and the corresponding SHAP-derived contributions from **(a)** mid-tropospheric geopotential height (C_{Z500}), **(b)** surface soil moisture (C_{SM}), and **(c)** and their interactions ($C_{Interact}$) during all summer heatwave periods. (**d–g**) Global-scale diagnostics: **(d)** spatial distribution of the nonlinearity of the Z500–SM–T interaction, quantified by $\Delta\text{AIC}_{interact}$, during summer heatwave periods. Black dots indicate grid points exhibiting robust nonlinear behavior ($\Delta\text{AIC}_{interact} < -2$ and confirmed in ≥99% of annual-replacement bootstrap realizations; see Methods), and grey shading denotes regions where the machine-learning model explains less than 60% of local temperature variance ($R^2_{ML} < 0.6$). **(e)** Spatially composited relationship between the interactive contribution ($C_{Interact}$) and local temperature anomaly (T_x) at grid points identified as nonlinear in panel (d). Solid lines show the spatial median response and shaded envelopes indicate the 10th–90th percentile range across locations; temperature anomalies are expressed relative to the local summer mean. **(f–g)** Nonlinearity magnitude (ΔAIC) of the direct contributions from **(f)** Z500 (ΔAIC_{z500}) and **(g)** soil moisture (ΔAIC_{SM}) during summer heatwave periods. The results remain qualitatively consistent with the main training period, including robust nonlinear interactions over ~20% of midlatitude humid land (d), a critical temperature of 4.0 ± 1.6 K (e), and an activated-regime slope of 0.10 ± 0.05 K K^{-1} (e).

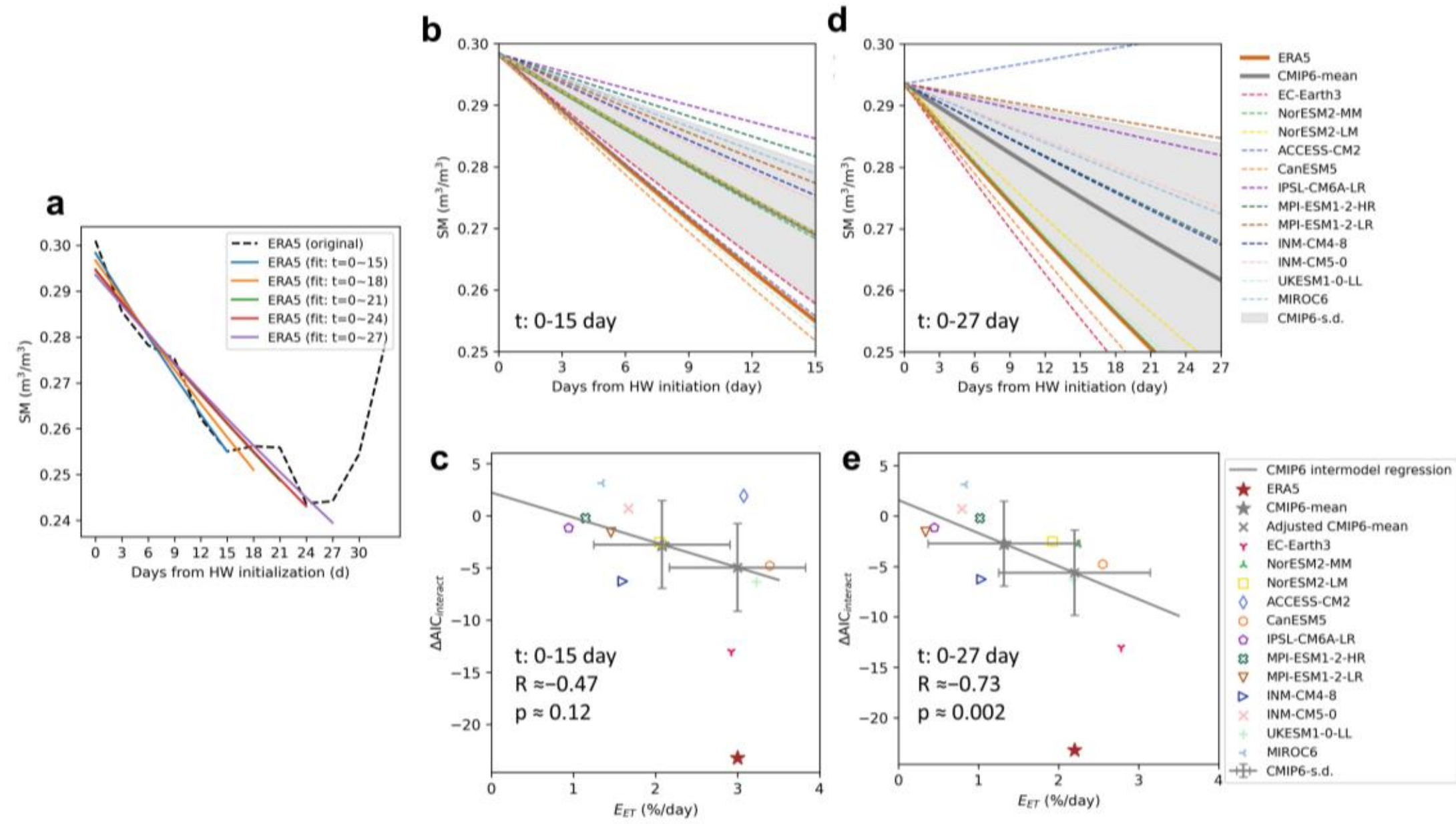

Fig. S29.

Sensitivity of soil-moisture drying rates to analysis window length. (a) Soil-moisture evolution during summer heatwave periods (1979–2023) in ERA5 and 12 CMIP6 models over Central Europe, with linear fits computed using different post-onset window lengths. **(b–e)** Soil-moisture decline rates following heatwave onset in ERA5 and CMIP6 models **(b, d)**, calculated assuming a common observed initial soil moisture at heatwave onset (see Methods), and their relationship to the nonlinearity of the Z500–SM–T interaction ($\Delta AIC_{Interact}$) **(c, e)**. Panels **(b, c)** show results using a 15-day window and panels **(d, e)** using a 27-day window. In (c, e), points denote individual CMIP6 models and lines indicate the inter-model linear regression between the soil-moisture decline rate (E_{ET}) and $\Delta AIC_{Interact}$. The adjusted CMIP-mean represents the value estimated from the intermodel regression across CMIP6 models, using the corresponding ERA5 value as the predictor.

Tables

Table S1.

Land area exhibiting nonlinear behavior among different statistic criterion and tests
Fraction of land area exhibiting nonlinear Z500–SM–T interaction under different statistical thresholds (ΔAIC < −2 or ΔAIC < 0), bootstrap resampling schemes (annual-, 10%-, or, 20%-replacement), and statistic tests (based on ΔAIC, LRT, or slope difference). Results demonstrate that identified nonlinear interactions are robust to criterion choice, resampling strategy, and statistical tests, with the highest prevalence in humid midlatitude regions.

Statistic criterion	Global Mid-Lat	North Mid-Lat	South Mid-Lat	**Mid-Lat humid**
**ΔAIC < -2**	**15%**	**19%**	**11%**	**28%**
Annual replacement ($\Delta AIC_{UCI,annual-re}$< -2)	12%	14%	9%	**21%**
10% replacement ($\Delta AIC_{UCI,90\%-re}$< -2)	11%	12%	9%	**19%**
20% replacement ($\Delta AIC_{UCI,80\%-re}$< -2)	9%	9%	9%	**15%**
ΔAIC < 0	16%	20%	11%	**30%**
Annual replacement ($\Delta AIC_{UCI,annual-re}$< 0)	14%	17%	11%	**25%**
10% replacement ($\Delta AIC_{UCI,90\%-re}$< 0)	13%	15%	10%	**24%**
20% replacement ($\Delta AIC_{UCI,80\%-re}$< 0)	10%	12%	9%	**19%**
Likelihood ratio test ($p < 0.05$)	16%	20%	11%	**29%**
Significant slope difference in segmented regression ($p < 0.05$)	17%	21%	11%	**30%**
